# Bridged 1,2,4-Trioxolanes: SnCl_4_—Catalyzed Synthesis and an In Vitro Study against *S. mansoni*

**DOI:** 10.3390/molecules28134913

**Published:** 2023-06-22

**Authors:** Peter S. Radulov, Ivan A. Yaremenko, Jennifer Keiser, Alexander O. Terent’ev

**Affiliations:** 1 N. D. Zelinsky Institute of Organic Chemistry, Russian Academy of Sciences, Moscow 119991, Russia; 2 Department of Medical Parasitology and Infection Biology, Swiss Tropical and Public Health Institute, CH-4123 Allschwil, Switzerland; 3 University of Basel, CH-4003 Basel, Switzerland

**Keywords:** peroxide, ozonide, tin chloride, hydrogen peroxide, antischistosomal

## Abstract

A synthesis of bridged 1,2,4-trioxolanes (bridged ozonides) from 1,5-diketones and hydrogen peroxide catalyzed by SnCl_4_ was developed. It was shown that the ratio of target ozonides can be affected by the application of SnCl_4_ as a catalyst and varying the solvent. A wide range of bridged 1,2,4-trioxolanes (ozonides) was obtained in yields from 50 to 84%. The ozonide cycle was moderately resistant to the reduction of the ester group near the peroxide cycle to alcohol with LiAlH_4_. The bridged ozonides were evaluated for their antischistosomal activity**.** These ozonides exhibited a very high activity against newly transformed schistosomula and adult *Schistosoma mansoni*.

## 1. Introduction

**Figure 1:**
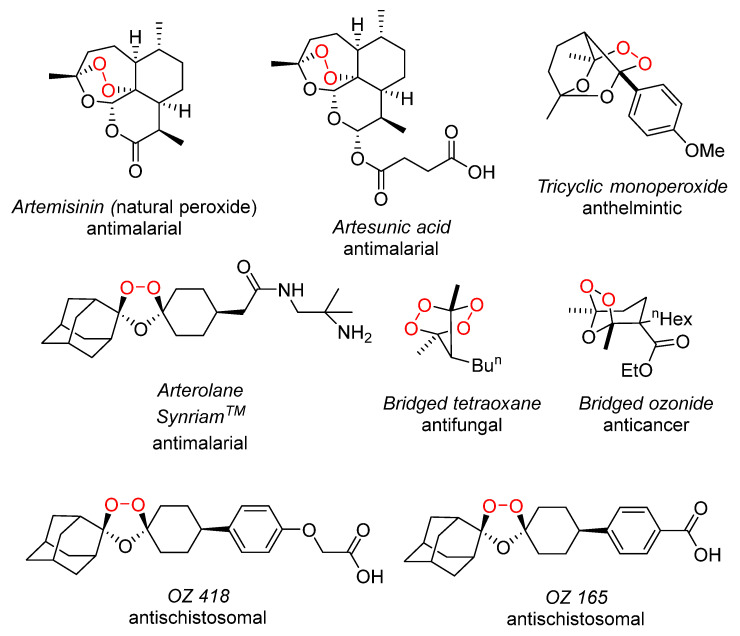
Structures of bioactive peroxides.

Organic peroxides related to the natural peroxide Artemisinin and its semisynthetic derivatives are a promising class of compounds for medicinal chemistry due to their antimalarial [1,2,3,4], antileishmanial [5,6], antischistosomal [7,8], anticancer [9,10,11], antifungal [12,13,14], antitubercular [15,16], and antiviral [17,18,19] activities (Figure 1). Among the synthetic peroxides, ozonides are the most attractive class. For example, synthetic ozonide OZ277 (“arterolane”) is used for the treatment of malaria. Additionally, this synthetic peroxide is active against α-coronavirus NL63, β-coronaviruses OC43, and SARS-CoV-2 [20,21,22]. Ozonides OZ418, OZ165, and their derivatives exhibit promising antischistosomal activity [23]. We found that bridged ozonides synthesized from β,δ’-triketones and H_2_O_2_ [24] also exhibit antischistosomal activity in vitro and in vivo [25]. It should be noted that bridged ozonides contain a bicyclic system related to that of natural Artemisinin. Semisynthetic artemisinins and their synthetic analogues, which are particularly active against juvenile schistosomes, have promise for the prevention of schistosomiasis. Schistosomiasis is a neglected tropical disease caused by parasitic flatworms (blood flukes) of the genus *Schistosoma* [26]. It affects more than 250 million people each year, mostly children from poor tropical rural areas, with praziquantel being the only therapy available. In the event of resistance, the treatment of schistosomiasis would be at risk [27,28]. Moreover, praziquantel, the only drug available for the treatment of schistosomiasis, is not effective against the juvenile stages of the parasite [29,30].

Carbonyl compounds, hydrogen peroxide, and hydroperoxides are convenient and important reagents for the synthesis of organic peroxides. On the basis of this, approaches to the synthesis of bis-hydroperoxides [31,32,33,34,35,36,37], bis-peroxides [38,39], 1,2,4,5-tetraoxanes [40,41,42,43,44], cyclic triperoxides [45,46], tricyclic monoperoxides [47,48], peroxylactones [49,50], and aminoperoxides [51,52,53,54,55,56,57,58,59] have been developed. However, approaches to the synthesis of 1,2,4-trioxolanes (ozonides) are limited. Traditionally, they are obtained via the ozonolysis of alkenes [60]. Another interesting approach to the synthesis of ozonides is the reaction of *O*-methyl oximes with carbonyl compounds in the presence of ozone (Griesbaum coozonolysis) [61]. The scarcity of approaches to the synthesis of ozonides constrains the structural diversity of the latter. Thus, it is time to tackle this issue, working out new concepts for the synthesis of ozonides. Recently, it was found that bridged ozonides can be obtained via the acid-catalyzed peroxidation of 1,5-diketones [62,63,64,65]. In our study, SnCl_4_ was shown to act as an efficient catalyst for the synthesis of bridged ozonides from 1,5-diketones and hydrogen peroxide. This finding expands the chemistry of peroxides and also provides new opportunities for the ozone-free synthesis of 1,2,4-trioxolanes. Bridged ozonides were tested for their antischistosomal activity against NTS (newly transformed schistosomula) and adult *S. mansoni*. 

## 2. Results and Discussion

1,5-Diketone **1a** was selected as a model substrate for the reaction with hydrogen peroxide. We evaluated the amount of SnCl_4_, H_2_O_2_, and the type of solvent on the assembly of peroxides **2a** and **3a** (Table 1). In the first stage, we chose 1.5 eq. of H_2_O_2_ and 1.0 eq. of SnCl_4_, with respect to diketone **1a** and THF as a solvent. A slight excess of hydrogen peroxide was chosen to achieve the complete conversion of diketone **1a**. The conversion of 1,5-diketone **1a** was monitored via TLC. The ratio of stereoisomeric ozonides **2a** and **3a** was established using NMR. After 24 h, the yield of peroxides **2a** and **3a** and the conversion of **1a** were 21% and 85%, respectively (entry 1, Table 1). In the case of using 3 eq. of SnCl_4_, the conversion of the diketone reached 100% in 24 h, and the yield of peroxides **2a** and **3a** was 64% (entry 2, Table 1). When using 5 eq. of SnCl_4_, the yield of ozonides **2a** and **3a** increased to 83% (**2a**:**3a** = 1:1) (entry 4, Table 1). A further increase in the amount of SnCl_4_ did not lead to an increase in the yield of the ozonides. In the case of using 3 eq. of H_2_O_2_, the yield of the ozonides was 70–71% when using both 3 eq. and 5 eq. of SnCl_4_ (entries 3 and 5, Table 1). Based on these results, the molar ratio of 1,5–diketone **1a**:H_2_O_2_:SnCl_4_ = 1:1.5:5.0 was chosen as optimal. Ozonides **2a** and **3a** were also obtained in high yield using 1,4-dioxane and Et_2_O as solvents (81% and 79%, respectively). However, the ratio of **2a**:**3a** was not 1:1 as in the case of entry 4 of Table 1, but 2.6:1.0 and 8.6:1.0, respectively (entries 6 and 7, Table 1). Thus, the ratio of ozonides can be tuned by varying the solvent to achieve the desired outcome. In cases of CH_3_CN and CH_2_Cl_2_, the formation of ozonides was not observed.

**Scheme 1:**
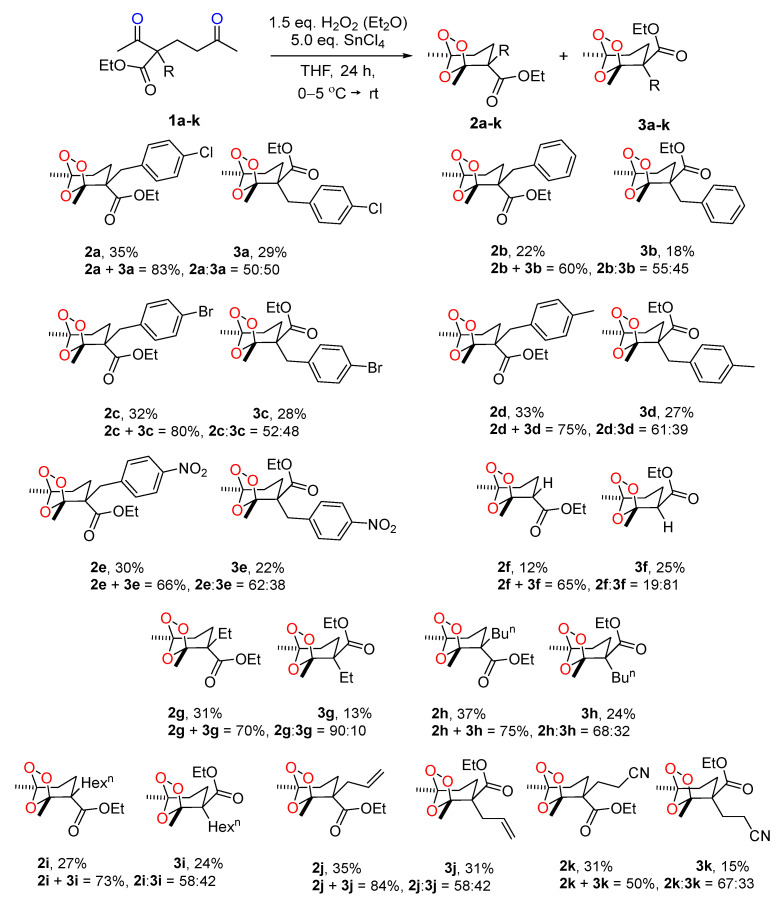
SnCl_4_—catalyzed synthesis of bridged 1,2,4-trioxolanes (ozonides). A 5.1 M solution of H_2_O_2-_ in Et_2_O (1.5 mol of Н_2_О_2_/1.0 mol of **1a**–**k**) and SnCl_4_ (5.0 mol of SnCl_4_/1.0 mol of **1a**–**k**) were added to a solution of 1,5-diketone **1a**–**k** (0.300 g, 0.81–1.49 mmol) in THF (5 mL) with stirring at 0–5 °С. The reaction mixture was warmed to 20–25 °С and stirred for 24 h. Isolated yields. The ratio of stereoisomeric ozonides was found by ^1^H NMR.

To further study their antischistosomal activity, bridged ozonides were synthesized under the best conditions (entry 4, Table 1). These conditions are the most suitable for obtaining each diastereosomer in individual form, since diastereosomers can behave differently in biological tests. With the optimal conditions in hand, we explored the scope and limitations of the assembly of ozonides **2** and **3**. As demonstrated in Scheme 1, a series of ozonides, containing various functional groups and moieties, i.e., an alkene, nitrile, ester, or aromatic core, could be obtained in good to excellent yields. Interestingly, the alkene function remained unchanged under the reaction conditions and the possible products of epoxidation were not detected. The yields were in the range from 50% (for ozonides from diketone **1k**) to 84% (for ozonides from diketone **1j**). Peroxides **2a**–**k** and **3a**–**k** were separated using ordinary column chromatography. Surprisingly, under the action of tin chloride, the ozonides were selectively formed, rather than a complex inseparable mixture. Under the action of SnCl_4_, the resulting peroxides could potentially undergo transformation with the cleavage of the O-O bond. Thus, we discovered a new nature of SnCl_4_ and new directions for the use of SnCl_4_ in peroxide chemistry.

**Scheme 2:**
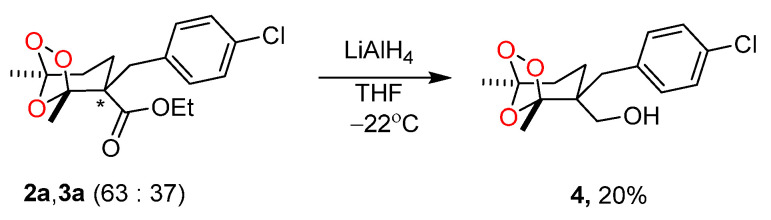
The reduction of the ester group in ozonides.

In the present study, it was found that the reduction of the ester group in ozonides **2a** and **3a** at −22 °C occurred with the formation of ozonide **4** with a primary alcohol functional group (Scheme 2). However, at −78 °C, the reduction of this ester group did not occur [62,63]. Additionally, at room temperature, both the ester group and peroxide cycle were reduced. The ozonide cycle of peroxide **2a** turned out to be moderately resistant to LiAlH_4_ under these conditions, but at the same time, peroxide **3a** turned out to be very sensitive. On the other hand, such a different sensitivity of the ozonides made it possible to obtain only one diastereoisomer from a mixture of initial ozonides, albeit with a yield of 20%. Such a transformation can open up new possibilities for expanding the structural diversity of peroxides.

### In Vitro Drug Assay on Newly Transformed Schistosomula (NTS) and Adult Schistosomes

In order to search for new agents against *Schistosoma mansoni* based on organic peroxides, a preliminary evaluation of the synthesized ozonides was carried out. Twenty synthesized bridged ozonides, **2b**–**k** and **3b**–**k,** were tested for their antischistosomal activity against the larval schistosome stage. At the highest drug concentration (33.3 µM), 11 compounds (**2b**–**f**, **2h**–**k**, **3c**, **3h,** and **3j**) were highly effective against NTS (newly transformed schistosomula) (effect > 90%). In most cases, isomer **2** was more active than isomer **3**. At 10 µM, only seven of them (**2b**–**e**, **2i**, **3c**, and **3h**) (Table 2) still showed a moderate activity (effect > 50%) and progressed into being tested against adult worms. Among these seven compounds, only four, **2c**, **2d**, **2i,** and **3c,** respectively, were moderately active against adult schistosomes (effect > 50% at 10 µM). A high activity (>70%) against adult schistosomes was observed with compounds **2c**, **2d,** and **3c**. We compared our results with two reference compounds, praziquantel (the drug of choice) and artesunate (a key antimalarial peroxide). Compounds **2d**, **2e**, **2j,** and **3c** were more active than artesunate and praziquantel against newly transformed schistosomula. In comparison to artesunate, which is not active against adult schistosomes in vitro (IC_50_ > 38 μM [66]), several compounds were active against this stage of the parasite. The results obtained demonstrated that this antischistosomal activity is highly dependent on the ozonide isomer structure and type of substituent in the bridge. This may indicate that ozonides do not act as oxidizers. It is likely that, by varying the substituents in the benzene ring, it will be possible to achieve promising results in the future.

## 3. Materials and Methods

### 3.1. General Materials and Methods

Caution: precautions should be taken when working with peroxides, such as the use of protective screens, fume hoods, and the avoidance of the contact of peroxides with transition metal salts, heat, and shaking.

The NMR spectra were recorded on a commercial instrument (300.13 MHz for ^1^Н, 75.48 MHz for ^13^С) in СDCl_3_. High-resolution mass spectra (HRMS) were acquired on a Brucker micrOTOF II instrument using electrospray ionization (ESI). The measurements were performed in positive ion mode (interface capillary voltage of 4500 V); the mass ratio was from *m/z* 50 to 3000; and the external/internal calibration was performed using Electrospray Calibrant Solution. A syringe injection was used for the solutions in MeCN (flow rate of 3 μL/min). Nitrogen was applied as a dry gas and the interface temperature was set at 180 °C. IR spectra were recorded on a Bruker ALPHA spectrometer.

The TLC analysis was carried out on silica gel chromatography plates of Macherey-Nagel Alugram UV254. The sorbent: Silica 60, a specific surface (BET) of ~ 500 m^2^/g, a mean pore size of 60 Å, a specific pore volume of 0.75 mL/g, and a particle size of 5–17 µm. The binder: a highly polymeric product stable in almost all the organic solvents and resistant towards aggressive visualization reagents. The melting points were determined using Kofler hot-stage apparatus. The chromatography of the 1,5-diketones was performed on silica gel (0.060–0.200 mm, 60 A, CAS 7631-86-9). The chromatography of the ozonides was performed on silica gel (0.040–0.060 mm, 60 A, CAS 7631-86-9).

SnCl_4_, H_2_O_2_ (35% aq.), and MgSO_4_, were purchased from commercial sources and used as received. An ethereal solution of H_2_O_2_ in Et_2_O (5.1 M) was prepared via an extraction with Et_2_O (5 × 100 mL) from a 35% aqueous solution (100 mL), followed by drying over MgSO_4_. Then, part of Et_2_O was removed in the vacuum of a membrane vacuum pump at 20–25 °C and titrated iodometrically [12,67]. All the solvents were distilled before use, using standard procedures. 

### 3.2. Synthesis of Starting Compounds

1,5-Diketones **1a [12]** and **1b**–**k [63]** were synthesized according to known procedures. 

### 3.3. Procedure for the Synthesis of Ozonides ***2a*** and ***3a*** from 1,5-Diketone ***1a***, for Table 1

An ethereal solution of H_2_O_2_ in Et_2_O (5.1 M) (0.27–0.54 mL, 1.4–2.8 mmol, and 1.5–3.0 mol of Н_2_О_2_/1.0 mol of **1a**) and SnCl_4_ (0.11–0.54 mL, 0.9–4.6 mmol, and 1.0–5.0 mol of SnCl_4_/1.0 mol of **1a**) was added to a solution of **1a** (0.30 g, 0.9 mmol) in dry THF (5 mL), with stirring at 0–5 °С. The reaction mixture was warmed to 20–25 °С and stirred for 24 h. Then, CHCl_3_ (40 mL) were added successively and the mixture was washed with water (10 mL), a saturated aq. sol. of NaHCO_3_ (10 mL), and then with water (2 × 10 mL). The organic phase was dried over MgSO_4_ and filtered. The solvent was removed in the vacuum of a water jet pump. 

Peroxides **2a** and **3a** were isolated using column chromatography on SiO_2_ with the use of a petroleum ether (PE):ethyl acetate (EA) mixture as the eluent, with a gradient of EA from 1 to 5 vol. %.

### 3.4. General Procedure for the Synthesis of Ozonides ***2a***–***k*** and ***3a***–***k*** from 1,5-Diketones ***1a***–***k***, for Scheme 1

An ethereal solution of H_2_O_2_ in Et_2_O (0.24–0.44 mL, 1.2–2.2 mmol, and 1.5 mol of Н_2_О_2_/1.0 mol of **1a**–**k**) and SnCl_4_ (0.47–0.87 mL, 4.1–7.5 mmol, and 5.0 mol of SnCl_4_/1.0 mol of **1a**–**k**) were added successively to a solution of **1a**–**k** (0.30 g, 0.8–1.5 mmol) in dry THF (5 mL), with stirring at 0–5 °С. The reaction mixture was warmed to 20–25 °С and stirred for 24 h. Then, CHCl_3_ (40 mL) was added and the mixture was washed with water (10 mL), a saturated aq. sol. of NaHCO_3_ (10 mL), and then with water (2 × 10 mL). The organic phase was dried over MgSO_4_ and filtered. The solvent was removed in the vacuum of a water jet pump. 

Peroxides **2a**–**k** and **3a**–**k** were isolated using column chromatography on SiO_2_ with the use of a PE:EA mixture as the eluent, with a gradient of EA from 1 to 5 vol. %.

**2a**: 110.1 mg, 0.32 mmol, and yield 35%; **3a**: 98.8 mg, 0.26 mmol, and yield 29%; **2b**: 69.6 mg, 0.22 mmol, and yield 22%; **3b**: 57.0 mg, 0.19 mmol, and yield 18%; **2c**: 100.2 mg, 0.26 mmol, and yield 32%; **3c**: 87.6 mg, 0.23 mmol, and yield 28%; **2d**: 104.2 mg, 0.32 mmol, and yield 33%; **3d**: 85.3 mg, 0.26 mmol, and yield 27%; **2e**: 94.4 mg, 0.27 mmol, and yield 30%; **3e**: 69.2 mg, 0.19 mmol, and yield 22%; **2f**: 38.9 mg, 0.18 mmol, and yield 12%; **3f**: 81.0 mg, 0.37 mmol, and yield 25%; **2g**: 99.5 mg, 0.40 mmol, and yield 31%; **3g**: 41.7 mg, 0.17 mmol, and yield 13%; **2h**: 117.9 mg, 0.43 mmol, and yield 37%; **3h**: 76.5 mg, 0.28 mmol, and yield 24%; **2i**: 85.6 mg, 0.28 mmol, and yield 27%; **3i**: 76.0 mg, 0.25 mmol, and yield 24%; **2j**: 112.0 mg, 0.44 mmol, and yield 35%; **3j**: 99.2 mg, 0.38 mmol, and yield 31%; **2k**: 98.9 mg, 0.37 mmol, and yield 31%; and **3k**: 47.8 mg, 0.18 mmol, and yield 15%. 

Mixtures of peroxides **2a**–**k** and **3a**–**k** were isolated using column chromatography on SiO_2_ with the use of a PE:EA mixture as the eluent, with a gradient of EA from 5 to 20 vol. %.

**2a** and **3a**: 251.8 mg, 0.74 mmol, and yield 80%; **2b** and **3b**: 190.0 mg, 0.62 mmol, and yield 60%; **2c** and **3c**: 237.4 mg, 0.65 mmol, and yield 80%; **2d** and **3d**: 236.8 mg, 0.74 mmol, and yield 75%; **2e** and **3e**: 207.5 mg, 0.59 mmol, and yield 66%; **2f** and **3f**: 210.6 mg, 0.97 mmol, and yield 65%; **2g** and **3g**: 224.7 mg, 0.92 mmol, and yield 70%; **2h** and **3h**: 239.0 mg, 0.88 mmol, and yield 75%; **2i** and **3i**: 231.3 mg, 0.77 mmol, and yield 73%; **2j** and **3j**: 268.8 mg, 1.04 mmol, and yield 84%; and **2k** and **3k**: 159.5 mg, 0.59 mmol, and yield 50%. 

Compounds **2a–k** and **3a–k** are known and were described in our previous studies [12,63]. 

#### 3.4.1. Ethyl (1R*,2R*,5S*)-2-(4-chlorobenzyl)-1,5-dimethyl-6,7,8-trioxabicyclo[3.2.1]octane-2-carboxylate (**2a**)

White crystals. Mp = 99–100 °С. (Lit. [12] Mp = 99–100 °С). R_f_ = 0.46 (TLC, PE:EA, 10:1). ^1^Н NMR (300.13 MHz, CDCl_3_), δ: 7.21 (d, *J* = 8.2 Hz, 2H), 7.00 (d, *J* = 8.2 Hz, 2H), 4.18 (q, *J* = 7.1 Hz, 2H), 3.31 (d, *J* = 12.9 Hz, 1H), 2.59 (d, *J* = 12.9 Hz, 1H), 2.12–1.97 (m, 2H), 1.79 (s, 3H), 1.82–1.53 (m, 2H), 1.48 (s, 3H), and 1.26 (t, *J* = 7.1 Hz, 3H). ^13^С NMR (75.48 MHz, CDCl_3_), δ: 172.3, 134.6, 132.9, 131.3, 128.5, 111.1, 109.9, 61.3, 54.2, 40.3, 32.9, 25.8, 20.6, 18.8, and 14.2. Anal. Calcd for C_17_H_21_ClO_5_: C, 59.91; H, 6.21; and Cl, 10.40. Found: C, 59.98; H, 6.27; and Cl, 10.49.

#### 3.4.2. Ethyl (1R*,2S*,5S*)-2-(4-chlorobenzyl)-1,5-dimethyl-6,7,8-trioxabicyclo[3.2.1]octane-2-carboxylate (**3a**)

White crystals. Mp = 89–90 °С. (Lit. [12] Mp = 89–90 °С). R_f_ = 0.40 (TLC, PE:EA, 10:1). ^1^Н NMR (300.13 MHz, CDCl_3_), δ: 7.23 (d, *J* = 8.3 Hz, 2H), 7.05 (d, *J* = 8.3 Hz, 2H), 4.14 (q, *J* = 7.1 Hz, 2H), 3.33 (d, *J* = 13.7 Hz, 1H), 3.00 (d, *J* = 13.7 Hz, 1H), 2.61 (td, *J* = 13.2, 6.5 Hz, 1H), 1.97–1.75 (m, 2H), 1.66 (s, 3H), 1.56 (s, 3H), 1.52–1.40 (m, 1H), and 1.23 (t, *J* = 7.1 Hz, 3H). ^13^С NMR (75.48 MHz, CDCl_3_), δ: 172.4, 135.8, 132.9, 131.4, 128.6, 111.3, 109.2, 61.4, 54.4, 37.0, 31.2, 21.8, 20.8, 19.1, and 14.2. Anal. Calcd for C_17_H_21_ClO_5_: C, 59.91; H, 6.21; and Cl, 10.40. Found: C, 59.99; H, 6.27; and Cl, 10.46.

#### 3.4.3. Ethyl (1R*,2R*,5S*)-2-benzyl-1,5-dimethyl-6,7,8-trioxabicyclo[3.2.1]octane-2-carboxylate (**2b**)

White crystals. Mp = 59–60 °С. (Lit. [63] Mp = 59–60 °С). R_f_ = 0.67 (TLC, PE:EA, 10:1). ^1^Н NMR (300.13 MHz, CDCl_3_), δ: 7.33–7.21 (m, 3H), 7.17–7.06 (m, 2H), 4.23 (q, *J* = 7.1 Hz, 2H), 3.38 (d, *J* = 12.9 Hz, 1H), 2.68 (d, *J* = 12.9 Hz, 1H), 2.15–1.97 (m, 2H), 1.86 (s, 3H), 1.89–1.59 (m, 2H), 1.52 (s, 3H), and 1.30 (t, *J* = 7.1 Hz, 3H). ^13^С NMR (75.48 MHz, CDCl_3_), δ: 172.5, 136.1, 130.0, 128.3, 126.9, 111.2, 109.9, 61.1, 54.3, 41.0, 32.9, 25.8, 20.6, 18.8, and 14.2. Anal. Calcd for C_17_H_22_O_5_: C, 66.65; and H, 7.24. Found: C, 66.69; and H, 7.28.

#### 3.4.4. Ethyl (1R*,2S*,5S*)-2-benzyl-1,5-dimethyl-6,7,8-trioxabicyclo[3.2.1]octane-2-carboxylate (**3b**)

White crystals. Mp = 42–43 °С. (Lit. [63] Mp = 42–43 °С). R_f_ = 0.63 (TLC, PE:EA, 10:1). ^1^Н NMR (300.13 MHz, CDCl_3_), δ: 7.33–7.20 (m, 3H), 7.18–7.08 (m, 2H), 4.18 (q, *J* = 7.1 Hz, 2H), 3.39 (d, *J* = 13.6 Hz, 1H), 3.08 (d, *J* = 13.6 Hz, 1H), 2.63 (td, *J* = 13.5, 6.3 Hz, 1H), 1.77–2.03 (m, 2H), 1.70 (s, 3H), 1.57 (s, 3H), 1.64–1.47 (m, 1H), and 1.23 (t, *J* = 7.1 Hz, 3H). ^13^С NMR (75.48 MHz, CDCl_3_), δ: 172.6, 137.3, 130.0, 128.4, 128.3, 126.9, 111.5, 109.2, 61.2, 54.4, 37.8, 31.2, 21.8, 20.9, 19.1, and 14.2. Anal. Calcd for C_17_H_22_O_5_: C, 66.65; and H, 7.24. Found: C, 66.72; and H, 7.31.

#### 3.4.5. Ethyl (1R*,2R*,5S*)-2-(4-bromobenzyl)-1,5-dimethyl-6,7,8-trioxabicyclo[3.2.1]octane-2-carboxylate (**2c**)

White crystals. Mp = 108–109 °С. (Lit. [63] Mp = 108–109 °С). R_f_ = 0.63 (TLC, PE:EA, 10:1). ^1^Н NMR (300.13 MHz, CDCl_3_), δ: 7.37 (d, *J* = 8.3 Hz, 2H), 6.95 (d, *J* = 8.3 Hz, 2H), 4.19 (q, *J* = 7.1 Hz, 2H), 3.30 (d, *J* = 12.9 Hz, 1H), 2.58 (d, *J* = 12.9 Hz, 1H), 1.79 (s, 3H), 2.14–1.89 (m, 2H), 1.84–1.54 (m, 2H), 1.48 (s, 3H), and 1.26 (t, *J* = 7.1 Hz, 3H). ^13^С NMR (75.48 MHz, CDCl_3_), δ: 172.3, 135.2, 131.7, 131.5, 121.0, 111.0, 109.9, 61.3, 54.1, 40.4, 32.9, 25.7, 20.6, 18.7, and 14.2. Anal. Calcd for C_17_H_21_BrO_5_: C, 53.00; H, 5.49; and Br, 20.74. Found: C, 53.09; H, 5.57; and Br, 20.79.

#### 3.4.6. Ethyl (1R*,2S*,5S*)-2-(4-bromobenzyl)-1,5-dimethyl-6,7,8-trioxabicyclo[3.2.1]octane-2-carboxylate (**3c**)

White crystals. Mp = 104–105 °С. (Lit. [63] Mp = 104–105 °С).R_f_ = 0.59 (TLC, PE:EA, 10:1). ^1^Н NMR (300.13 MHz, CDCl_3_), δ: 7.37 (d, *J* = 8.3 Hz, 2H), 6.98 (d, *J* = 8.3 Hz, 2H), 4.14 (q, *J* = 7.1 Hz, 2H), 3.31 (d, *J* = 13.6 Hz, 1H), 2.99 (d, *J* = 13.6 Hz, 1H), 2.62 (td, *J* = 13.2, 6.6 Hz, 1H), 1.96–1.73 (m, 2H), 1.66 (s, 3H), 1.52–1.40 (m, 1H), 1.55 (s, 3H), and 1.22 (t, *J* = 7.1 Hz, 3H). ^13^С NMR (75.48 MHz, CDCl_3_), δ: 172.4, 136.3, 131.8, 131.6, 121.0, 111.3, 109.2, 61.4, 54.3, 37.1, 31.2, 21.8, 20.8, 19.1, and 14.2. Anal. Calcd for C_17_H_21_BrO_5_: C, 53.00; H, 5.49; and Br, 20.74. Found: C, 53.07; H, 5.55; and Br, 20.81.

#### 3.4.7. Ethyl (1R*,2R*,5S*)-1,5-dimethyl-2-(4-methylbenzyl)-6,7,8-trioxabicyclo[3.2.1]octane-2-carboxylate (**2d**)

White crystals. Mp = 62–64 °С. (Lit. [63] Mp = 62–64 °С). R_f_ = 0.51 (TLC, PE:EA, 10:1). ^1^Н NMR (300.13 MHz, CDCl_3_), δ: 7.06 (d, *J* = 8.0 Hz, 2H), 6.96 (d, *J* = 8.0 Hz, 2H), 4.20 (q, *J* = 7.1 Hz, 2H), 3.31 (d, *J* = 12.9 Hz, 1H), 2.59 (d, *J* = 12.9 Hz, 1H), 2.12–1.92 (m, 2H), 2.30 (s, 3H), 1.81 (s, 3H), 1.79–1.54 (m, 2H), 1.48 (s, 3H), and 1.27 (t, *J* = 7.1 Hz, 3H). ^13^С NMR (75.48 MHz, CDCl_3_), δ: 172.6, 136.4, 132.9, 129.8, 129.0, 111.2, 109.9, 61.1, 54.3, 40.6, 32.9, 25.8, 21.2, 20.6, 18.8, and 14.2. Anal. Calcd for C_18_H_24_O_5_: C, 67.48; H, 7.55; and Cl. Found: C, 67.55; and H, 7.62.

#### 3.4.8. Ethyl (1R*,2S*,5S*)-1,5-dimethyl-2-(4-methylbenzyl)-6,7,8-trioxabicyclo[3.2.1]octane-2-carboxylate (**3d**)

White crystals. Mp = 48–50 °С. (Lit. [63] Mp = 48–50 °С). Rf = 0.47 (TLC, PE:EA, 10:1). ^1^Н NMR (300.13 MHz, CDCl_3_), δ: 7.08 (d, *J* = 8.1 Hz, 2H), 6.98 (d, *J* = 8.1 Hz, 2H), 4.15 (q, *J* = 7.1 Hz, 2H), 3.32 (d, *J* = 13.6 Hz, 1H), 3.01 (d, *J* = 13.6 Hz, 1H), 2.59 (td, *J* = 13.2, 6.5 Hz, 1H), 2.30 (s, 3H), 2.04–1.74 (m, 2H), 1.68 (s, 3H), 1.62 –1.47 (m, 1H), 1.56 (s, 3H), and 1.23 (t, *J* = 7.1 Hz, 3H). ^13^С NMR (75.48 MHz, CDCl_3_), δ: 172.7, 136.5, 134.1, 129.9, 129.1, 111.5, 109.2, 61.2, 54.5, 37.3, 31.3, 21.8, 21.1, 20.9, 19.1, and 14.2. Anal. Calcd for C_18_H_24_O_5_: C, 67.48; H, 7.55; and Cl. Found: C, 67.57; and H, 7.63.

#### 3.4.9. Ethyl (1R*,2S*,5S*)-1,5-dimethyl-2-(4-nitrobenzyl)-6,7,8-trioxabicyclo[3.2.1]octane-2-carboxylate (**2e**)

White crystals. Mp = 97–98 °С (Lit. [63] Mp = 97–98 °С). R_f_ = 0.44 (TLC, PE:EA, 5:1). ^1^Н NMR (300.13 MHz, CDCl_3_), δ: 8.10 (d, *J* = 8.7 Hz, 2H), 7.25 (d, *J* = 8.7 Hz, 2H), 4.21 (q, *J* = 7.1 Hz, 2H), 3.43 (d, *J* = 12.7 Hz, 1H), 2.74 (d, *J* = 12.7 Hz, 1H), 2.13–1.94 (m, 2H), 1.78 (s, 3H), 1.86–1.73 (m, 1H), 1.49 (s, 3H), 1.62–1.44 (m, 1H), and 1.27 (t, *J* = 7.1 Hz, 3H), ^13^С NMR (75.48 MHz, CDCl_3_), δ: 172.1, 147.2, 144.1, 130.9, 123.6, 110.9, 109.9, 61.4, 54.1, 40.8, 32.8, 25.8, 20.6, 18.7, and 14.2. Anal. Calcd for C_17_H_21_NO_7_: C, 58.11; H, 6.02; and N, 3.99. Found: C, 58.17; H, 6.08; and N, 4.05.

#### 3.4.10. Ethyl (1R*,2S*,5S*)-1,5-dimethyl-2-(4-nitrobenzyl)-6,7,8-trioxabicyclo[3.2.1]octane-2-carboxylate (**3e**)

White crystals. Mp = 143–144 °С (Lit. **[63]** Mp = 143–144 °С). R_f_ = 0.50 (TLC, PE:EA, 5:1). ^1^Н NMR (300.13 MHz, CDCl_3_), δ: 8.12 (d, *J* = 8.75 Hz, 2H), 7.31 (d, *J* = 8.7 Hz, 2H), 4.16 (q, *J* = 7.1 Hz, 2H), 3.46 (d, *J* = 13.5 Hz, 1H), 3.16 (d, *J* = 13.5 Hz, 1H), 2.75–2.59 (m, 1H), 1.94–1.78 (m, 2H), 1.66 (s, 3H), 1.57 (s, 3H), 1.47 –1.37 (m, 1H), and 1.23 (t, *J* = 7.1 Hz, 3H). ^13^С NMR (75.48 MHz, CDCl_3_), δ: 172.2, 147.2, 145.2, 131.0, 123.6, 111.0, 109.2, 61.7, 54.4, 37.5, 31.1, 21.9, 20.8, 19.0, and 14.2. Anal. Calcd for C_17_H_21_NO_5_: C, 58.11; H, 6.02; and N, 3.99. Found: C, 58.22; H, 6.11, and N, 4.09.

#### 3.4.11. Ethyl (1R*,2S*,5S*)-1,5-dimethyl-6,7,8-trioxabicyclo[3.2.1]octane-2-carboxylate (**2f**)

Colorless oil. R_f_ = 0.42 (TLC, PE:EA, 10:1). ^1^Н NMR (300.13 MHz, CDCl_3_), δ: 4.03–4.29 (m, 2H), 2.73 (d, *J* = 6.2 Hz, 1H), 2.51–2.27 (m, 1H), 2.28–2.02 (m, 1H), 2.01 –1.81 (m, 1H), 1.81–1.67 (m, 1H), 1.62 (s, 3H), 1.51 (s, 3H), and 1.27 (t, *J* = 7.1 Hz, 3H). ^13^С NMR (75.48 MHz, CDCl_3_), δ: 171.3, 110.0, 108.1, 60.9, 46.8, 31.1, 21.1, 21.0, 20.5, and 14.3. Anal. Calcd for C_10_H_16_O_5_: C, 55.55; and H, 7.46. Found: C, 55.65; and H, 7.49.

#### 3.4.12. Ethyl (1R*,2R*,5S*)-1,5-dimethyl-6,7,8-trioxabicyclo[3.2.1]octane-2-carboxylate (**3f**)

White crystals. Mp = 50–51 °С (Lit. [63] Mp = 49–50 °С). R_f_ = 0.31 (TLC, PE:EA, 10:1). ^1^Н NMR (300.13 MHz, CDCl_3_), δ: 4.16 (q, *J* = 7.1 Hz, 2H), 2.77 (dd, *J* = 12.3, 4.9 Hz, 1H), 2.57–2.37 (m, 1H), 1.98–1.70 (m, 3H), 1.57 (s, 3H), 1.51 (s, 3H), and 1.26 (t, *J* = 7.1 Hz, 3H). ^13^С NMR (75.48 MHz, CDCl_3_), δ: 171.6, 108.7, 107.7, 60.9, 49.4, 33.4, 21.3, 21.0, 20.4, and 14.3, Anal. Calcd for C_10_H_16_O_5_: C, 55.55; and H, 7.46. Found: C, 55.59; and H, 7.50.

#### 3.4.13. Ethyl (1R*,2S*,5S*)-2-ethyl-1,5-dimethyl-6,7,8-trioxabicyclo[3.2.1]octane-2-carboxylate (**2g**)

Colorless oil. R_f_ = 0.42 (TLC, PE:EA, 20:1). ^1^Н NMR (300.13 MHz, CDCl_3_), δ: 4.19 (q, *J =* 7.2 Hz, 2H), 2.33–2.04 (m, 2H), 1.99–1.74 (m, 3H), 1.67 (s, 3H), 1.47 (s, 3H), 1.53–1.38 (m, 1H), 1.28 (t, *J* = 7.2 Hz, 3H), and 0.79 (t, *J* = 7.2 Hz, 3H). ^13^С NMR (75.48 MHz, CDCl_3_), δ: 172.9, 111.3, 109.6, 60.9, 53.7, 33.0, 28.1, 25.2, 20.6, 18.7, 14.3, and 8.2. Anal. Calcd for C_12_H_20_O_5_: C, 59.00; and H, 8.25. Found: C, 59.08; and H, 8.31.

#### 3.4.14. Ethyl (1R*,2R*,5S*)-2-ethyl-1,5-dimethyl-6,7,8-trioxabicyclo[3.2.1]octane-2-carboxylate (**3g**)

Colorless oil. R_f_ = 0.36 (TLC, PE:EA, 20:1). ^1^Н NMR (300.13 MHz, CDCl_3_), δ: 4.16 (q, *J =* 7.4 Hz, 2H), 2.73–2.59 (m, 1H), 2.00–1.63 (m, 1H), 1.84–1.62 (m, 4H), 1.57 (s, 3H), 1.48 (s, 3H), 1.26 (t, *J* = 7.1 Hz, 3H), and 0.84 (t, *J* = 7.4 Hz, 3H). ^13^С NMR (75.48 MHz, CDCl_3_), δ: 172.9, 111.4, 108.9, 61.0, 53.8, 31.1, 24.5, 21.7, 20.7, 18.9, 14.3, and 9.3. Anal. Calcd for C_14_H_24_O_5_: C, 59.00; and H, 8.25. Found: C, 59.06; and H, 8.29.

#### 3.4.15. Ethyl (1R*,2S*,5S*)-2-butyl-1,5-dimethyl-6,7,8-trioxabicyclo[3.2.1]octane-2-carboxylate (**2h**)

Slightly yellow oil. R_f_ = 0.40 (TLC, PE:EA, 20:1). ^1^Н NMR (300.13 MHz, CDCl_3_), δ: 4.24–4.11 (m, 2H), 2.19–2.05 (m, 2H), 1.95–1.71 (m, 3H), 1.67 (s, 3H), 1.45 (s, 3H), 1.53 1.35 (m, 1H), 1.34 –1.18 (m, 6H), 1.06–0.80 (m, 1H), and 0.86 (t, *J* = 7.1 Hz, 3H). ^13^С NMR (75.48 MHz, CDCl_3_), δ: 173.0, 111.3, 109.6, 60.9, 53.3, 34.9, 33.0, 25.7, 26.0, 23.1, 20.6, 18.7, 14.2, and 14.0. Anal. Calcd for C_14_H_24_O_5_: C, 61.74; and H, 8.88. Found: C, 61.70; and H, 8.85.

#### 3.4.16. Ethyl (1R*,2R*,5S*)-2-butyl-1,5-dimethyl-6,7,8-trioxabicyclo[3.2.1]octane-2-carboxylate (**3h**)

Colorless oil. R_f_ = 0.33 (TLC, PE:EA, 20:1). ^1^Н NMR (300.13 MHz, CDCl_3_), δ: 4.15 (q, *J =* 7.1 Hz, 2H), 2.74–2.60 (m, 1H), 1.91–1.61 (m, 5H), 1.57 (s, 3H), 1.48 (s, 3H), 1.36–1.18 (m, 6H), 1.14–0.99 (m, 1H), and 0.89 (t, *J* = 7.1 Hz, 3H). ^13^С NMR (75.48 MHz, CDCl_3_), δ: 173.0, 111.4, 108.9, 61.0, 53.4, 31.4, 31.2, 27.2, 23.3, 22.3, 20.7, 18.9, 14.2, and 14.0. Anal. Calcd for C_14_H_24_O_5_: C, 61.74; and H, 8.88. Found: C, 61.79; and H, 8.93.

#### 3.4.17. Ethyl (1R*,2S*,5S*)-2-hexyl-1,5-dimethyl-6,7,8-trioxabicyclo[3.2.1]octane-2-carboxylate (**2i**)

Slightly yellow oil. R_f_ = 0.42 (TLC, PE:EA, 20:1). ^1^Н NMR (300.13 MHz, CDCl_3_), δ: 4.23–4.14 (m, 2H), 2.20–2.06 (m, 2H), 1.97–1.73 (m, 3H), 1.68 (s, 3H), 1.47 (s, 3H), 1.50 –1.36 (m, 1H), 1.33–1.18 (m, 10H), 1.09–0.90 (m, 1H), and 0.86 (t, *J* = 7.1 Hz, 3H). ^13^С NMR (75.48 MHz, CDCl_3_), δ: 173.1, 111.4, 109.6, 60.9, 53.4, 35.2, 33.0, 31.7, 29.7, 25.7, 23.8, 22.7, 20.7, 18.8, 14.3, and 14.1. Anal. Calcd for C_16_H_28_O_5_: C, 63.97; and H, 9.40. Found: C, 64.02; and H, 9.47.

#### 3.4.18. Ethyl (1R*,2R*,5S*)-2-hexyl-1,5-dimethyl-6,7,8-trioxabicyclo[3.2.1]octane-2-carboxylate (**3i**)

Colorless oil. R_f_ = 0.38 (TLC, PE:EA, 20:1). ^1^Н NMR (300.13 MHz, CDCl_3_), δ: 4.15 (q, *J =* 7.1 Hz, 2H), 2.77–2.58 (m, 1H), 1.90–1.61 (m, 5H), 1.58 (s, 3H), 1.49 (s, 3H), 1.36–0.97 (m, 11H), and 0.94–0.79 (m, 3H). ^13^С NMR (75.48 MHz, CDCl_3_), δ: 173.0, 111.4, 108.9, 61.0, 53.4, 31.8, 31.7, 31.2, 29.9, 25.0, 22.7, 22.4, 20.7, 18.9, 14.3, and 14.1. Anal. Calcd for C_16_H_28_O_5_: C, 63.97; and H, 9.40. Found: C, 64.05; and H, 9.48.

#### 3.4.19. Ethyl (1R*,2R*,5S*)-2-allyl-1,5-dimethyl-6,7,8-trioxabicyclo[3.2.1]octane-2-carboxylate (**2j**)

Slightly yellow oil. R_f_ = 0.29 (TLC, PE:EA, 60:1). ^1^Н NMR (300.13 MHz, CDCl_3_), δ: 5.70–5.50 (m, 1H), 5.14–5.00 (m, 2H), 4.19 (q, *J =* 7.1 Hz, 2H), 2.63 (dd, *J* = 13.2, 6.8 Hz, 1H), 2.24 –1.86 (m, 4H), 1.84 –1.73 (m, 1H), 1.69 (s, 3H), 1.48 (s, 3H), and 1.27 (t, *J* = 7.1 Hz, 3H). ^13^С NMR (75.48 MHz, CDCl_3_), δ: 172.4, 132.4, 118.9, 110.9, 109.8, 61.1, 53.1, 39.8, 32.9, 25.9, 20.7, 18.7, and 14.3. Anal. Calcd for C_13_H_20_O_5_: C, 60.92; and H, 7.87. Found: C, 60.96; and H, 7.92.

#### 3.4.20. Ethyl (1R*,2S*,5S*)-2-allyl-1,5-dimethyl-6,7,8-trioxabicyclo[3.2.1]octane-2-carboxylate (**3j**)

Slightly yellow oil. R_f_ = 0.23 (TLC, PE:EA, 20:1). ^1^Н NMR (300.13 MHz, CDCl_3_), δ: 5.72–5.53 (m, 1H), 5.13–5.03 (m, 2H), 4.16 (q, *J =* 7.1 Hz, 2H), 2.78 –2.56 (m, 2H), 2.48 (dd, *J* = 13.9, 8.7 Hz, 1H), 1.83 –1.60 (m, 3H), 1.57 (s, 3H), 1.50 (s, 3H), and 1.26 (t, *J* = 7.1 Hz, 3H). ^13^С NMR (75.48 MHz, CDCl_3_), δ: 172.6, 133.7, 118.7, 110.9, 109.0, 61.2, 52.8, 36.4, 30.8, 22.7, 20.8, 18.8, and 14.3. Anal. Calcd for C_13_H_20_O_5_: C, 60.92; and H, 7.87. Found: C, 60.97; and H, 7.91.

#### 3.4.21. Ethyl (1R*,2S*,5S*)-2-(2-cyanoethyl)-1,5-dimethyl-6,7,8-trioxabicyclo[3.2.1]octane-2-carboxylate (**2k**)

White crystals. Mp = 83–84 °С (Lit. [63] Mp = 83–84 °С). R_f_ = 0.60 (TLC, PE:EA, 5:1). ^1^Н NMR (300.13 MHz, CDCl_3_), δ: 4.25 (q, J = 7.1 Hz, 2H), 2.40–1.77 (m, 8H), 1.65 (s, 3H), 1.49 (s, 3H), and 1.31 (t, J = 7.1 Hz, 3H). ^13^С NMR (75.48 MHz, CDCl_3_), δ: 171.6, 118.9, 110.4, 109.7, 61.8, 52.2, 32.6, 30.8, 25.1, 20.6, 18.6, 14.2, and 12.4. Anal. Calcd for C_13_H_19_NO_5_: C, 57.98; H, 7.11; and N, 5.20. Found: C, 58.05; H, 7.16; and N, 5.27.

#### 3.4.22. Ethyl (1R*,2R*,5S*)-2-(2-cyanoethyl)-1,5-dimethyl-6,7,8-trioxabicyclo[3.2.1]octane-2-carboxylate (**3k**)

Colorless oil. R_f_ = 0.51 (TLC, PE:EA, 5:1). ^1^Н NMR (300.13 MHz, CDCl_3_), δ: 4.22 (q, *J* = 7.1 Hz, 2H), 2.88–2.75 (m, 1H), 2.45–2.16 (m, 4H), 1.94–1.78 (m, 2H), 1.73–1.61 (m, 1H), 1.53 (s, 3H), 1.52 (s, 3H), and 1.29 (t, *J* = 7.1 Hz, 3H). ^13^С NMR (75.48 MHz, CDCl_3_), δ: 171.8, 119.4, 110.3, 109.0, 61.8, 52.7, 30.9, 27.1, 22.1, 20.6, 18.6, 14.2, and 13.4. Anal. Calcd for C_13_H_19_NO_5_: C, 57.98; H, 7.11; and N, 5.20. Found: C, 58.04; H, 7.13; and N, 5.26.

### 3.5. Synthesis of (2-(4-chlorobenzyl)-1,5-dimethyl-6,7,8-trioxabicyclo[3.2.1]octan-2-yl)methanol *(**4**)*

LiAlH_4_ (0.152 g, 4.0 mmol) was added to the solution of ozonides **2a** and **3a** (0.341 g, 1.0 mmol) in dry THF (10 mL), with stirring in the argon atmosphere at −22 °С. The reaction mixture was stirred at −22 °С for 72 h. Then, 5 mL of 5M NaOH solution and 15 mL of water were added at −22 °С. The reaction mixture was warmed to r.t. Then, 30 mL of CHCl_3_ was added. The organic layer was separated. Peroxide **4** was extracted with CHCl_3_ (3 × 15 mL) from the aqueous layer. The combined organic layers were washed with 5 mL of water, dried over MgSO_4_, and filtered. The solvent was removed and pure product **4** was obtained. Ozonide **4**, 60.0 mg, 0.2 mmol, and yield 20%.

#### (1R*,2S*,5S*)-2-(4-chlorobenzyl)-1,5-dimethyl-6,7,8-trioxabicyclo[3.2.1]octan-2-yl)methanol (**4**)

Colorless oil. R_f_ = 0.35 (TLC, PE:EA, 5:1). ^1^Н NMR (300.13 MHz, CDCl_3_), δ: 7.26 (d, *J* = 8.4 Hz, 2H), 7.18 (d, *J* = 8.4 Hz, 2H), 3.92 (dd, *J* = 11.0 Hz, 4.5 Hz, 1H), 3.52 (d, *J* = 11.0 Hz, 1H), 2.97 (d, *J* = 12.9 Hz, 1H), 2.55 (d, *J* = 12.9 Hz, 1H), 2.19–2.05 (m, 1H), 1.80–1.70 (m, 3H), 1.68 (s, 3H), and 1.51 (s, 3H). ^13^С NMR (75.48 MHz, CDCl_3_), δ: 135.3, 132.4, 132.0, 128.2, 112.9, 109.1, 64.4, 44.6, 37.5, 32.2, 26.4, 20.8, and 18.7. HRMS (ESI-TOF): m/z [M + H]^+^: calculated for [C_15_H_20_ClO_4_]+: 299.1046; found: 299.1045. Anal. Calcd for C_15_H_19_ClO_4_: C, 60.30; H, 6.41; and Cl, 11.87. Found: C, 60.41; H, 6.52; and Cl, 11.96.

### 3.6. Maintenance of the Parasites at the Swiss TPH

The intermediate host, *Biomphalaria glabrata* snails infected with *S. mansoni*, were kept in water tanks under a natural temperature and humidity level. Three-week-old female NMRI mice were purchased from Charles River (Sulzfeld, Germany). After one week of acclimatization at the Swiss TPH animal facility, they were infected subcutaneously with 100 freshly harvested *S. mansoni* cercariae. They were then maintained for 7 weeks at 22 °C and 50% humidity, with an artificial 12 h day/night cycle and free access to rodent diet and water. All the procedures were performed according to the Swiss federal law and the cantonal regulations on animal experimentation (licence n°2070).

### 3.7. In Vitro Compound Screening on S. mansoni NTS and Adult S. mansoni

The *S. mansoni* cercariae were transformed into NTS according to standard procedures [68]. The resulting NTS suspension was diluted to a concentration of 100 NTS per 50 μL with the use of Medium 199 (Invitrogen, Carlsbad, CA, USA). The Medium 199 was supplemented with 5% iFCS (heat-inactivated fetal calf serum), 100 U/mL of penicillin, and 100 μg/mL of streptomycin (Invitrogen, Carlsbad, CA, USA). The NTS suspension was incubated at 37 °C (5% CO_2_ in ambient air) to ensure its completed conversion into schistosomula (minimum of 12−24 h). After this, drug solutions were prepared in 96-flat bottom well-plates (BD Falcon, Corning, NY, USA) at concentrations of 33 and 10 µM with the use of the supplemented (iFCS and antibiotics) Medium 199. The NTS suspension was added to each well. The plates were incubated at 37 °C (5% CO_2_ in ambient air) for 72h. NTS incubated in the presence of a blank medium containing the highest concentration of DMSO used served as a control. The NTS were evaluated using microscopy (Carl Zeiss, Germany, magnification 80×).

Adult flukes were collected from the hepatic portal and mesenteric veins of the infected NMRI mice (7−8 weeks post-infection), as described recently [68]. The schistosomes were placed in RPMI 1640 culture medium. The RPMI 1640 was supplemented with 5% iFCS, 100 U/mL of penicillin, and 100 μg/mL streptomycin. Then, the schistosomes in the medium were incubated at 37 °C (5% CO_2_ in ambient air) until their use. At least three schistosomes of both sexes were added to each well of 24-flat bottom well-plates (BD Falcon, USA), containing 2 mL of medium and 10 µM of the test drug. Schistosomes incubated in the presence of a blank medium containing the highest concentration of DMSO used served as a control. The schistosomes were incubated for 72 h and evaluated using microscopy, as summarized recently [68].

## 4. Conclusions

In summary, we have disclosed that SnCl_4_ is an efficient catalyst for the ozone-free synthesis of bridged ozonides from 1,5-diketones and hydrogen peroxide in moderate to high yields. Thus, we discovered a new nature of SnCl_4_ and new directions for its use in peroxide chemistry. We have also demonstrated that SnCl_4_ used as a catalyst along with a varying solvent affects the ratio of target ozonides. The ozonide cycle is moderately stable to LiAlH_4_, which makes it possible to carry out the reduction of the ester group located near the peroxide cycle. Such a transformation can open up additional features for the synthesis of novel hybrid molecules. Ozonides **2d**, **2e,** and **3c** exhibited a very high activity against newly transformed schistosomula. The results obtained can be useful for the development of peroxide-based compounds against schistosomiasis.

## Figures and Tables

**Table 1:** Peroxidation of diketone **1a** ^a^.

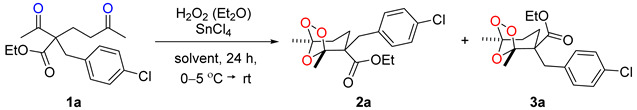					
Entry	Equiv. of H_2_O_2_ vs. 1a	Equiv. of SnCl_4_ vs. 1a	Solvent	Isolated yield of 2a + 3a, %	Ratio of 2a:3a
1	1.5	SnCl_4_ (1.0)	THF	21	1.4:1.0
2	1.5	SnCl_4_ (3.0)	THF	64	1.2:1.0
3	3.0	SnCl_4_ (3.0)	THF	70	1:1
4	1.5	SnCl_4_ (5.0)	THF	83	1:1
5	3.0	SnCl_4_ (5.0)	THF	71	1:1
6	1.5	SnCl_4_ (5.0)	1,4-Dioxane	81	2.6:1.0
7	1.5	SnCl_4_ (5.0)	Et_2_O	79	8.6:1.0
8	1.5	SnCl_4_ (5.0)	СН_3_СN	-	-
9	1.5	SnCl_4_ (5.0)	СН_2_Сl_2_	-	-

^a^ A 5.1 M ethereal solution of H_2_O_2_ (1.5–3.0 mol Н_2_О_2_/1.0 mol of 1,5-diketone **1a**) and SnCl_4_ (1.0–5.0 mol SnCl_4_/1.0 mol of **1a**) were added to a solution of **1a** (0.30 g, 0.9 mmol) in a solvent (5 mL) with stirring at 0–5 °С. The reaction mixture was warmed to 20–25 °C and stirred for 24 h.

**Table 2:** In vitro activity of peroxides (bridged ozonides) on NTS and adult *S. mansoni*.

Compound	NTS		Adult
	(Effect %) at 33.3 µM	(Effect %) at 10 µM	(Effect %) at 10 µM
2b	100 ± 0	74 ± 5	23 ± 4
2c	100 ± 0	67 ± 20	70 ± 2
2d	100 ± 0	100 ± 0	82 ± 0
2e	100 ± 0	100 ± 0	14 ± 4
2f	93 ± 11	30 ± 5	ND
2g	36 ± 22	39 ± 5	ND
2h	77 ± 38	17 ± 5	ND
2i	100 ± 0	87 ± 5	55 ± 3
2j	96 ± 5	23 ± 9	ND
2k	90 ± 0	23 ± 0	ND
3b	22 ± 0	12 ± 0	ND
3c	100 ± 0	100 ± 0	76 ± 0
3d	74 ± 11	22 ± 0	ND
3e	22 ± 5	12 ± 0	ND
3f	61 ± 6	11 ± 6	ND
3g	73 ± 6	15 ± 6	ND
3h	100 ± 0	42 ± 11	ND
3j	100 ± 0	88 ± 0	23 ± 4
3k	12 ± 0	3.2 ± 0	ND
Artesunate	-	63 ±0	not active
Praziquantel	-	77 ± 0	100 ± 0

ND: not done, because the effect at 10 µM on NTS was <50%.

## Data Availability

The data presented in this study are available in insert article or Supplementary Materials here.

## Supplementary Materials

The following supporting information can be downloaded at: https://www.mdpi.com/article/10.3390/molecules28134913/s1, NMR and HRMS spectra.

## Sample Availability

Samples of the compounds **2**–**4** are available from the authors.
