# Supplementary material for: Bridged 1,2,4-Trioxolanes: SnCl4—Catalyzed Synthesis and an In Vitro Study against S. mansoni
Source: Molecules. 2023 Jun 22;28(13):4913. doi: 10.3390/molecules28134913 (PMC10343456; doi:10.3390/molecules28134913)
Supplement: Supplementary file 1 [file molecules-28-04913-s001.zip › molecules-2265067-supplementary.pdf]

Supporting information

Bridged 1,2,4-Trioxolanes: $SnCl_4$ - Catalyzed Synthesis and an In Vitro Study against *S. mansoni*

Peter S. Radulov[1], Ivan A. Yaremenko[1], Jennifer Keiser[2,3] and Alexander O. Terent'ev[1]*

[1]N. D. Zelinsky Institute of Organic Chemistry, Russian Academy of Sciences, 47 Leninsky Prospekt, 119991 Moscow, Russia

[2] Department of Medical Parasitology and Infection Biology, Swiss Tropical and Public Health Institute, CH-4123 Allschwil Switzerland

[3] University of Basel, CH-4003 Basel, Switzerland

* Correspondence: alterex@yandex.ru; Tel.: +7-916-385-4080

NMR spectra of peroxides 2a-k and 3a-k

^{1}H NMR (300.13 MHz, CDCl3). Ethyl (1*R**,2*R**,5*S**)-2-(4-chlorobenzyl)-1,5-dimethyl-6,7,8-trioxabicyclo[3.2.1]octane-2-carboxylate, 2a

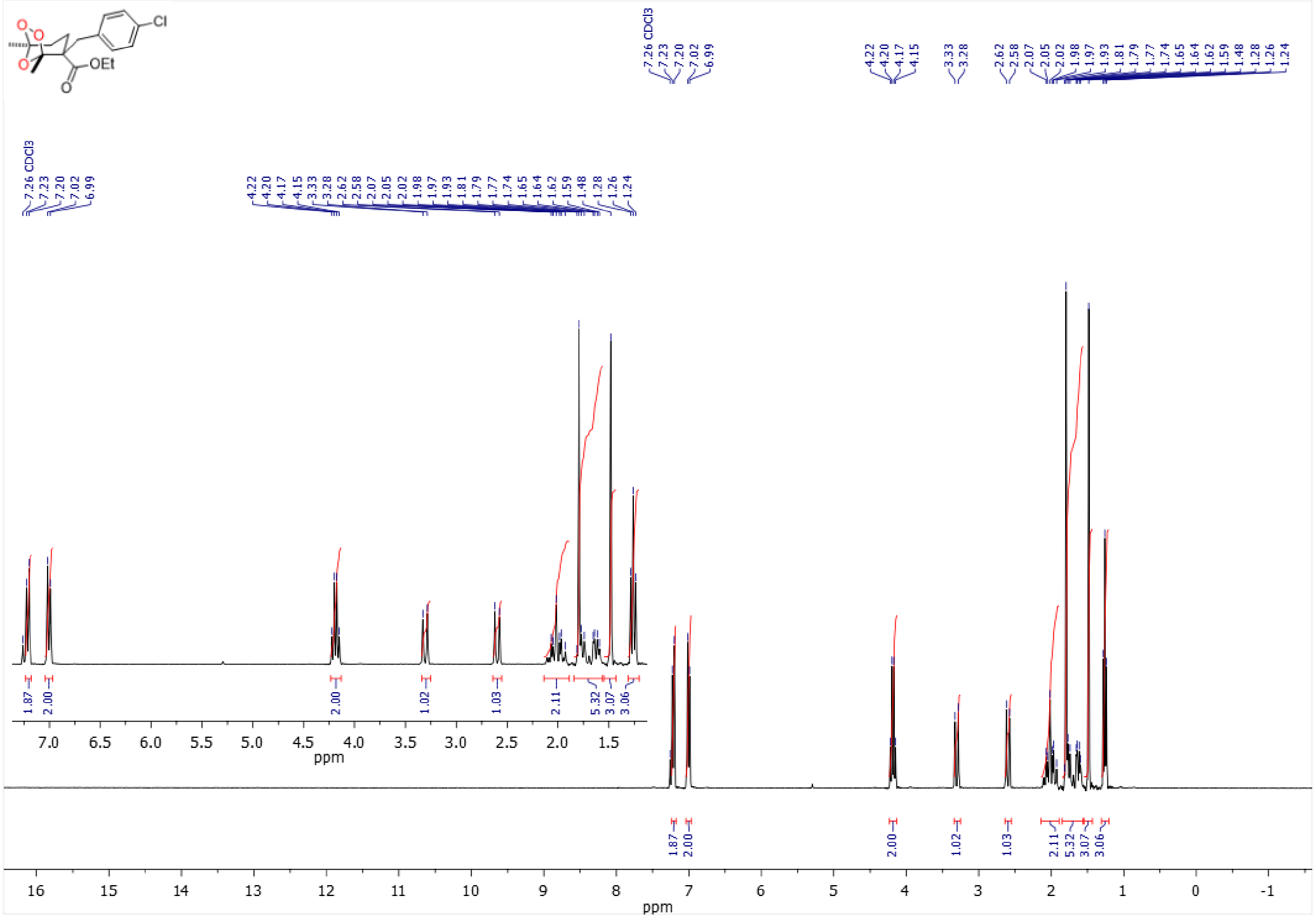

^{13}C NMR (75.48 MHz, CDCl$_3$). Ethyl (1*R,2*R**,5*S**)-2-(4-chlorobenzyl)-1,5-dimethyl-6,7,8-trioxabicyclo[3.2.1]octane-2-carboxylate, 2a**

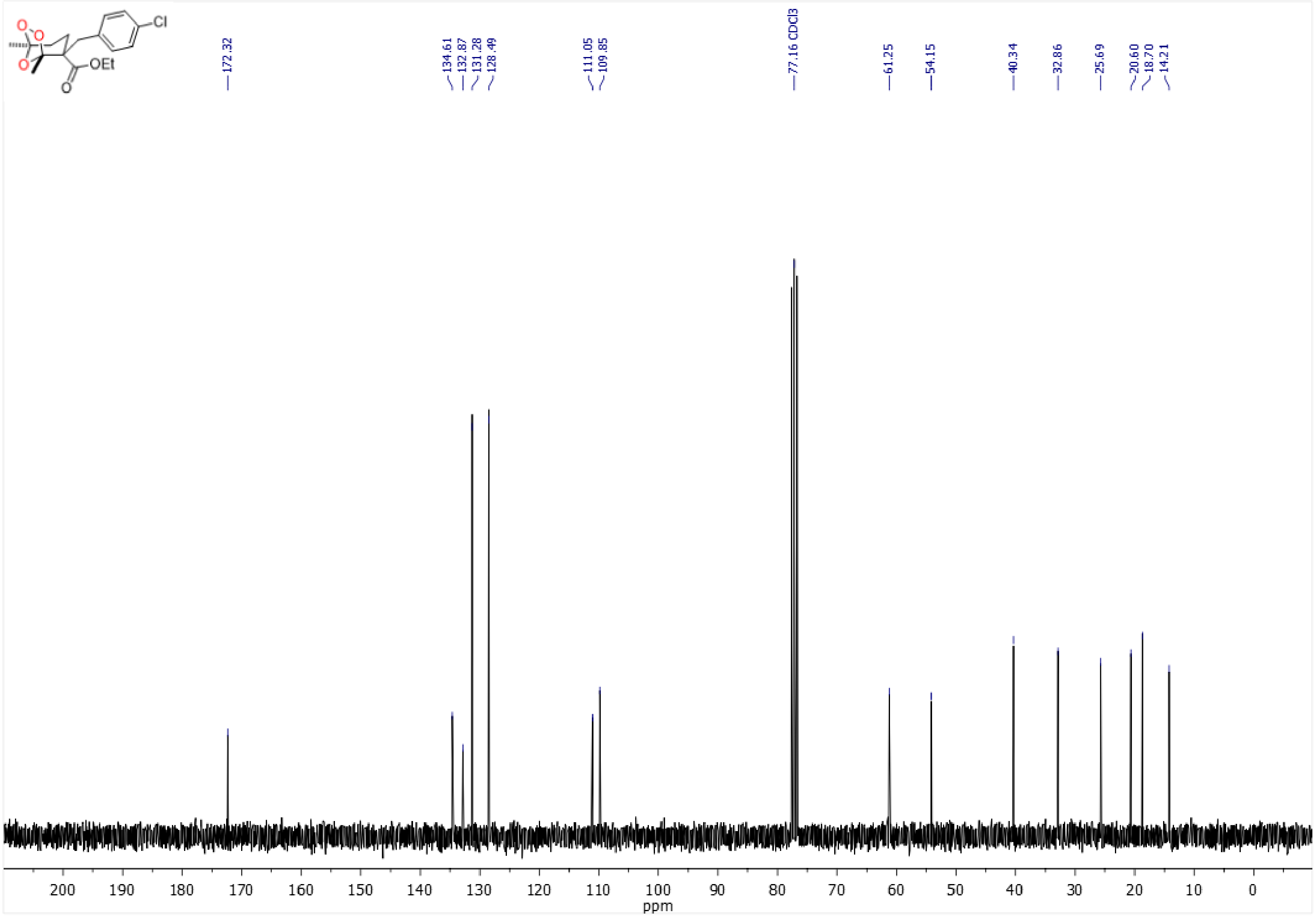

^{1}H NMR (300.13 MHz, $CDCl_3$). Ethyl (1*R**,2*S**,5*S**)-2-(4-chlorobenzyl)-1,5-dimethyl-6,7,8-trioxabicyclo[3.2.1]octane-2-carboxylate, 3a

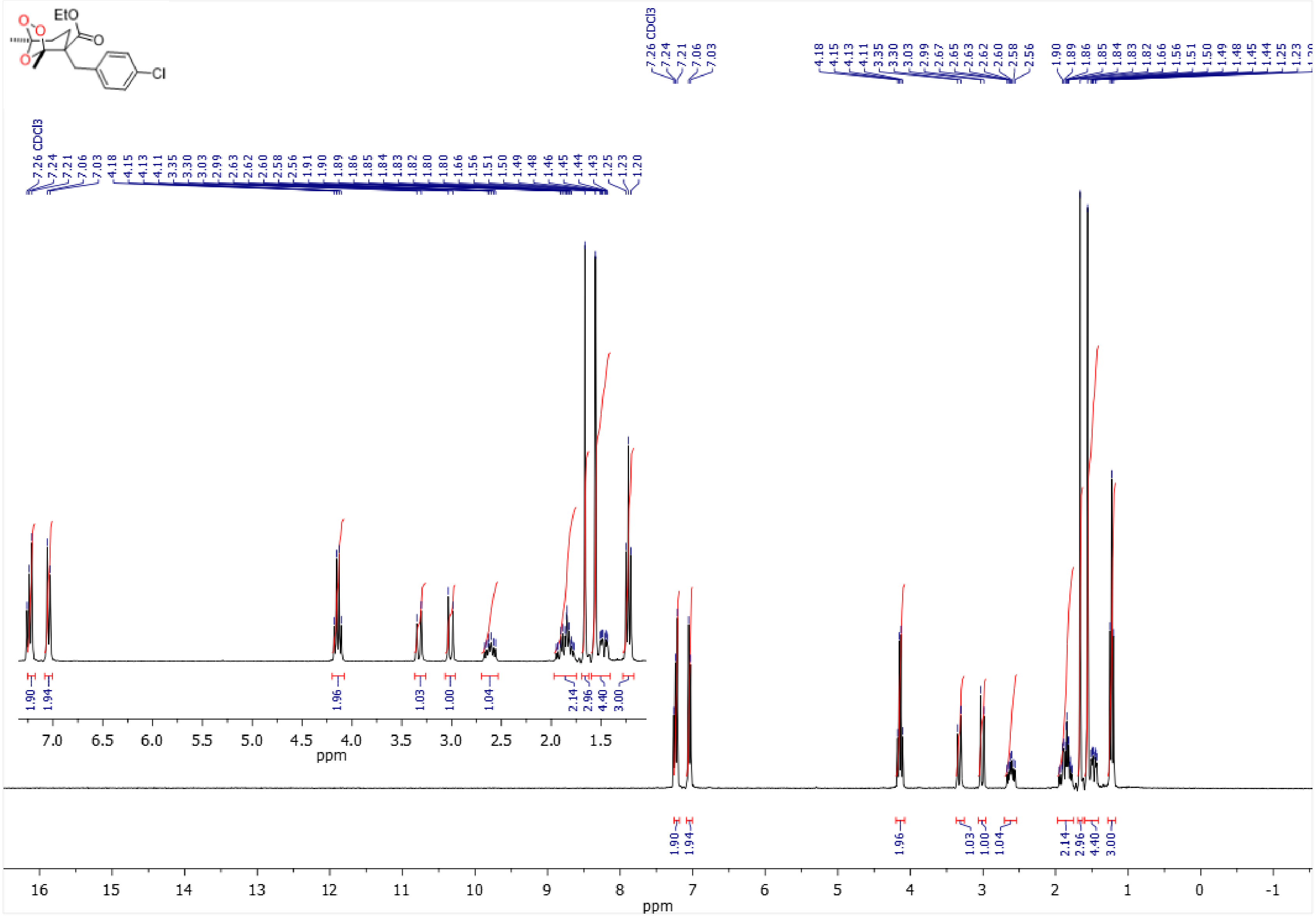

^{13}C NMR (75.48 MHz, $CDCl_3$). Ethyl (1*R**,2*S**,5*S**)-2-(4-chlorobenzyl)-1,5-dimethyl-6,7,8-trioxabicyclo[3.2.1]octane-2-carboxylate, 3a

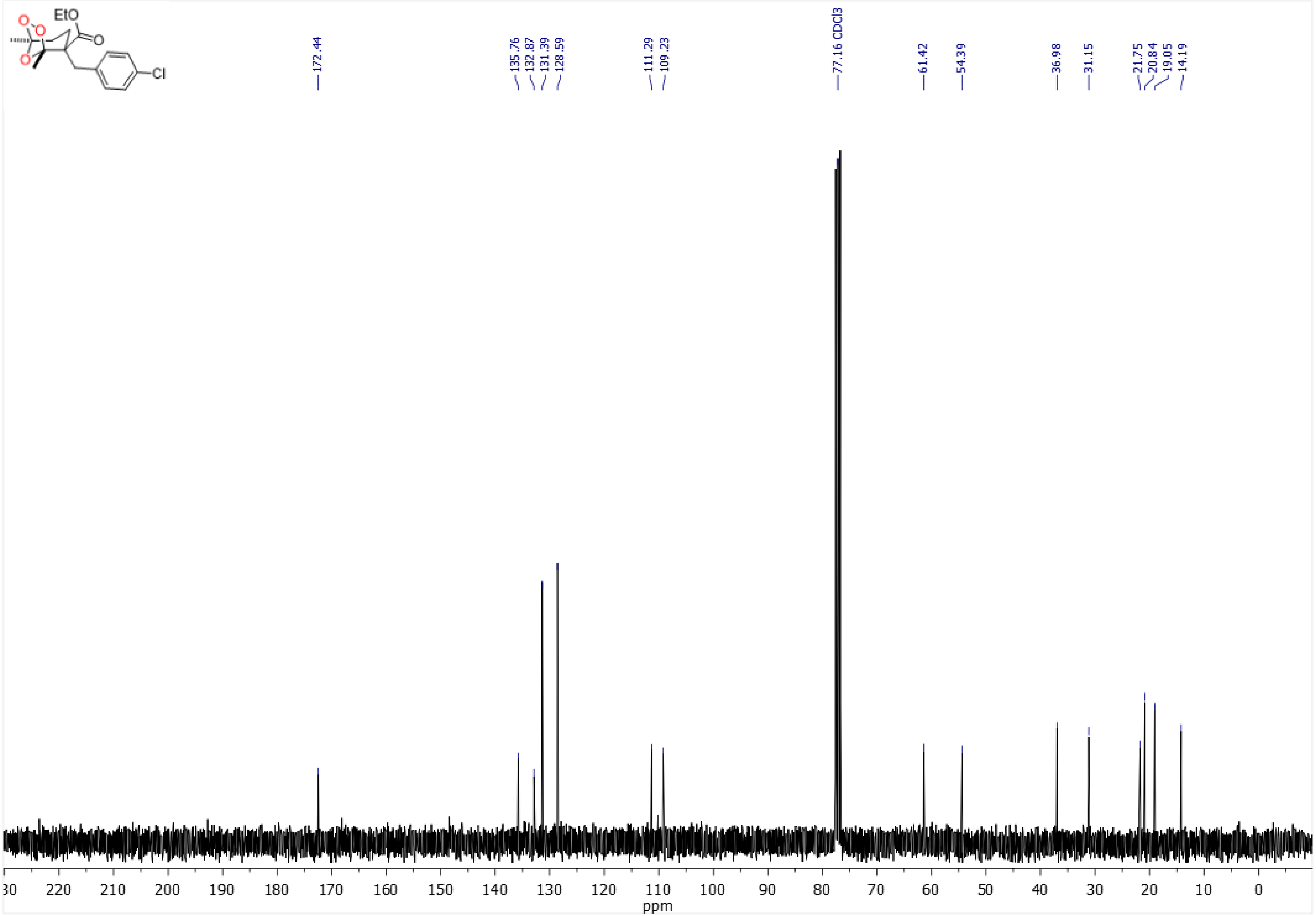

^{1}H NMR (300.13 MHz, $CDCl_3$). Ethyl (1*R**,2*R**,5*S**)-2-benzyl-1,5-dimethyl-6,7,8-trioxabicyclo[3.2.1]octane-2-carboxylate, 2b

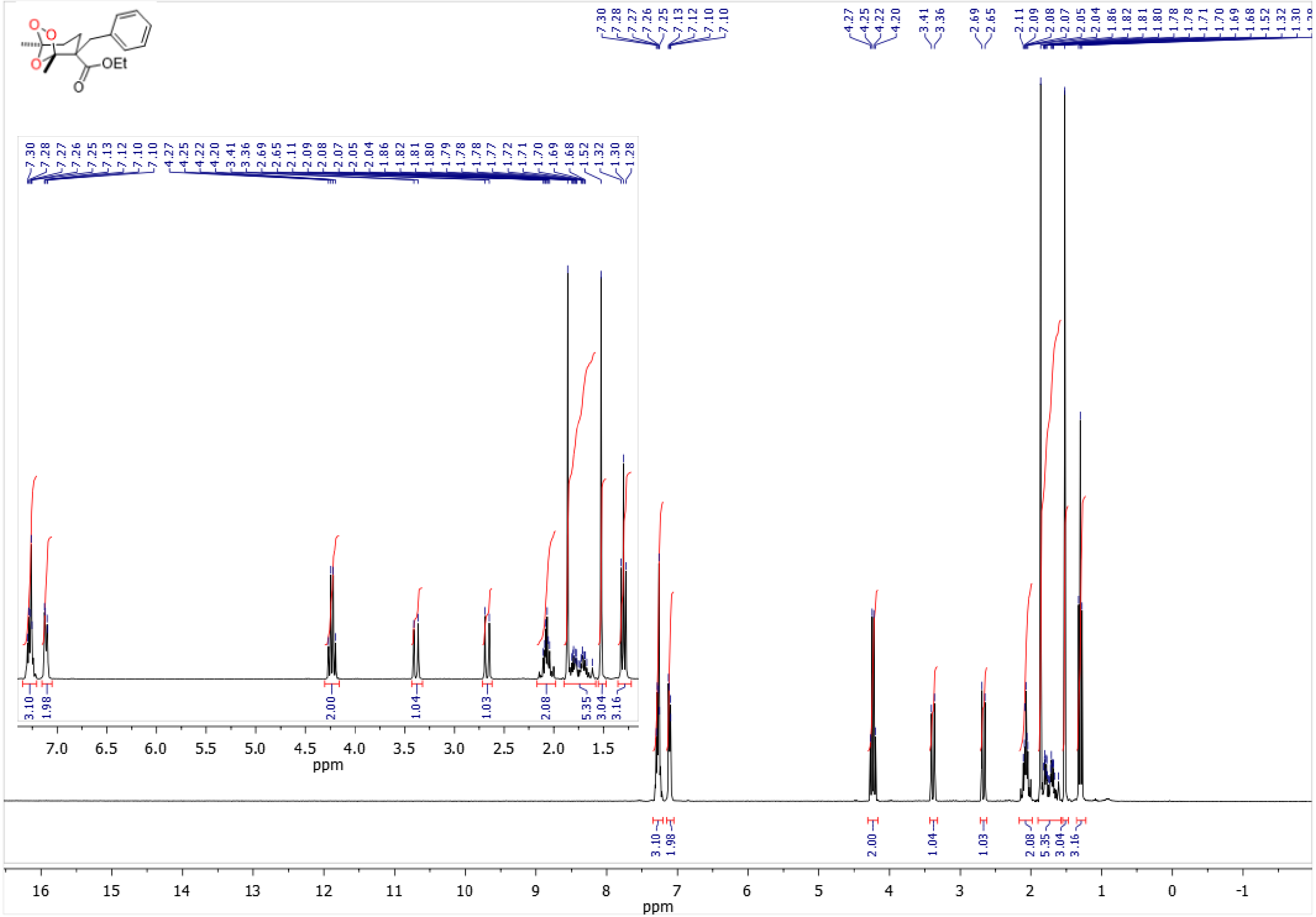

^{13}C NMR (75.48 MHz, $CDCl_3$). Ethyl (1*R**,2*R**,5*S**)-2-benzyl-1,5-dimethyl-6,7,8-trioxabicyclo[3.2.1]octane-2-carboxylate, 2b

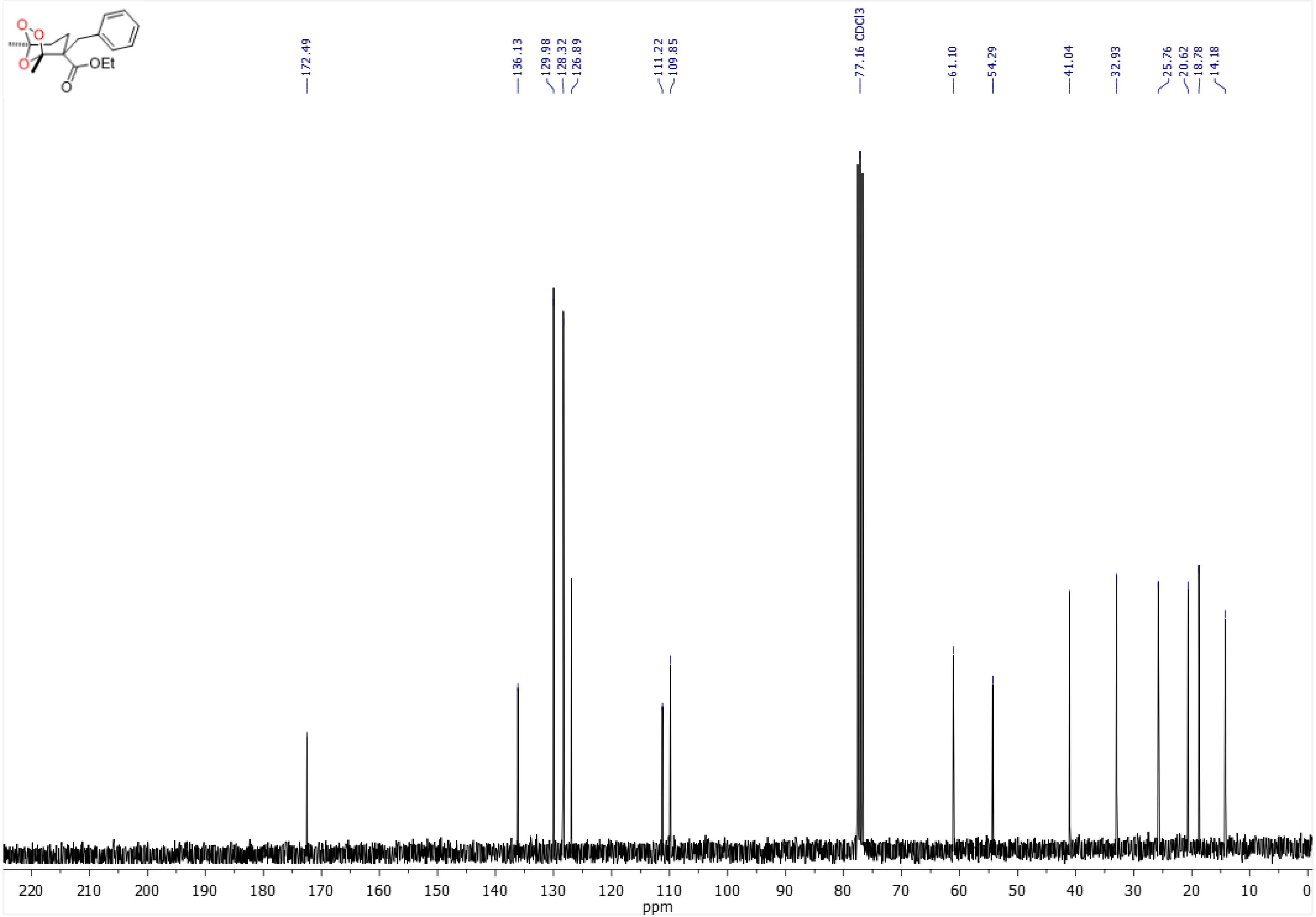

^{1}H NMR (300.13 MHz, $CDCl_3$). Ethyl (1*R**,2*S**,5*S**)-2-benzyl-1,5-dimethyl-6,7,8-trioxabicyclo[3.2.1]octane-2-carboxylate, 3b

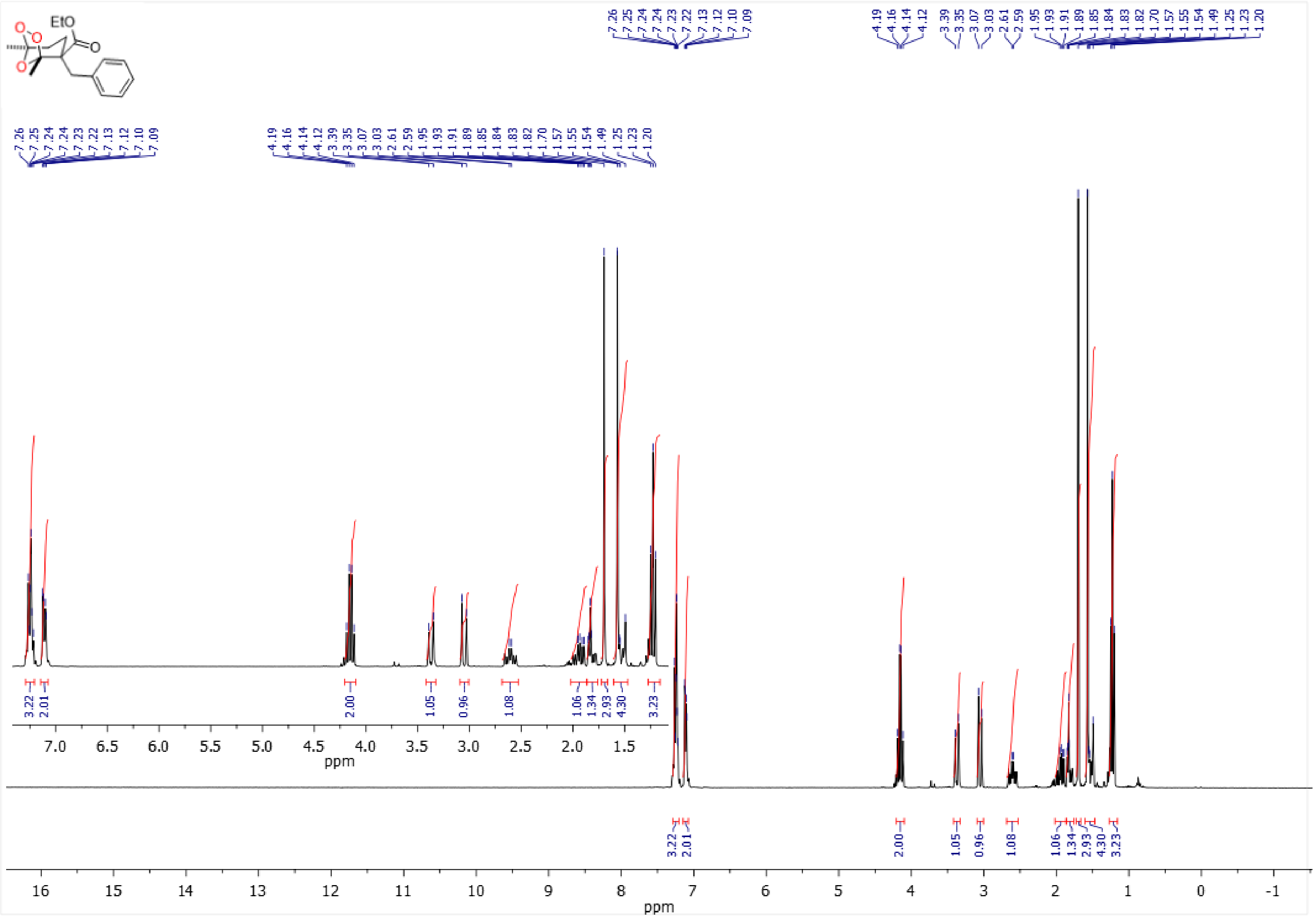

^{13}C NMR (75.48 MHz, CDCl$_3$). Ethyl (1*R**,2*S**,5*S**)-2-benzyl-1,5-dimethyl-6,7,8-trioxabicyclo[3.2.1]octane-2-carboxylate, 3b

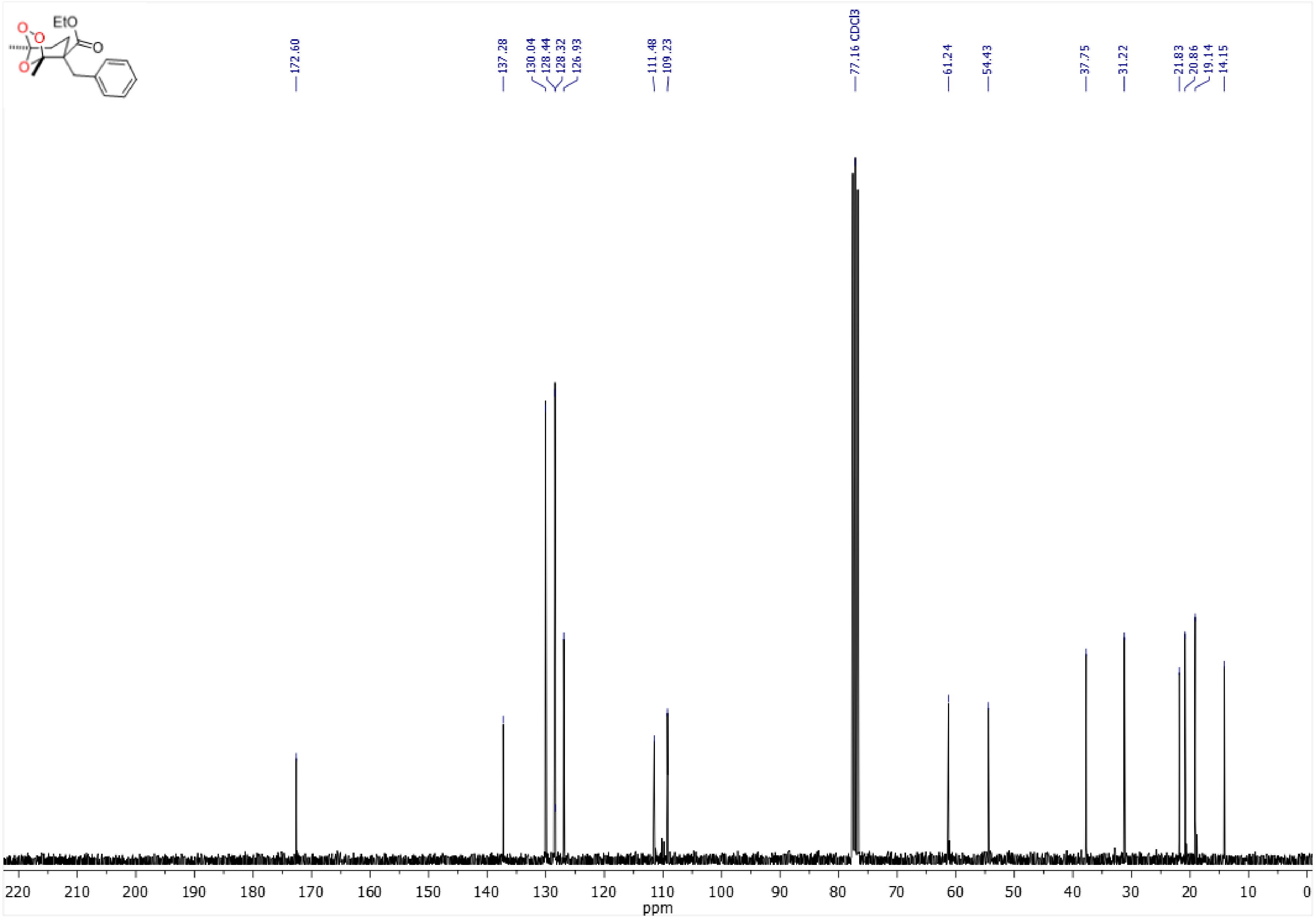

^{1}H NMR (300.13 MHz, $CDCl_3$). Ethyl (1*R**,2*R**,5*S**)-2-(4-bromobenzyl)-1,5-dimethyl-6,7,8-trioxabicyclo[3.2.1]octane-2-carboxylate, 2c

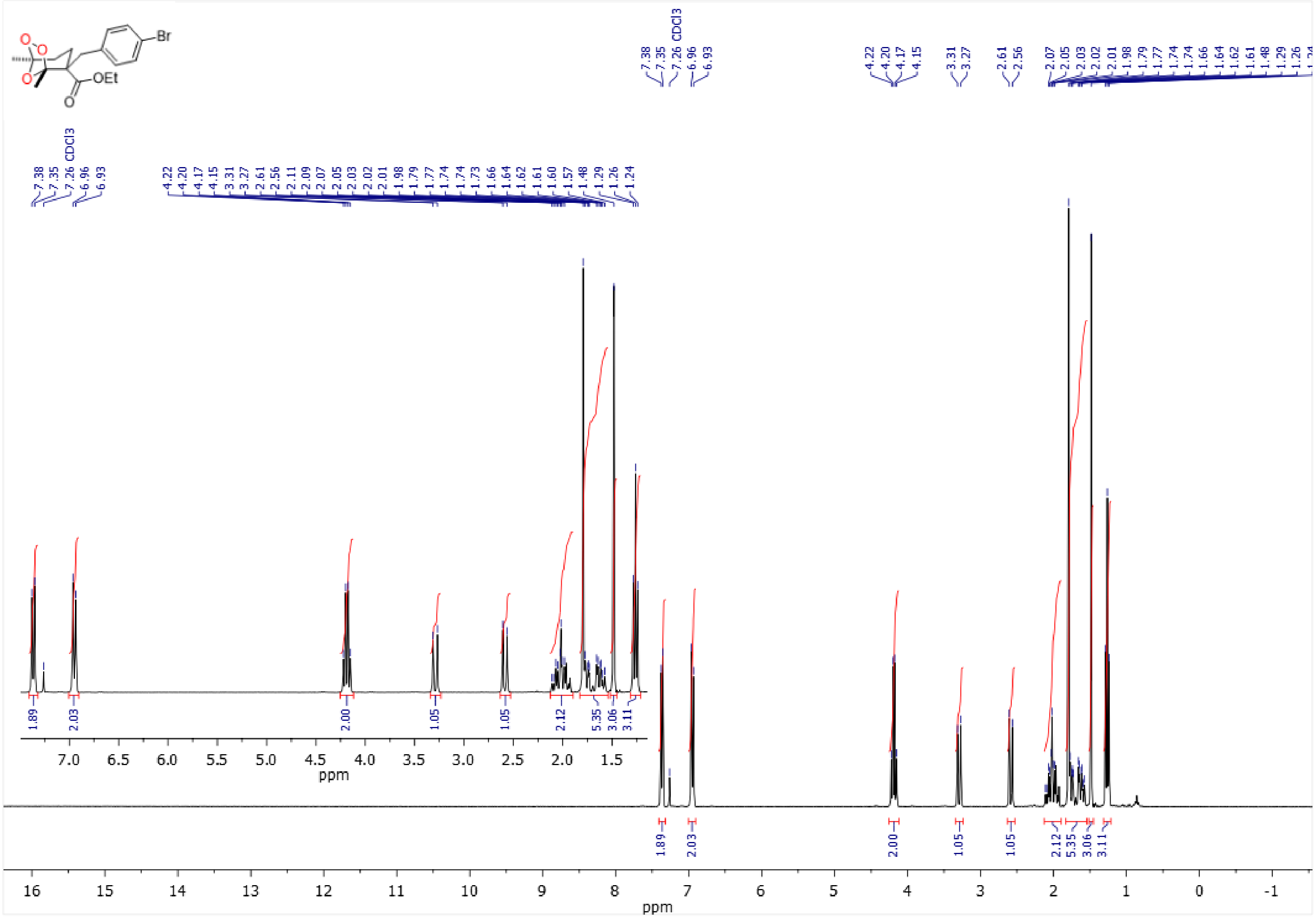

^{13}C NMR (75.48 MHz, CDCl₃). Ethyl (1*R**,2*R**,5*S**)-2-(4-bromobenzyl)-1,5-dimethyl-6,7,8-trioxabicyclo[3.2.1]octane-2-carboxylate, 2c

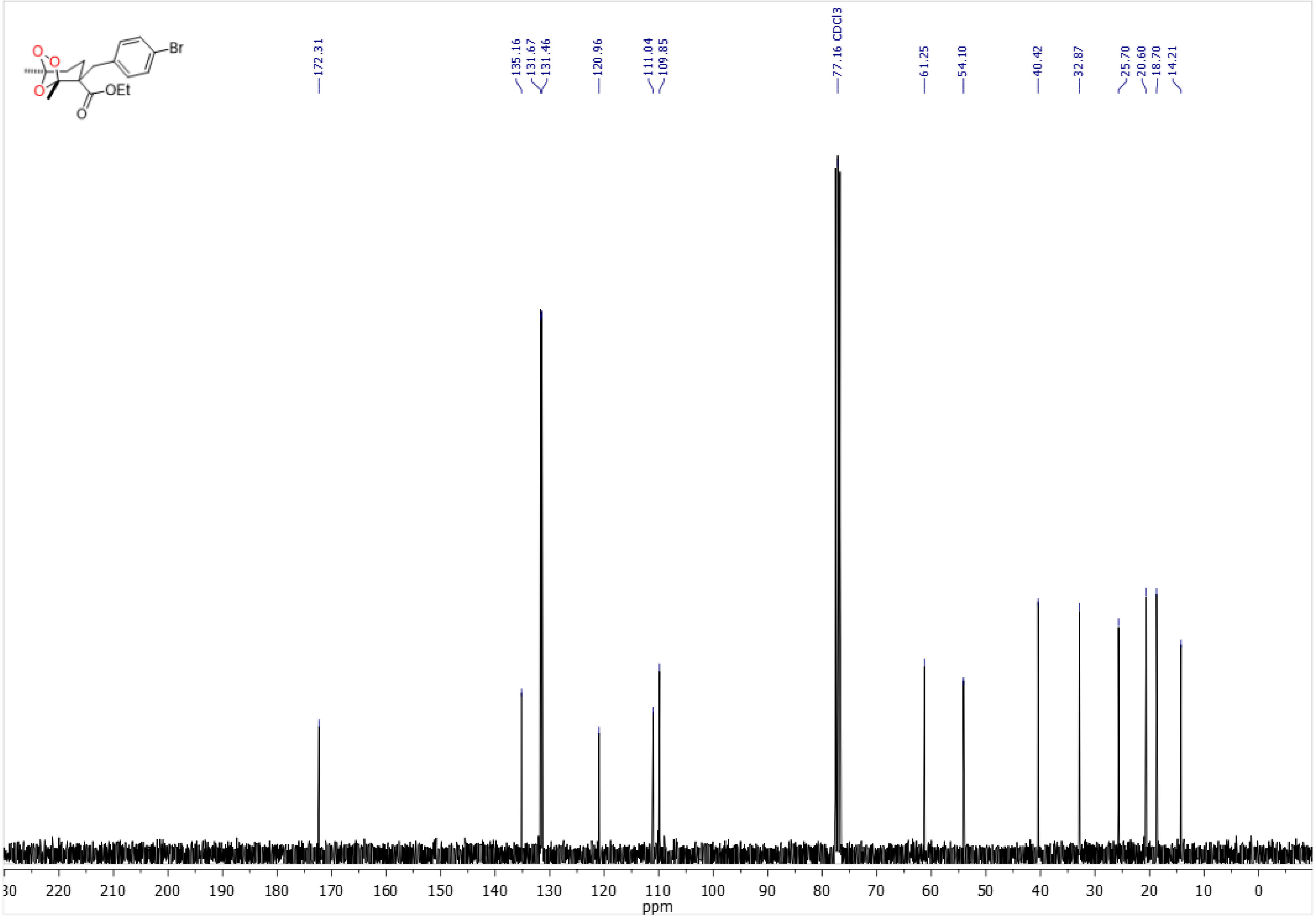

^{1}H NMR (300.13 MHz, $CDCl_3$). Ethyl (1*R,2*S**,5*S**)-2-(4-bromobenzyl)-1,5-dimethyl-6,7,8-trioxabicyclo[3.2.1]octane-2-carboxylate, 3c**

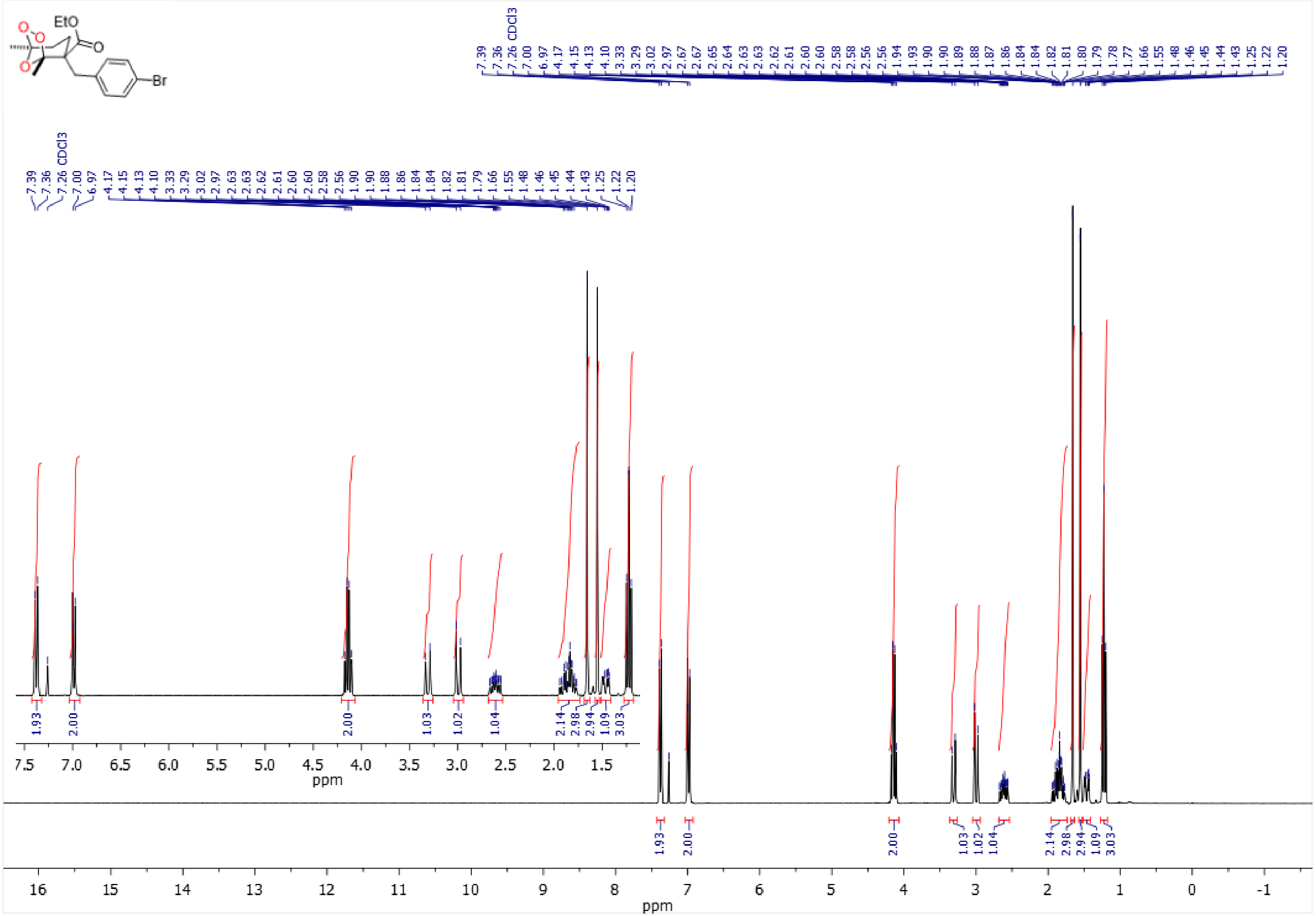

^{13}C NMR (75.48 MHz, $CDCl_3$). Ethyl (1*R**,2*S**,5*S**)-2-(4-bromobenzyl)-1,5-dimethyl-6,7,8-trioxabicyclo[3.2.1]octane-2-carboxylate, 3c

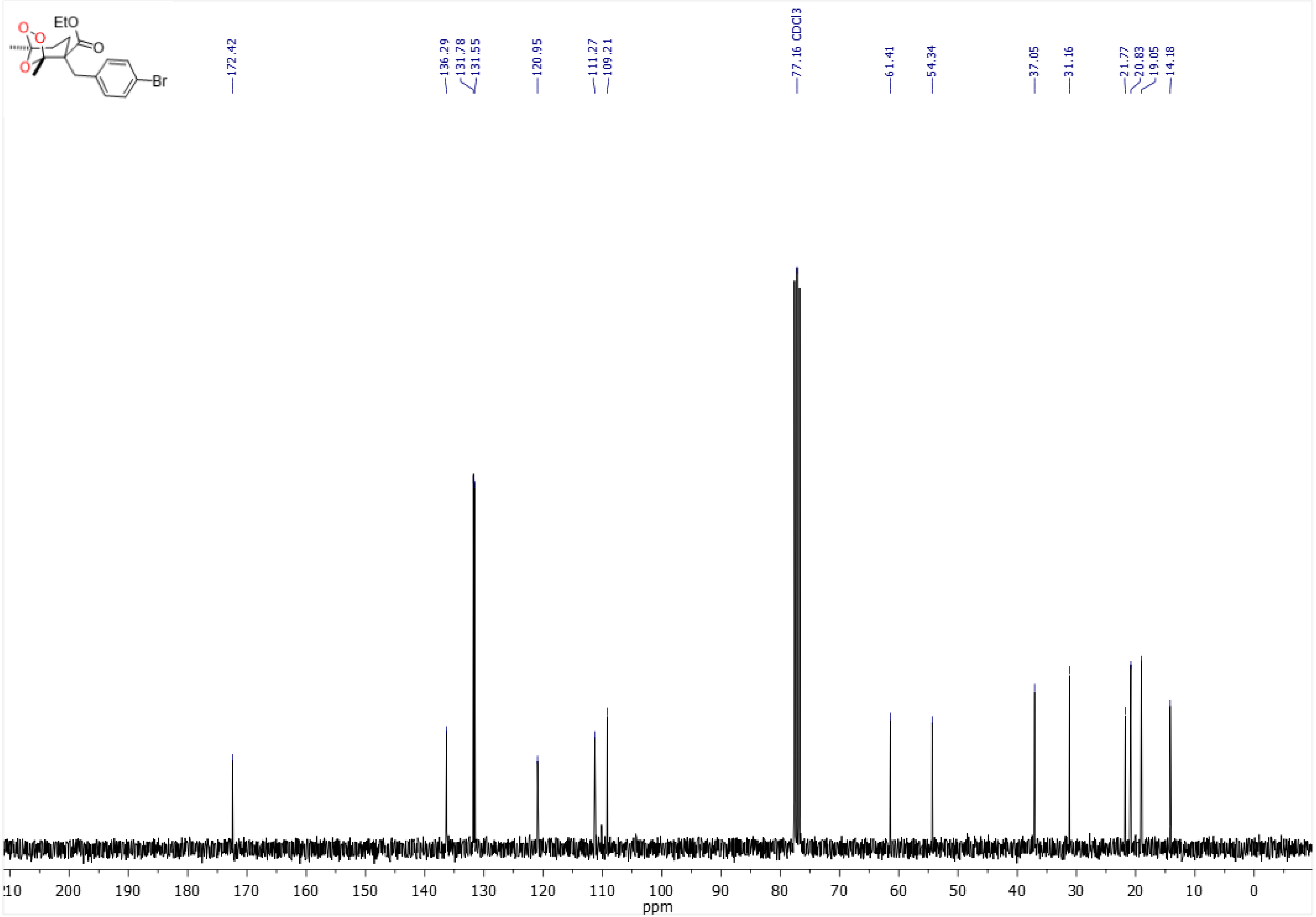

^{1}H NMR (300.13 MHz, $CDCl_3$). Ethyl (1R*,2R*,5S*)-1,5-dimethyl-2-(4-methylbenzyl)-6,7,8-trioxabicyclo[3.2.1]octane-2-carboxylate, 2d

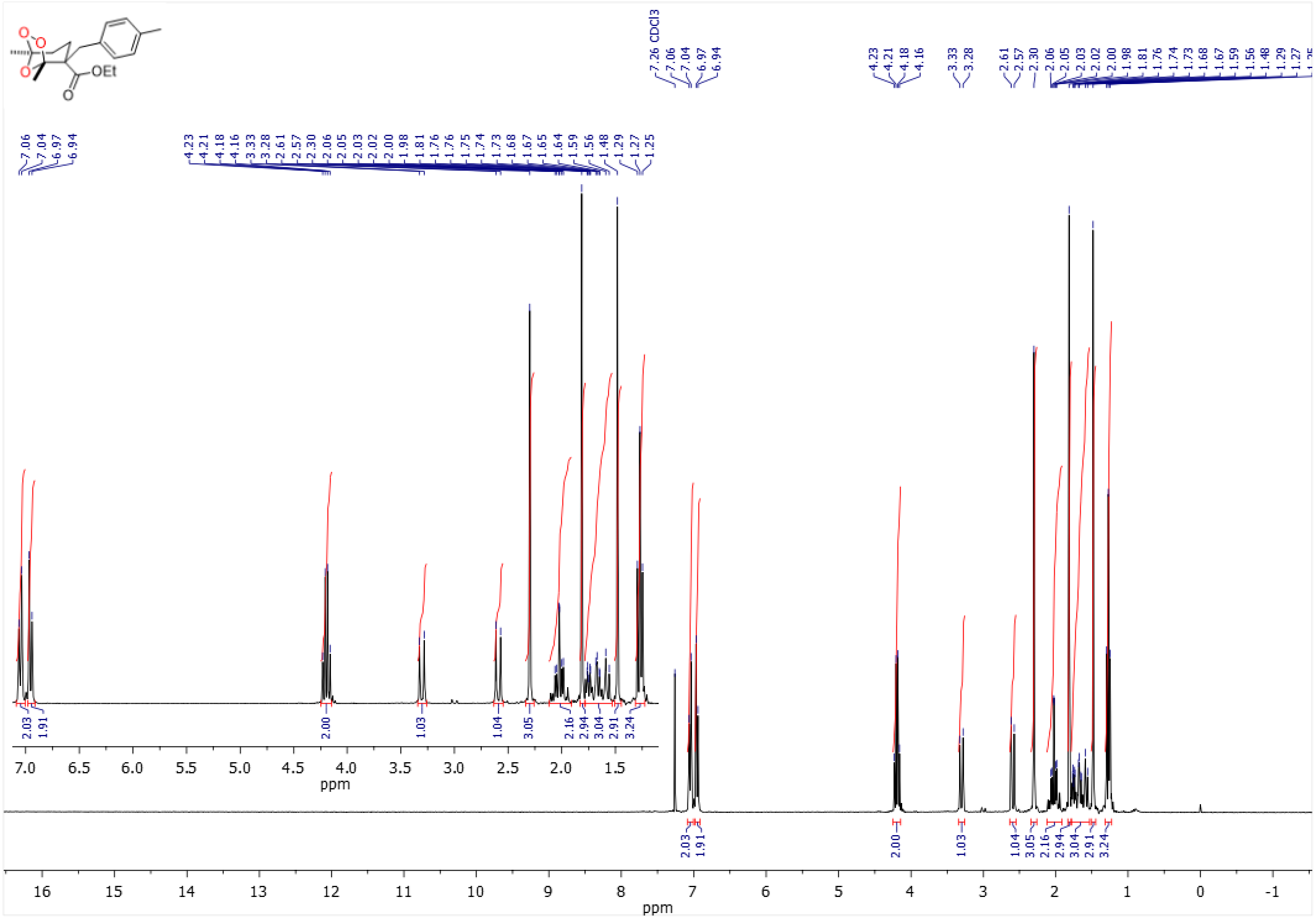

^{13}C NMR (75.48 MHz, CDCl3). Ethyl (1R*,2R*,5S*)-1,5-dimethyl-2-(4-methylbenzyl)-6,7,8-trioxabicyclo[3.2.1]octane-2-carboxylate, 2d

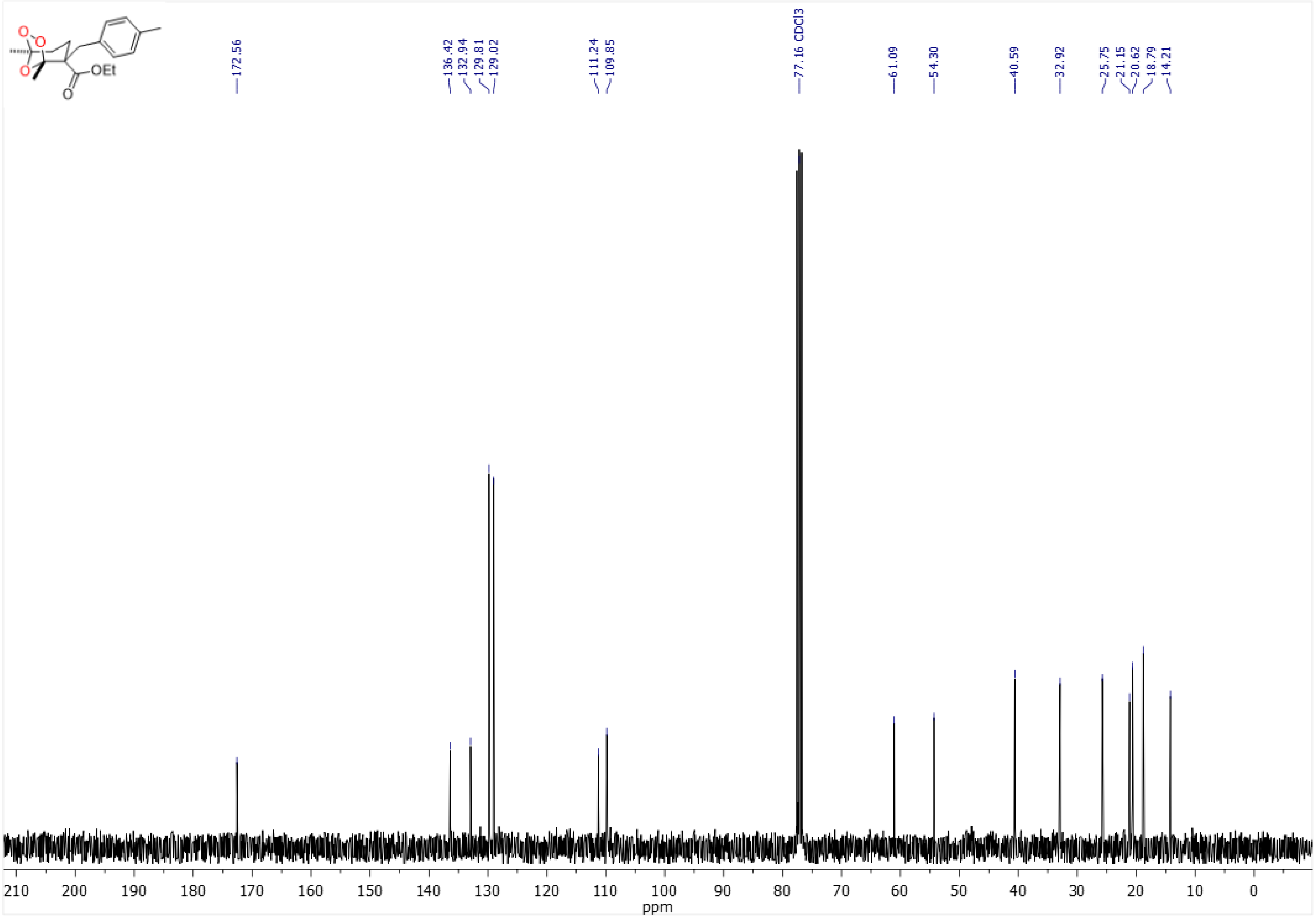

^{1}H NMR (300.13 MHz, $CDCl_3$). Ethyl (1R*,2S*,5S*)-1,5-dimethyl-2-(4-methylbenzyl)-6,7,8-trioxabicyclo[3.2.1]octane-2-carboxylate, 3d

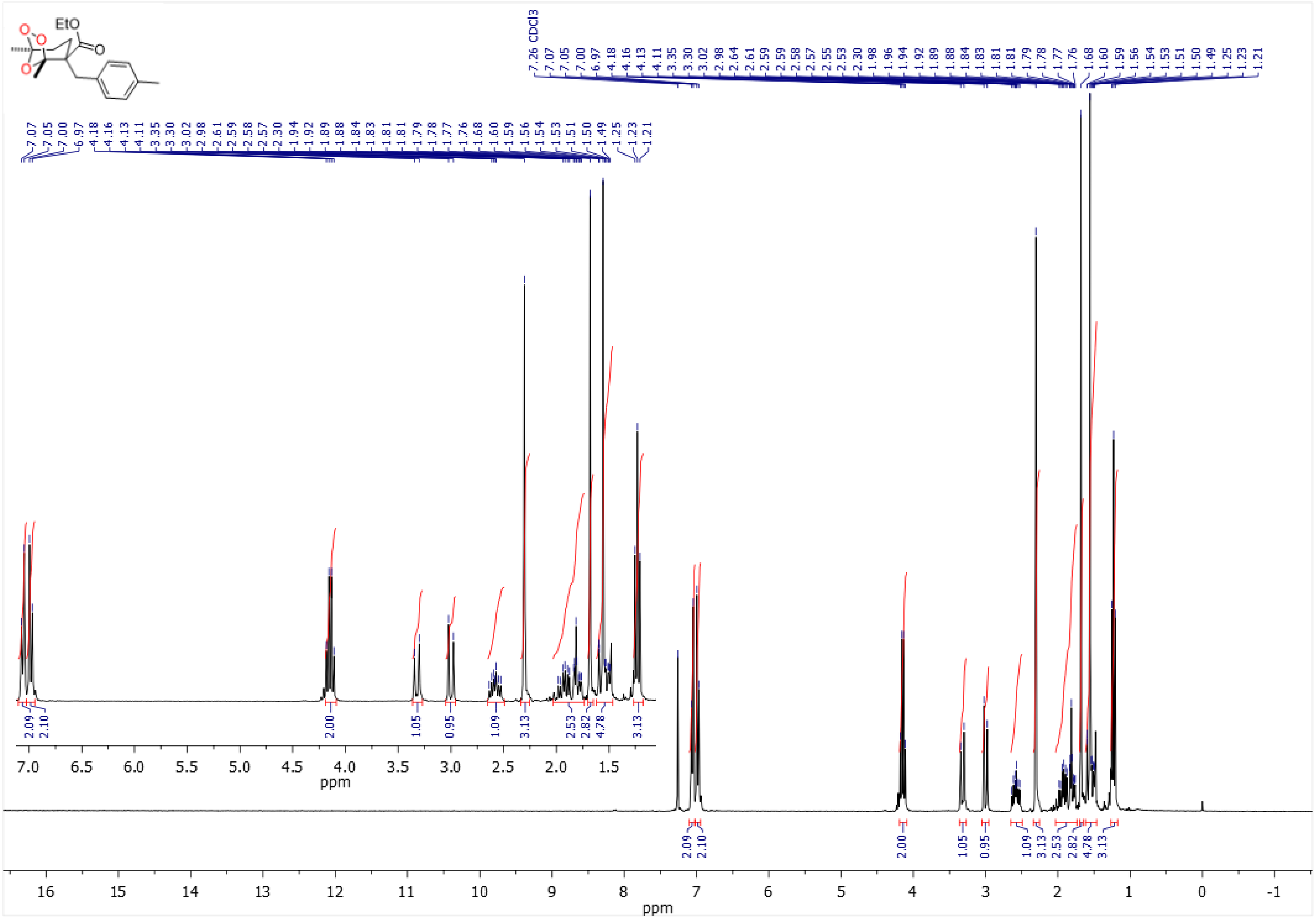

^{13}C NMR (75.48 MHz, CDCl3). Ethyl (1R*,2S*,5S*)-1,5-dimethyl-2-(4-methylbenzyl)-6,7,8-trioxabicyclo[3.2.1]octane-2-carboxylate, 3d

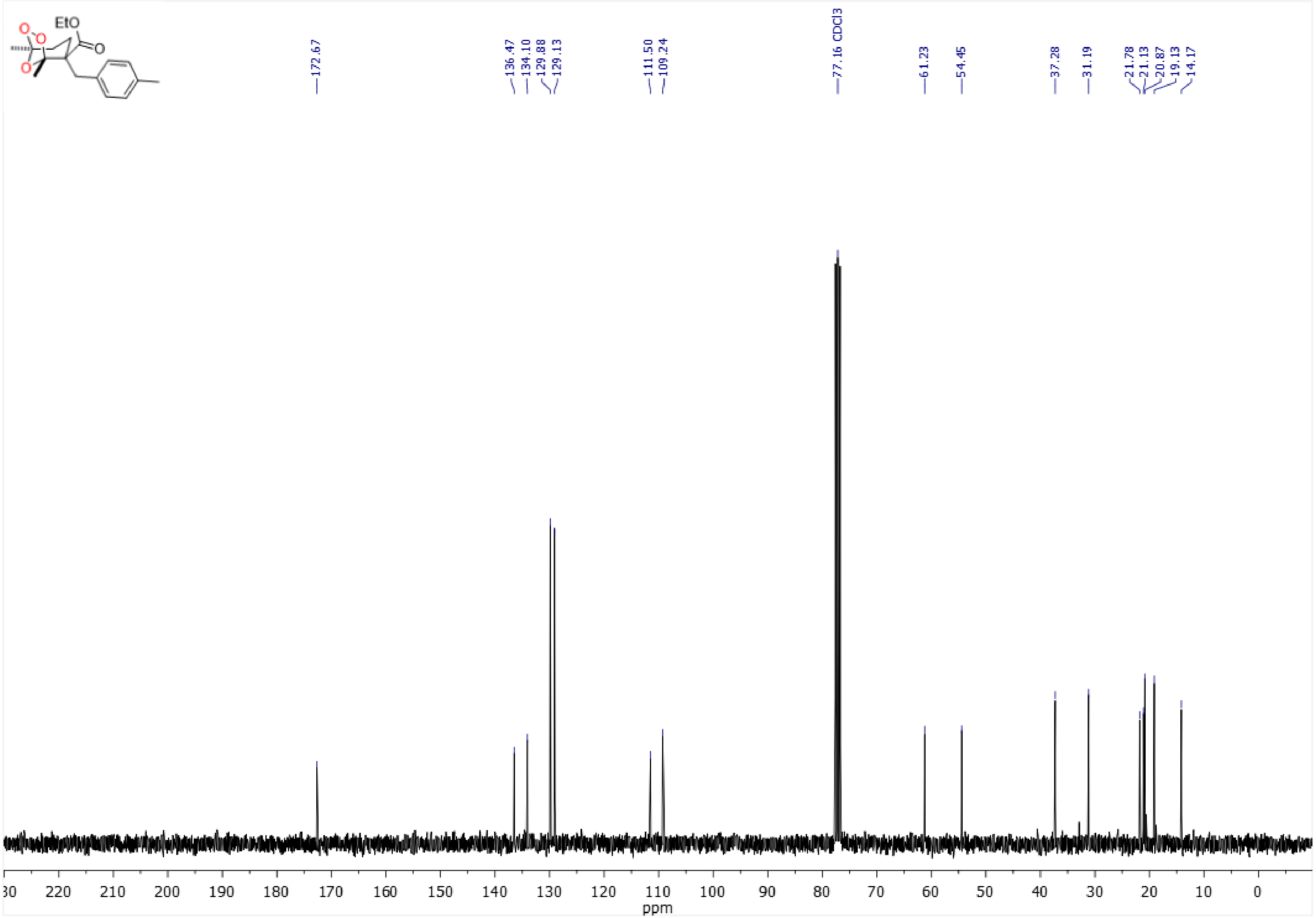

^{1}H NMR (300.13 MHz, CDCl3). Ethyl (1*R**,2*S**,5*S**)-1,5-dimethyl-2-(4-nitrobenzyl)-6,7,8-trioxabicyclo[3.2.1]octane-2-carboxylate, 2e

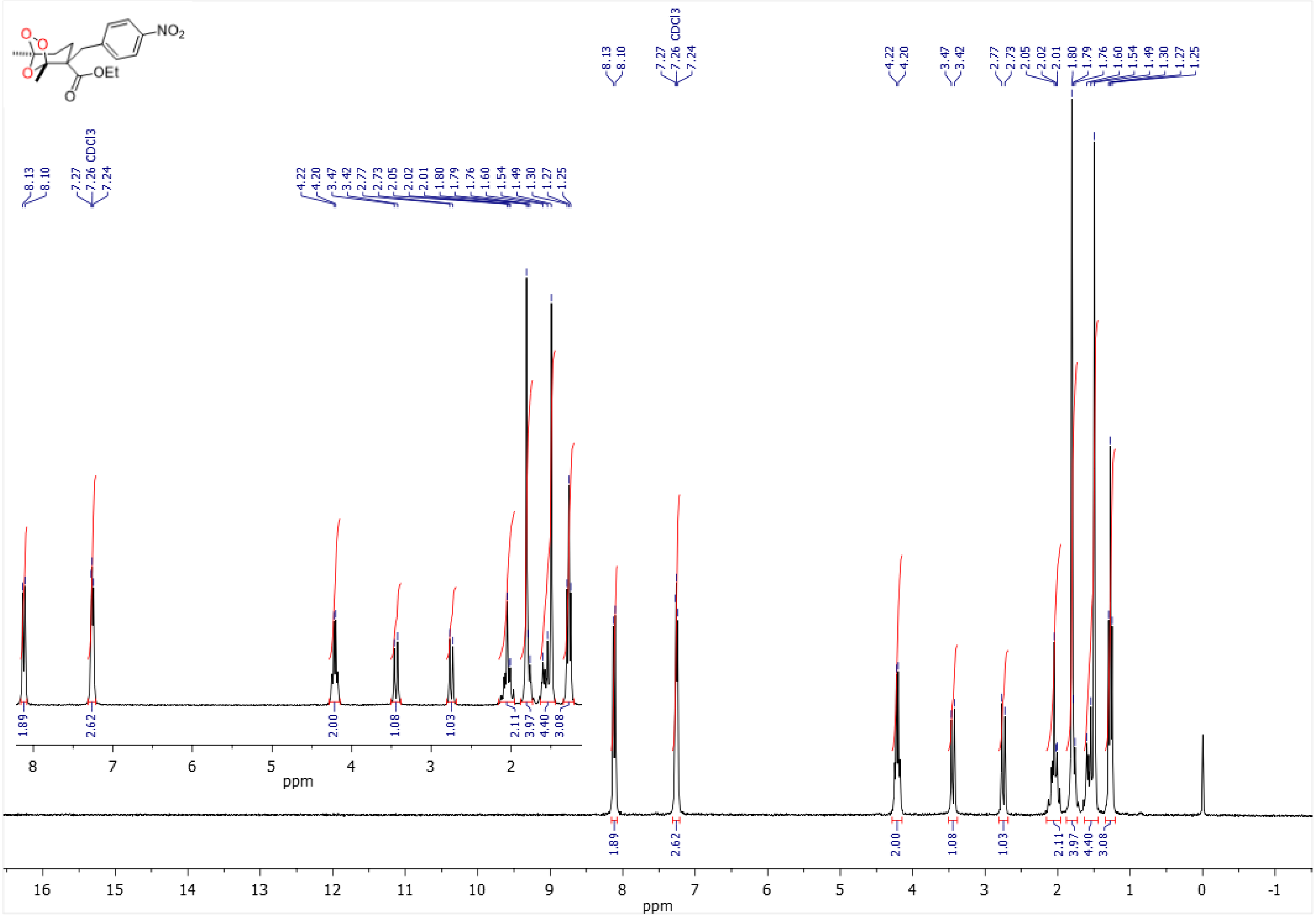

^{13}C NMR (75.48 MHz, $CDCl_3$). Ethyl (1*R,2*S**,5*S**)-1,5-dimethyl-2-(4-nitrobenzyl)-6,7,8-trioxabicyclo[3.2.1]octane-2-carboxylate, 2e**

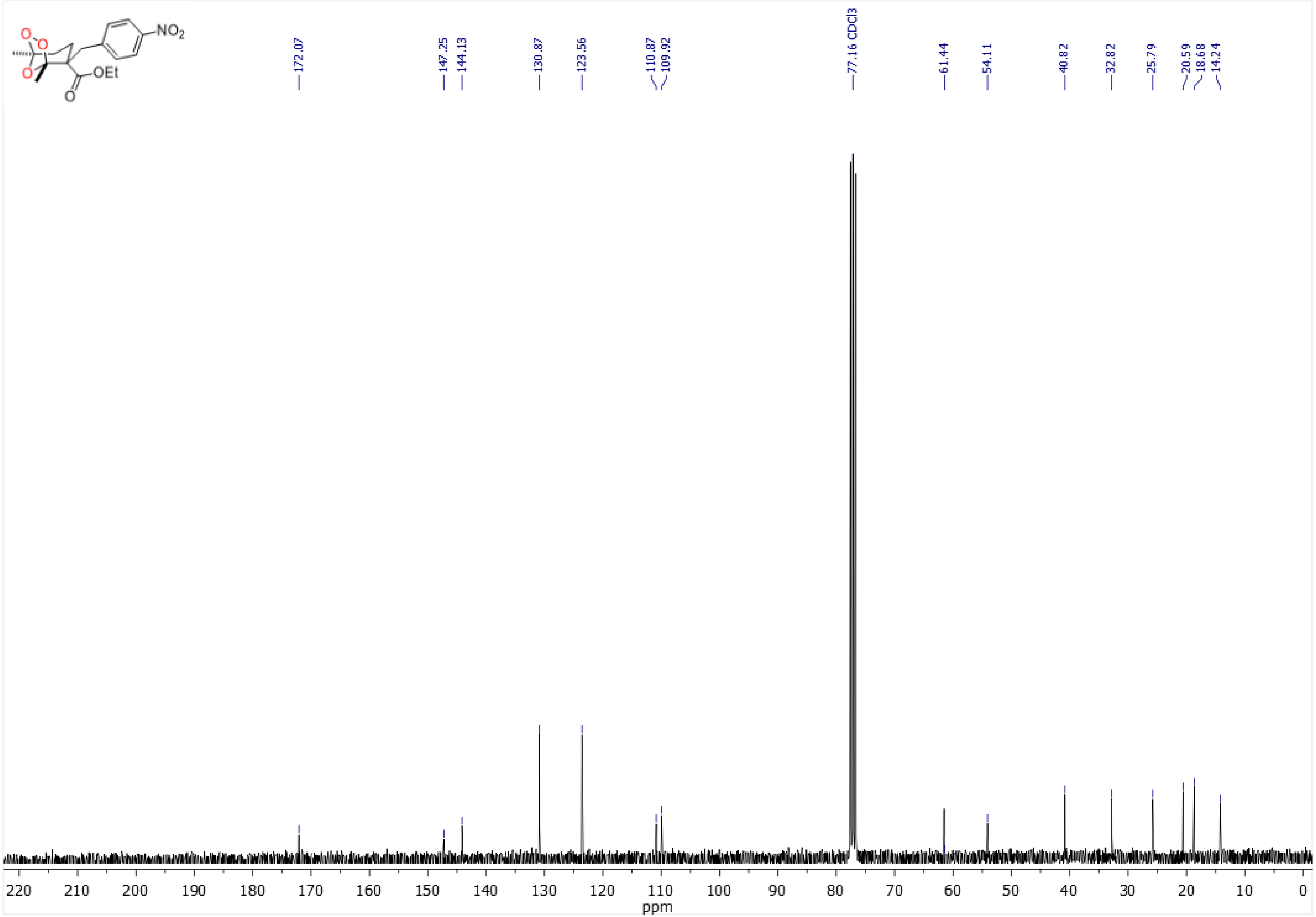

^{1}H NMR (300.13 MHz, $CDCl_3$). Ethyl (1*R,2*S**,5*S**)-1,5-dimethyl-2-(4-nitrobenzyl)-6,7,8-trioxabicyclo[3.2.1]octane-2-carboxylate, 3e**

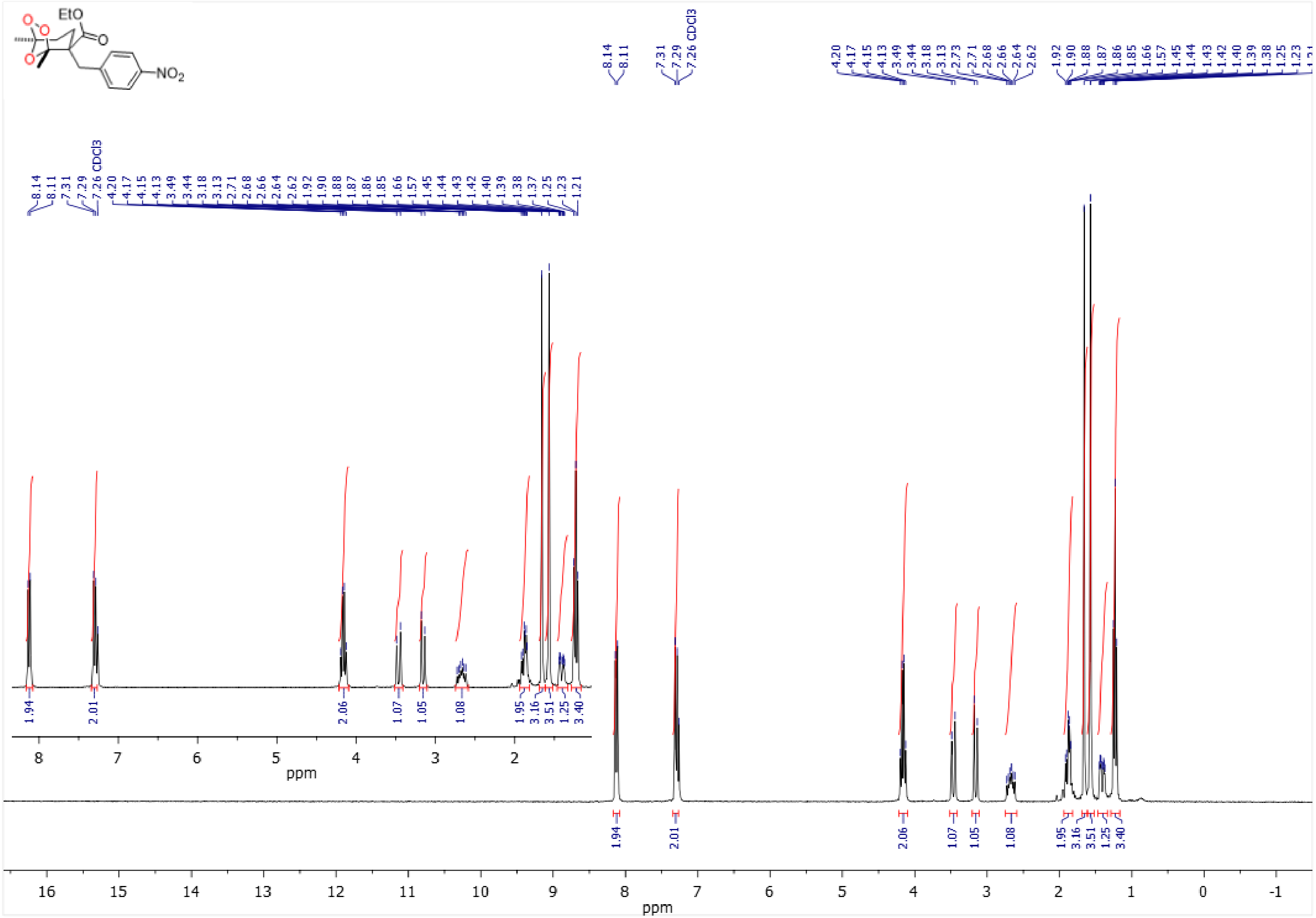

^{13}C NMR (75.48 MHz, $CDCl_3$). Ethyl (1*R**,2*S**,5*S**)-1,5-dimethyl-2-(4-nitrobenzyl)-6,7,8-trioxabicyclo[3.2.1]octane-2-carboxylate, 3e

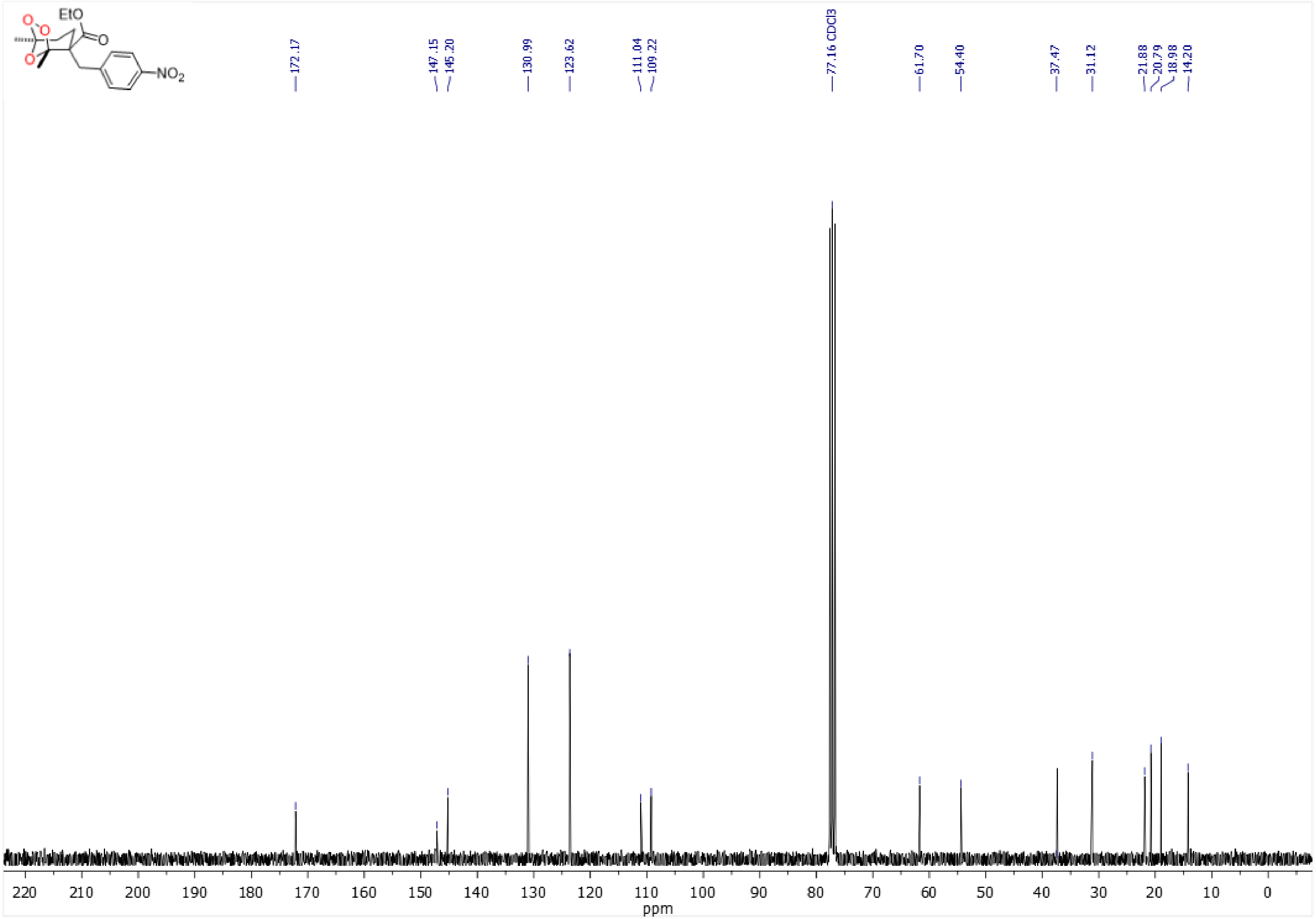

^{1}H NMR (300.13 MHz, $CDCl_3$). Ethyl (1*R**,2*S**,5*S**)-1,5-dimethyl-6,7,8-trioxabicyclo[3.2.1]octane-2-carboxylate, 2f

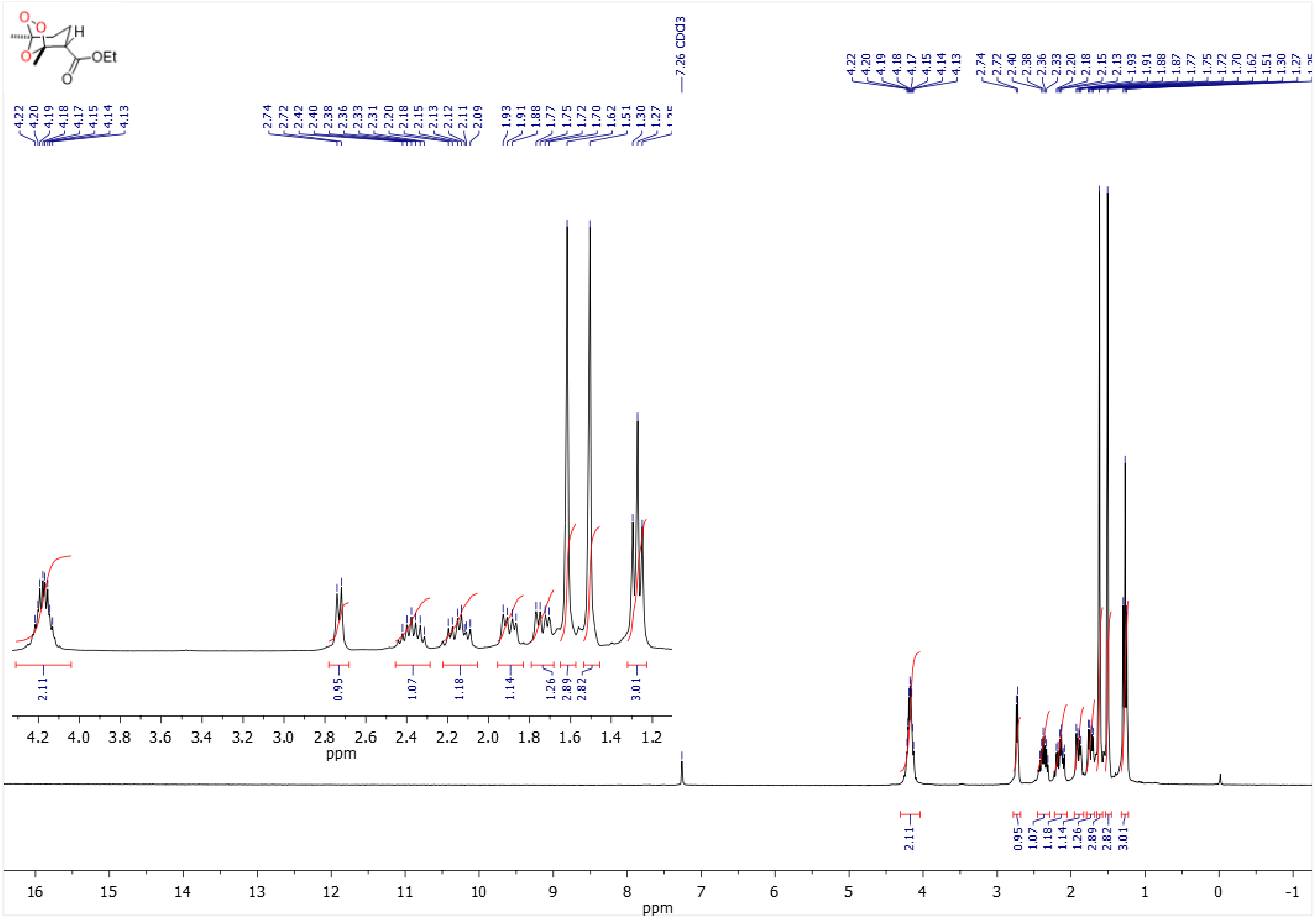

^{13}C NMR (75.48 MHz, $CDCl_3$). Ethyl (1*R**,2*S**,5*S**)-1,5-dimethyl-6,7,8-trioxabicyclo[3.2.1]octane-2-carboxylate, 2f

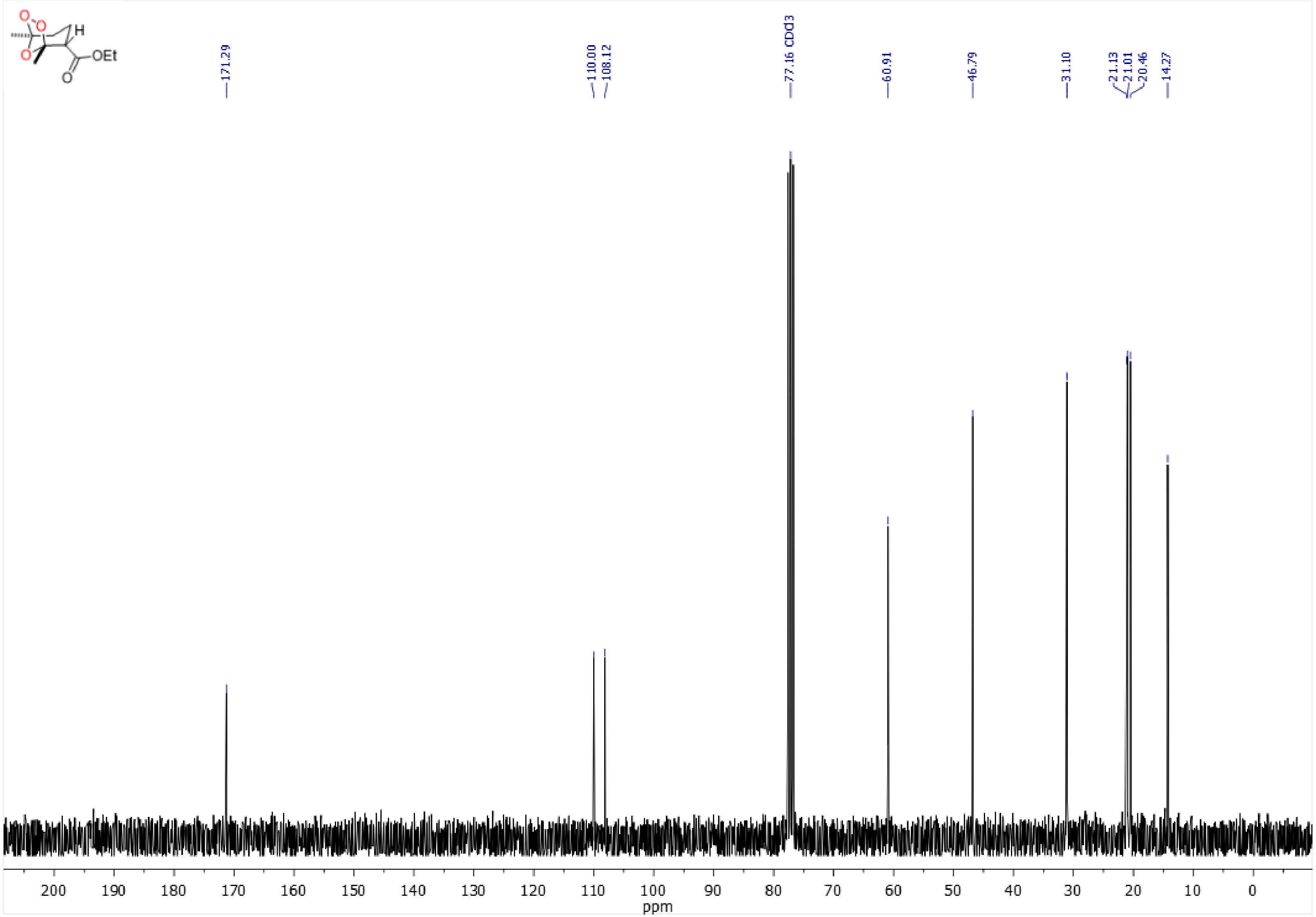

^{1}H NMR (300.13 MHz, $CDCl_3$). Ethyl (1*R**,2*R**,5*S**)-1,5-dimethyl-6,7,8-trioxabicyclo[3.2.1]octane-2-carboxylate, 3f

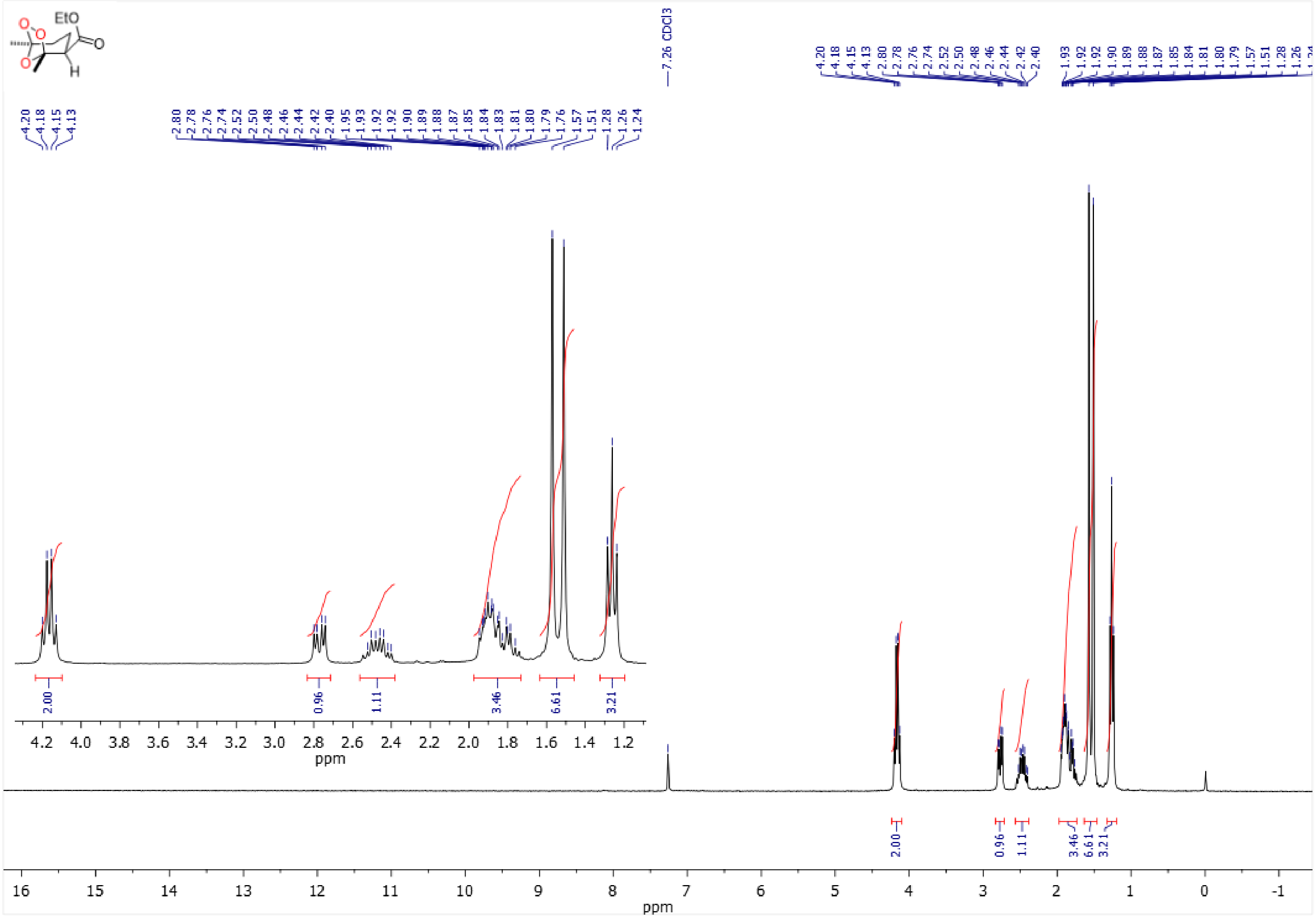

^{13}C NMR (75.48 MHz, CDCl₃). Ethyl (1*R,2*R**,5*S**)-1,5-dimethyl-6,7,8-trioxabicyclo[3.2.1]octane-2-carboxylate, 3f**

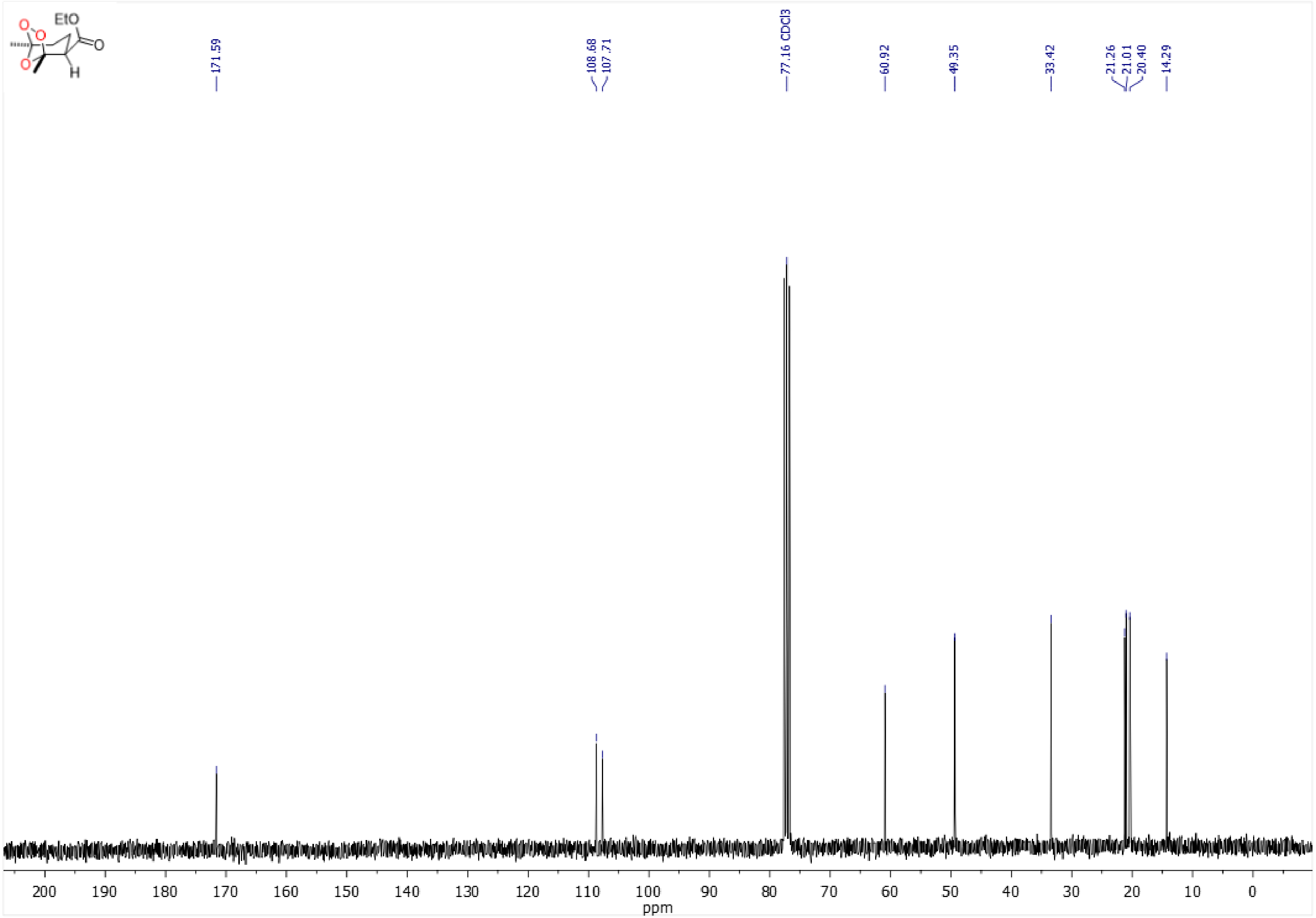

^{1}H NMR (300.13 MHz, CDCl$_3$). Ethyl (1*R**,2*S**,5*S**)-2-etyl-1,5-dimethyl-6,7,8-trioxabicyclo[3.2.1]octane-2-carboxylate, 2g

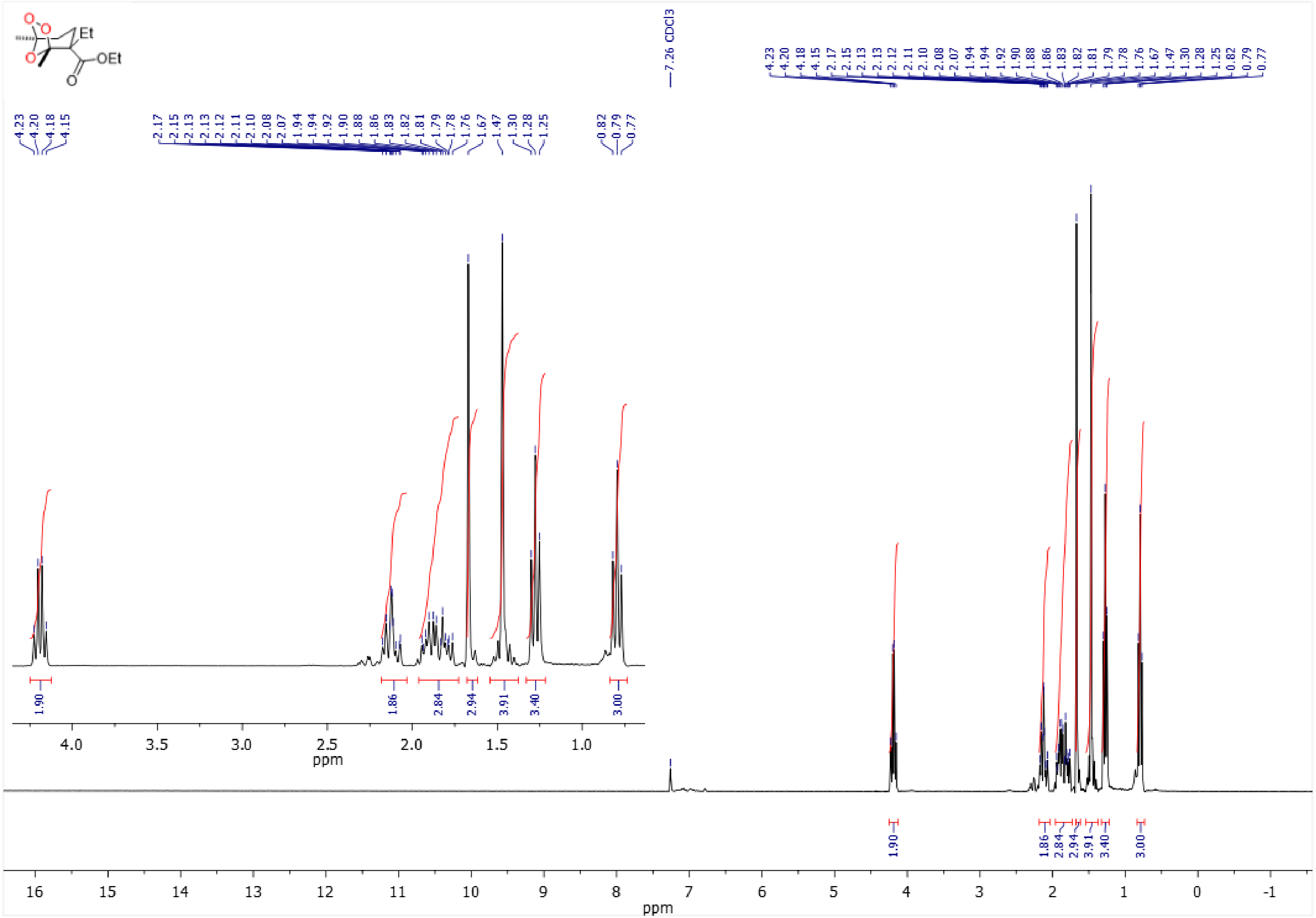

^{13}C NMR (75.48 MHz, CDCl₃). Ethyl (1*R**,2*S**,5*S**)-2-etyl-1,5-dimethyl-6,7,8-trioxabicyclo[3.2.1]octane-2-carboxylate, 2g

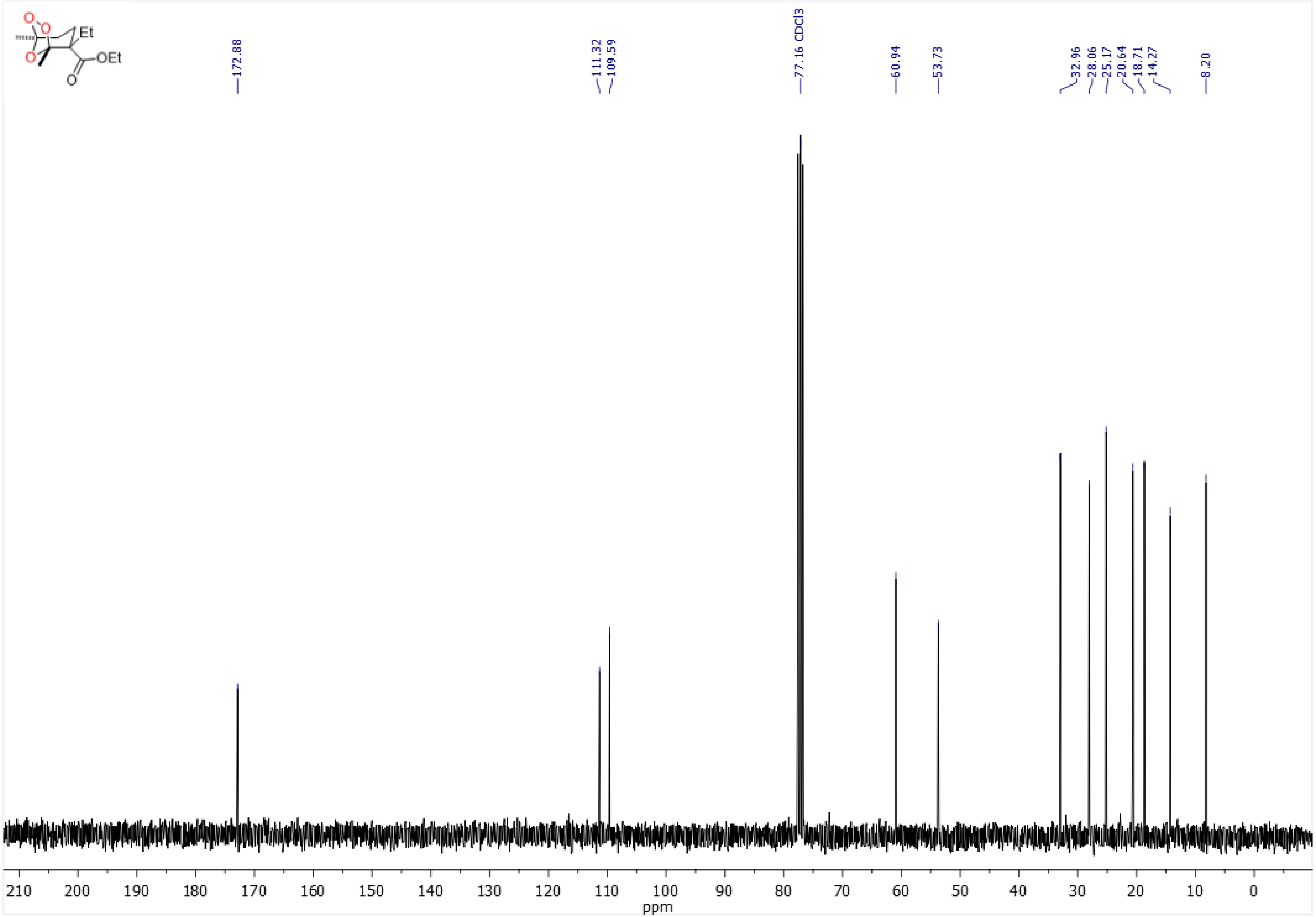

^{1}H NMR (300.13 MHz, $CDCl_3$). Ethyl (1*R**,2*R**,5*S**)-2-ethyl-1,5-dimethyl-6,7,8-trioxabicyclo[3.2.1]octane-2-carboxylate, 3g

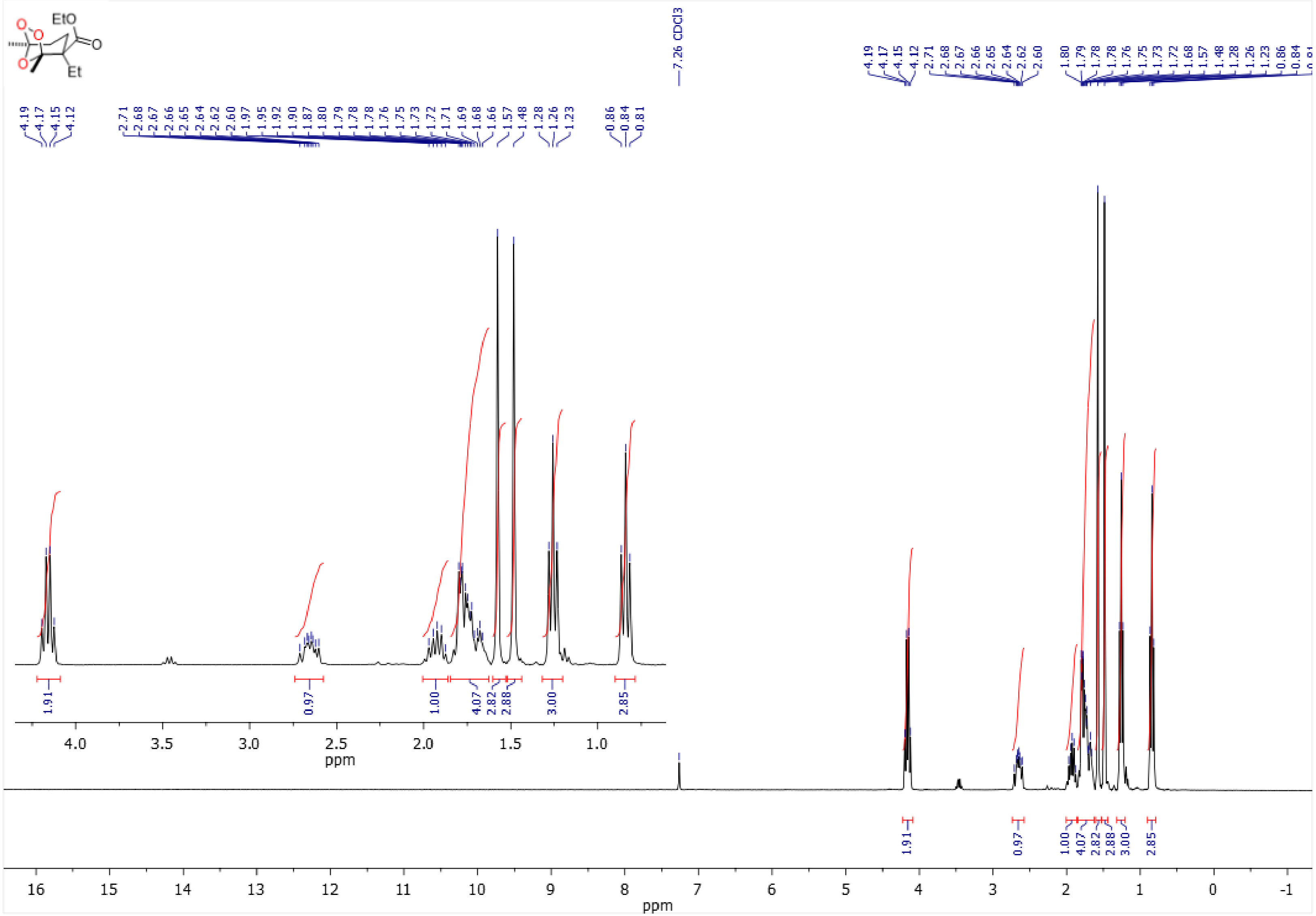

^{13}C NMR (75.48 MHz, $CDCl_3$). Ethyl (1*R**,2*R**,5*S**)-2-ethyl-1,5-dimethyl-6,7,8-trioxabicyclo[3.2.1]octane-2-carboxylate, 3g

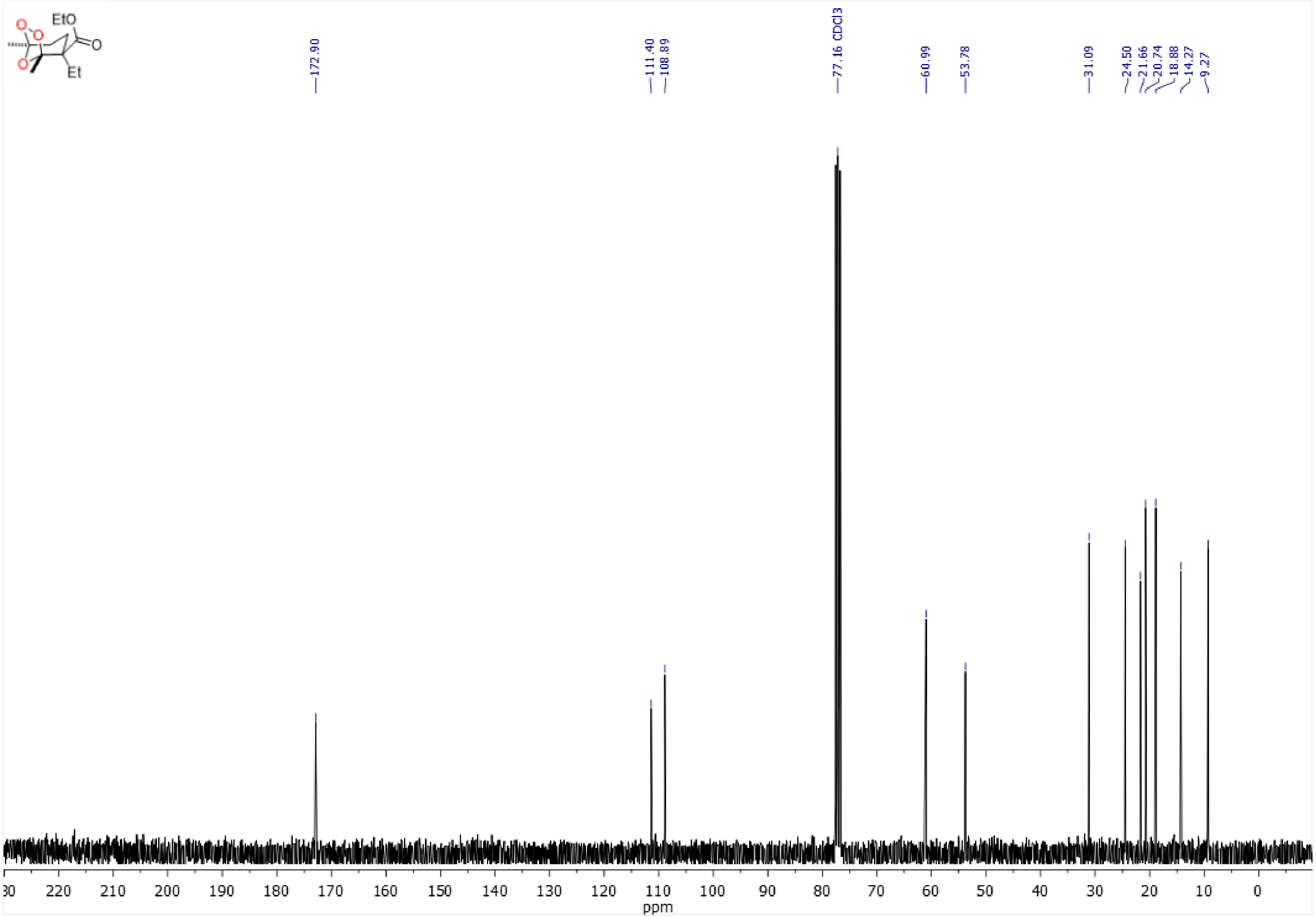

^{1}H NMR (300.13 MHz, $CDCl_3$). Ethyl (1*R**,2*S**,5*S**)-2-butyl-1,5-dimethyl-6,7,8-trioxabicyclo[3.2.1]octane-2-carboxylate, 2h

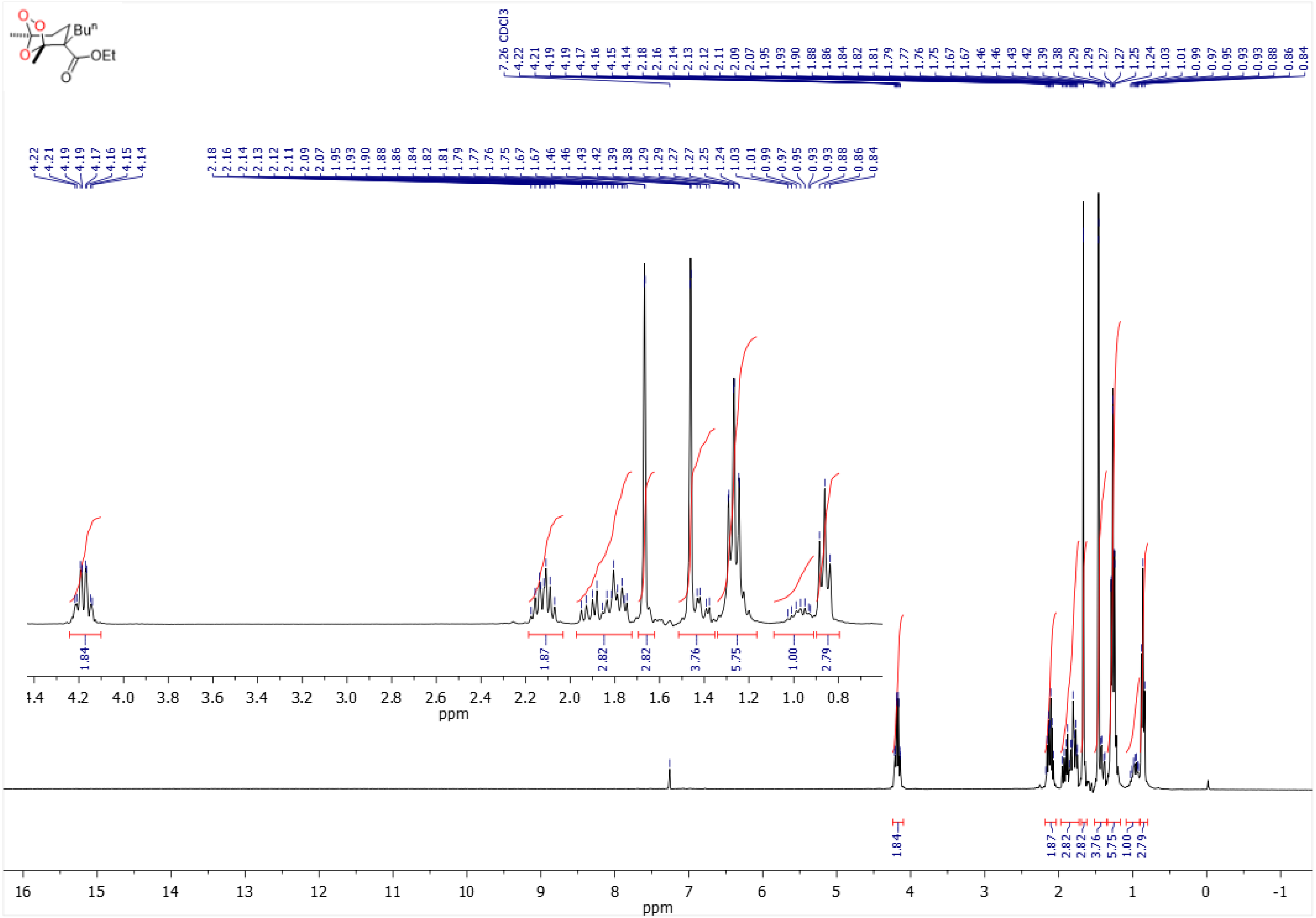

^{13}C NMR (75.48 MHz, $CDCl_3$). Ethyl (1*R**,2*S**,5*S**)-2-butyl-1,5-dimethyl-6,7,8-trioxabicyclo[3.2.1]octane-2-carboxylate, 2h

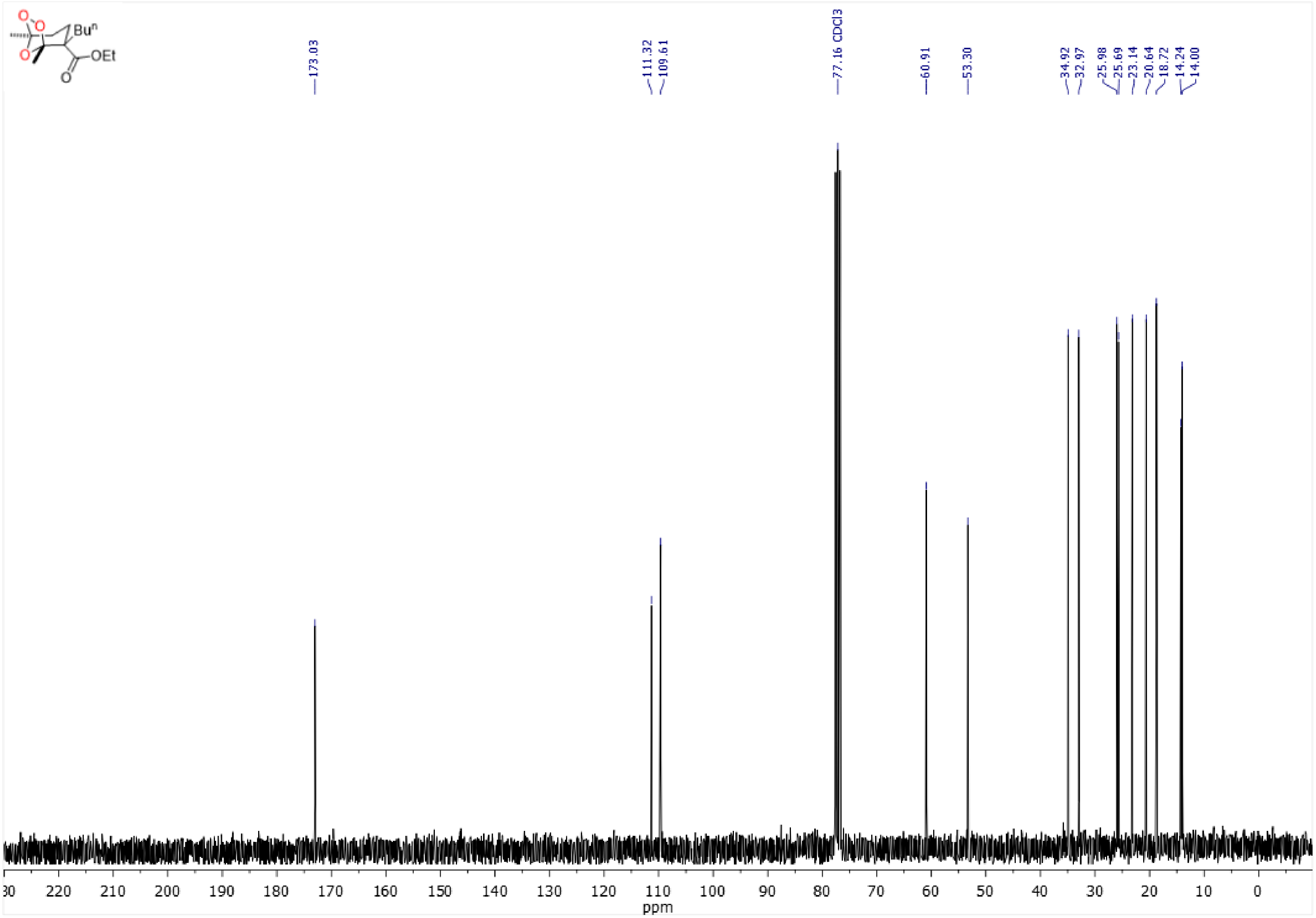

^{1}H NMR (300.13 MHz, $CDCl_3$). Ethyl (1*R**,2*R**,5*S**)-2-butyl-1,5-dimethyl-6,7,8-trioxabicyclo[3.2.1]octane-2-carboxylate, 3h

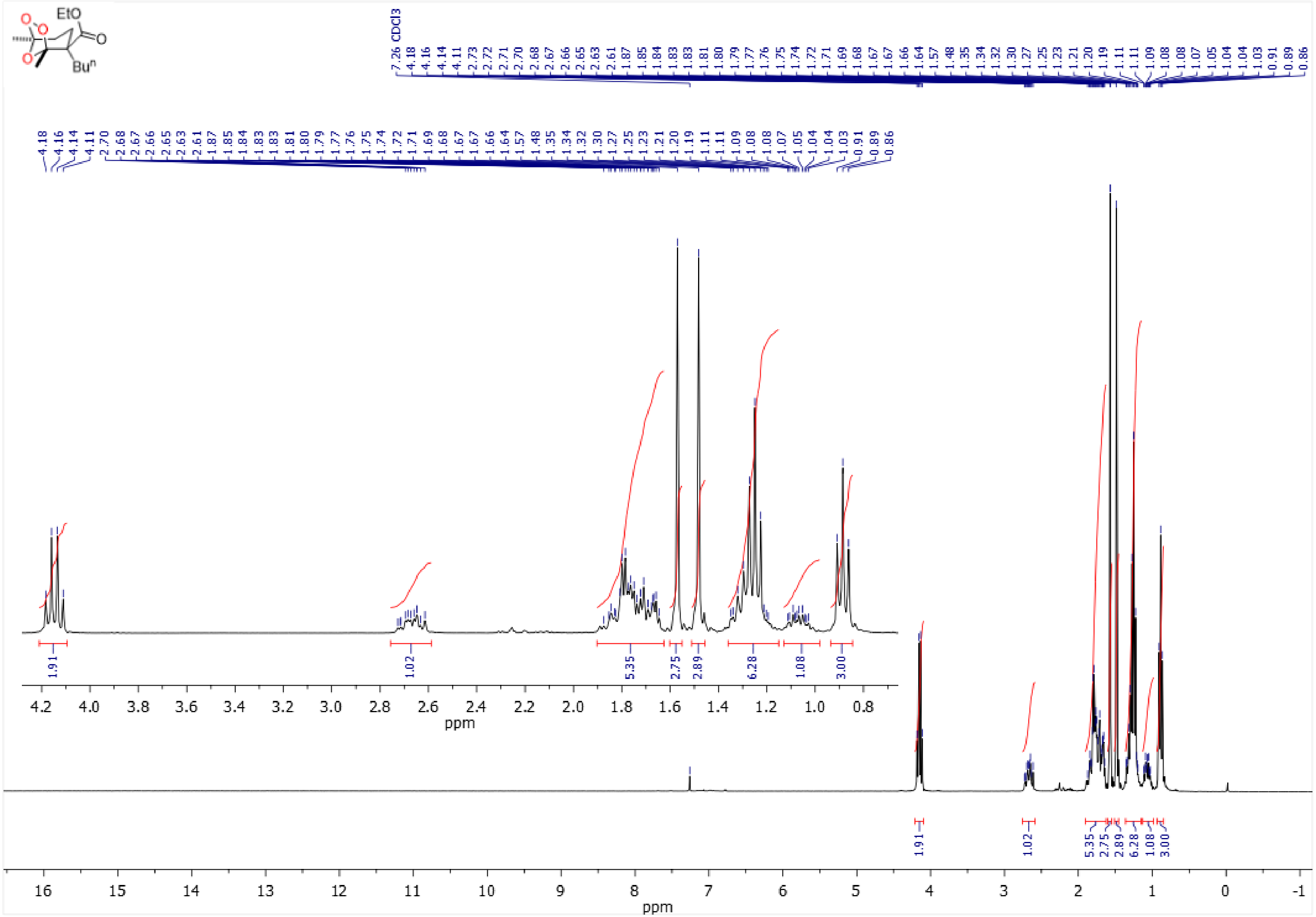

^{13}C NMR (75.48 MHz, CDCl$_3$). Ethyl (1*R**,2*R**,5*S**)-2-butyl-1,5-dimethyl-6,7,8-trioxabicyclo[3.2.1]octane-2-carboxylate, 3h

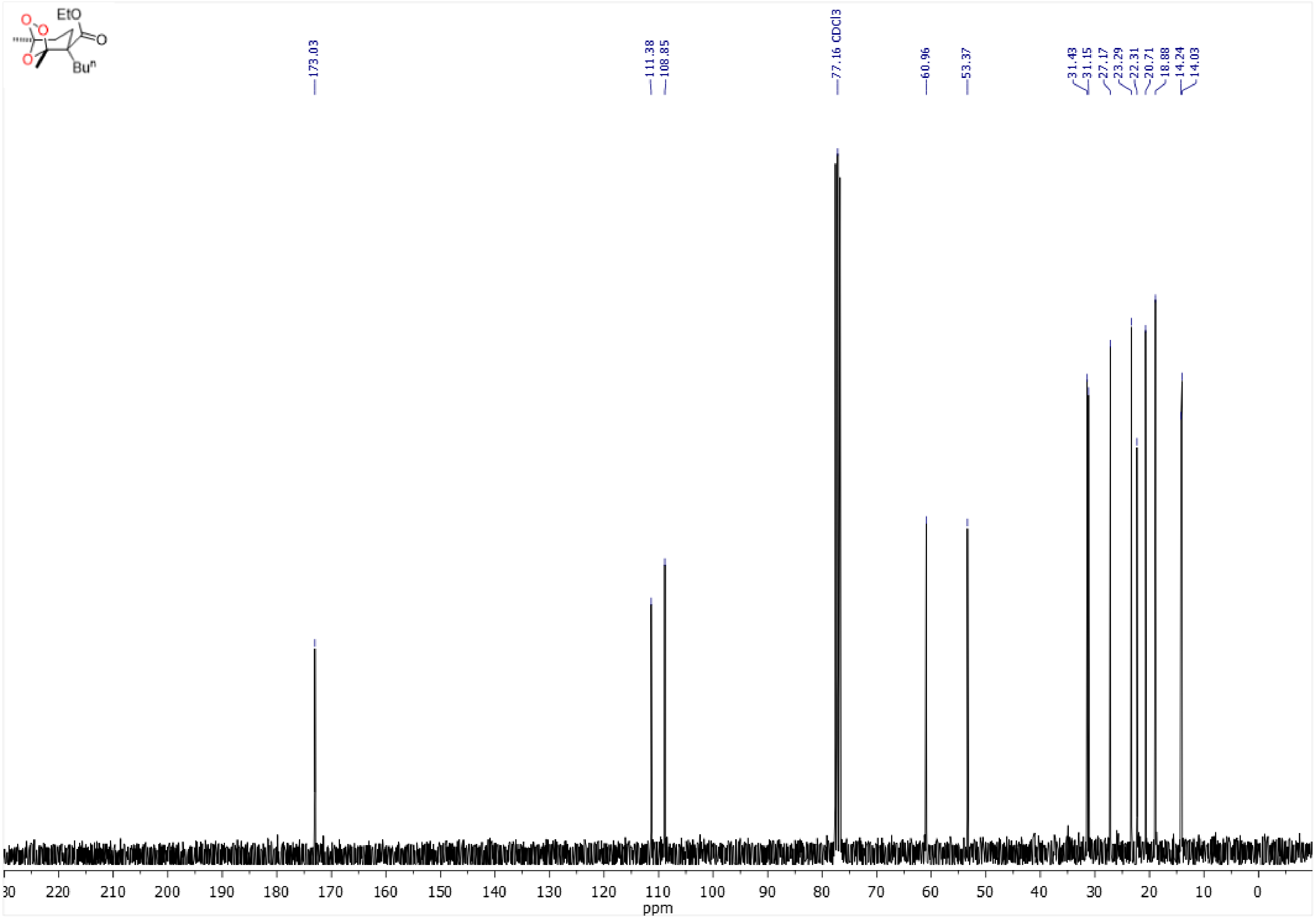

^{1}H NMR (300.13 MHz, $CDCl_3$). Ethyl (1*R**,2*S**,5*S**)-2-hexyl-1,5-dimethyl-6,7,8-trioxabicyclo[3.2.1]octane-2-carboxylate, 2i

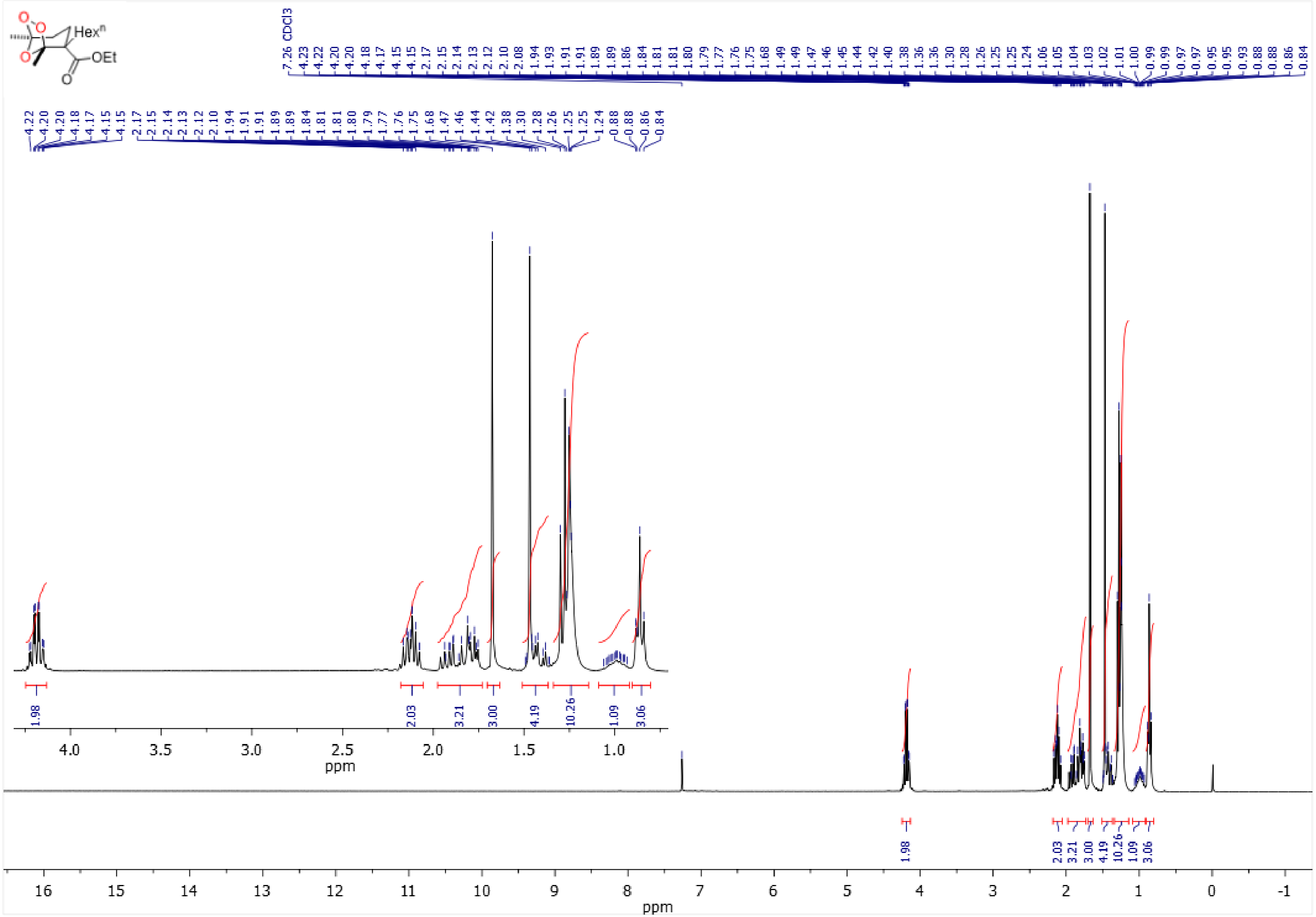

^{13}C NMR (75.48 MHz, $CDCl_3$). Ethyl (1*R,2*S**,5*S**)-2-hexyl-1,5-dimethyl-6,7,8-trioxabicyclo[3.2.1]octane-2-carboxylate, 2i**

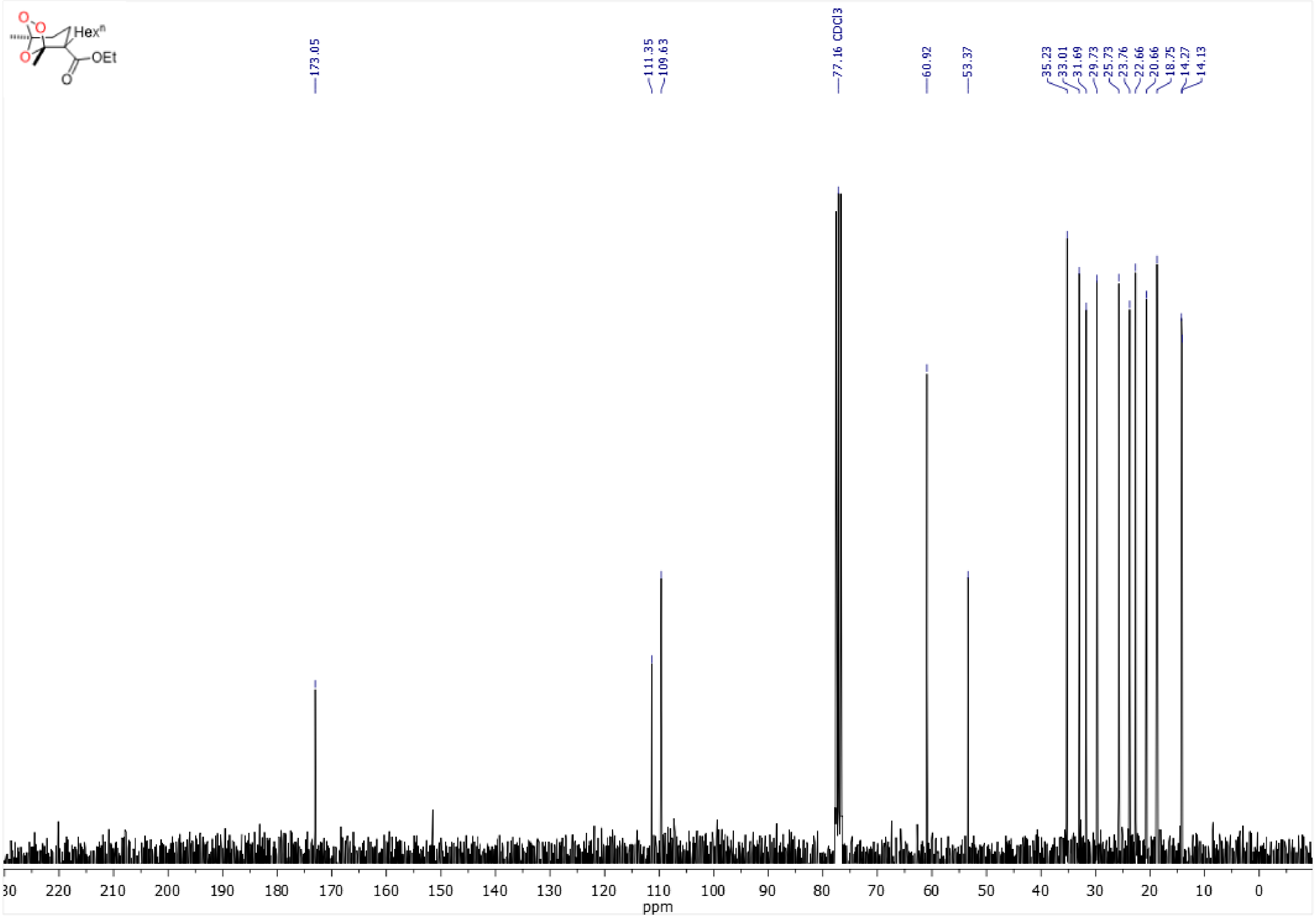

^{1}H NMR (300.13 MHz, CDCl3). Ethyl (1*R**,2*R**,5*S**)-2-hexyl-1,5-dimethyl-6,7,8-trioxabicyclo[3.2.1]octane-2-carboxylate, 3i

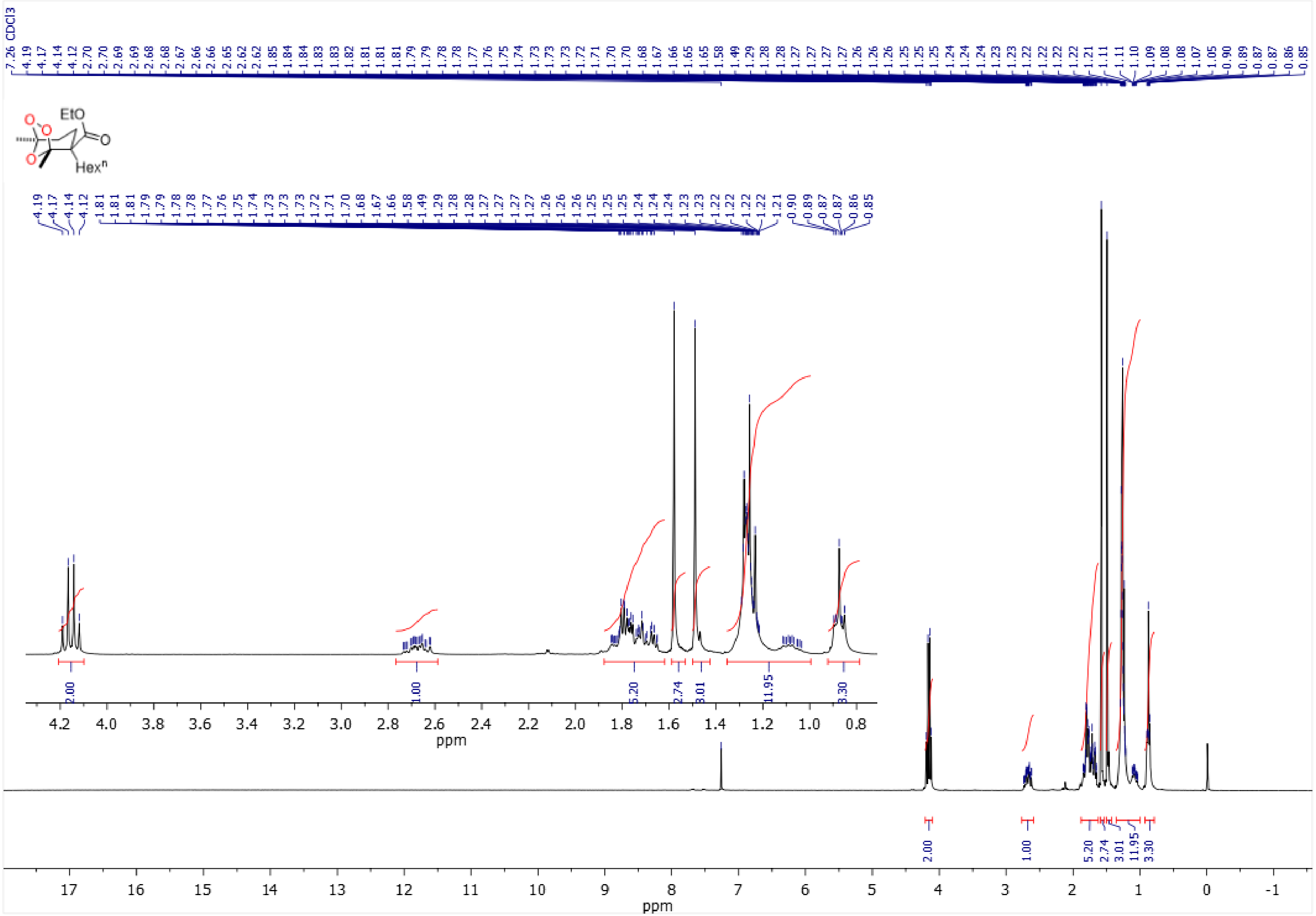

^{13}C NMR (75.48 MHz, $CDCl_3$). Ethyl (1*R**,2*R**,5*S**)-2-hexyl-1,5-dimethyl-6,7,8-trioxabicyclo[3.2.1]octane-2-carboxylate, 3i

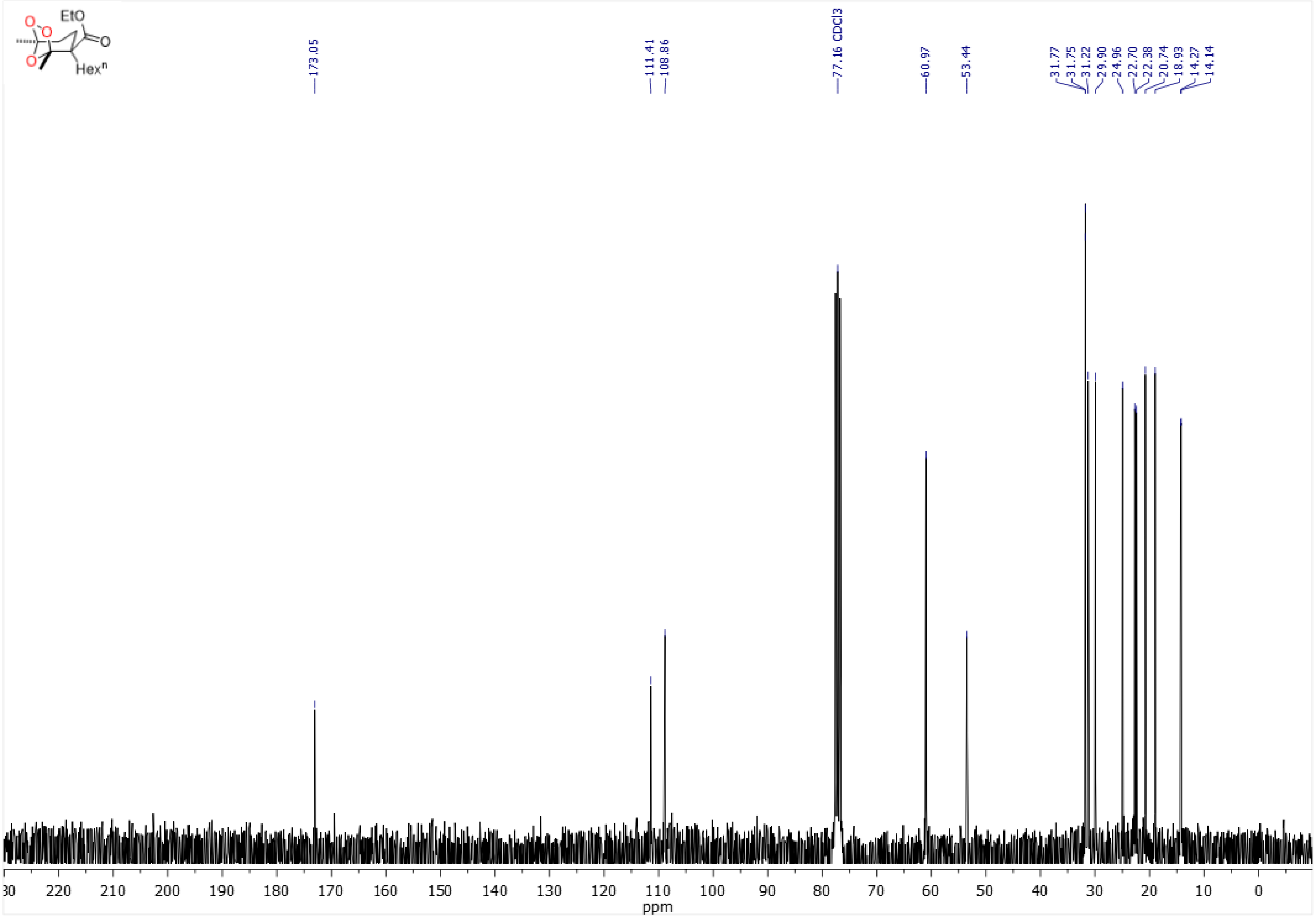

^{1}H NMR (300.13 MHz, CDCl3). Ethyl (1*R**,2*R**,5*S**)-2-allyl-1,5-dimethyl-6,7,8-trioxabicyclo[3.2.1]octane-2-carboxylate, 2j

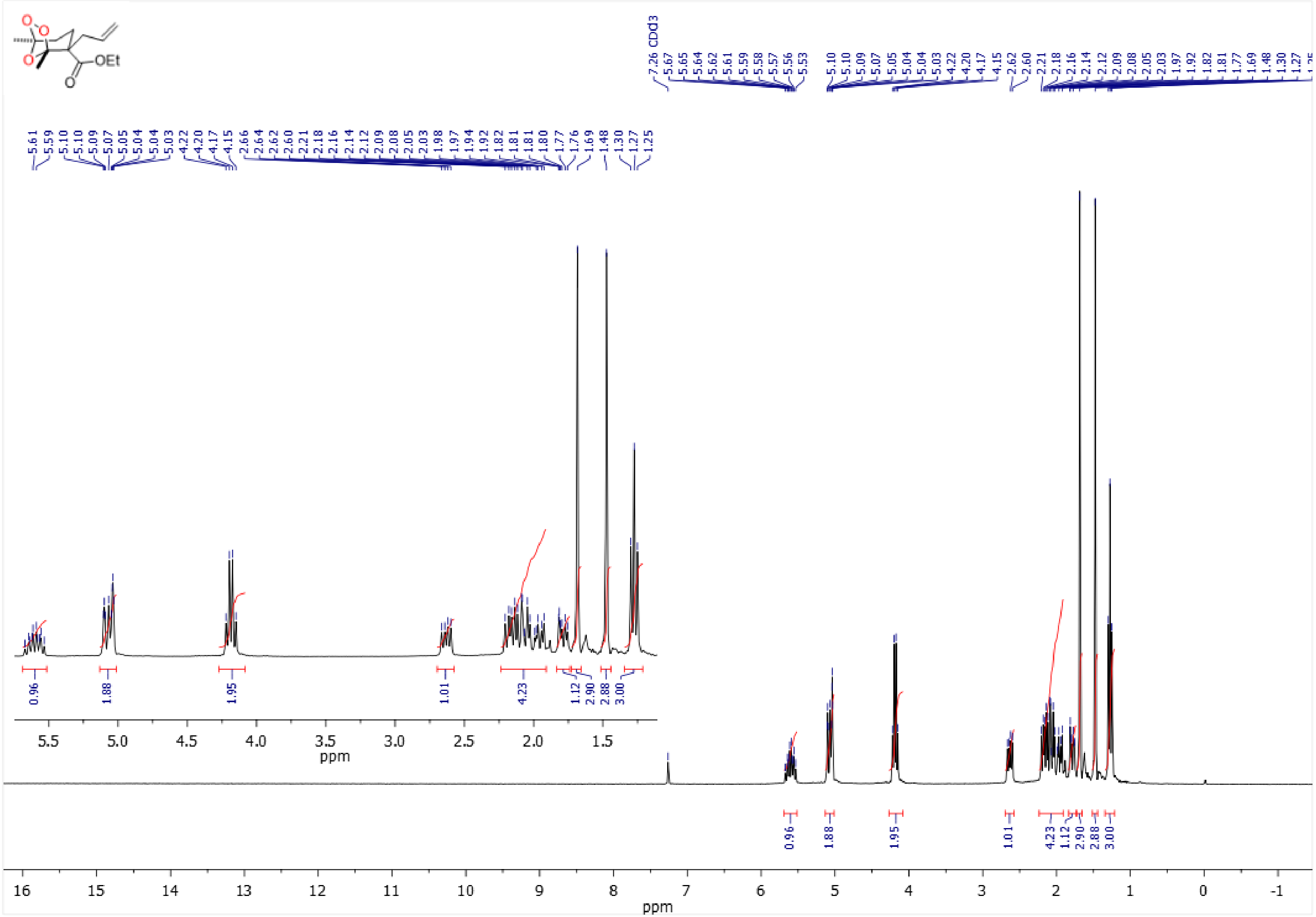

^{13}C NMR (75.48 MHz, CDCl₃). Ethyl (1*R,2*R**,5*S**)-2-allyl-1,5-dimethyl-6,7,8-trioxabicyclo[3.2.1]octane-2-carboxylate, 2j**

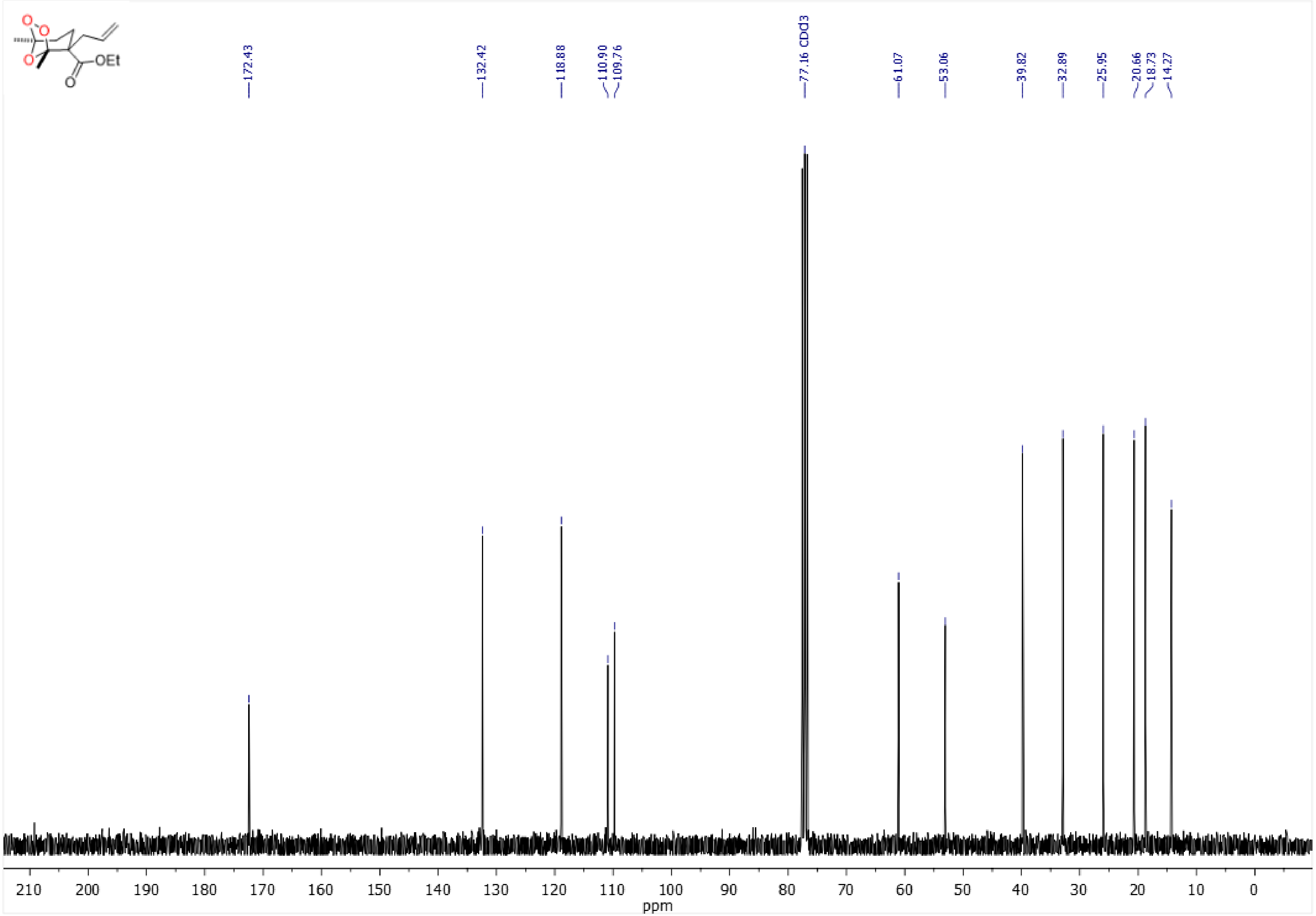

^{1}H NMR (300.13 MHz, CDCl$_3$). Ethyl (1*R**,2*S**,5*S**)-2-allyl-1,5-dimethyl-6,7,8-trioxabicyclo[3.2.1]octane-2-carboxylate, 3j

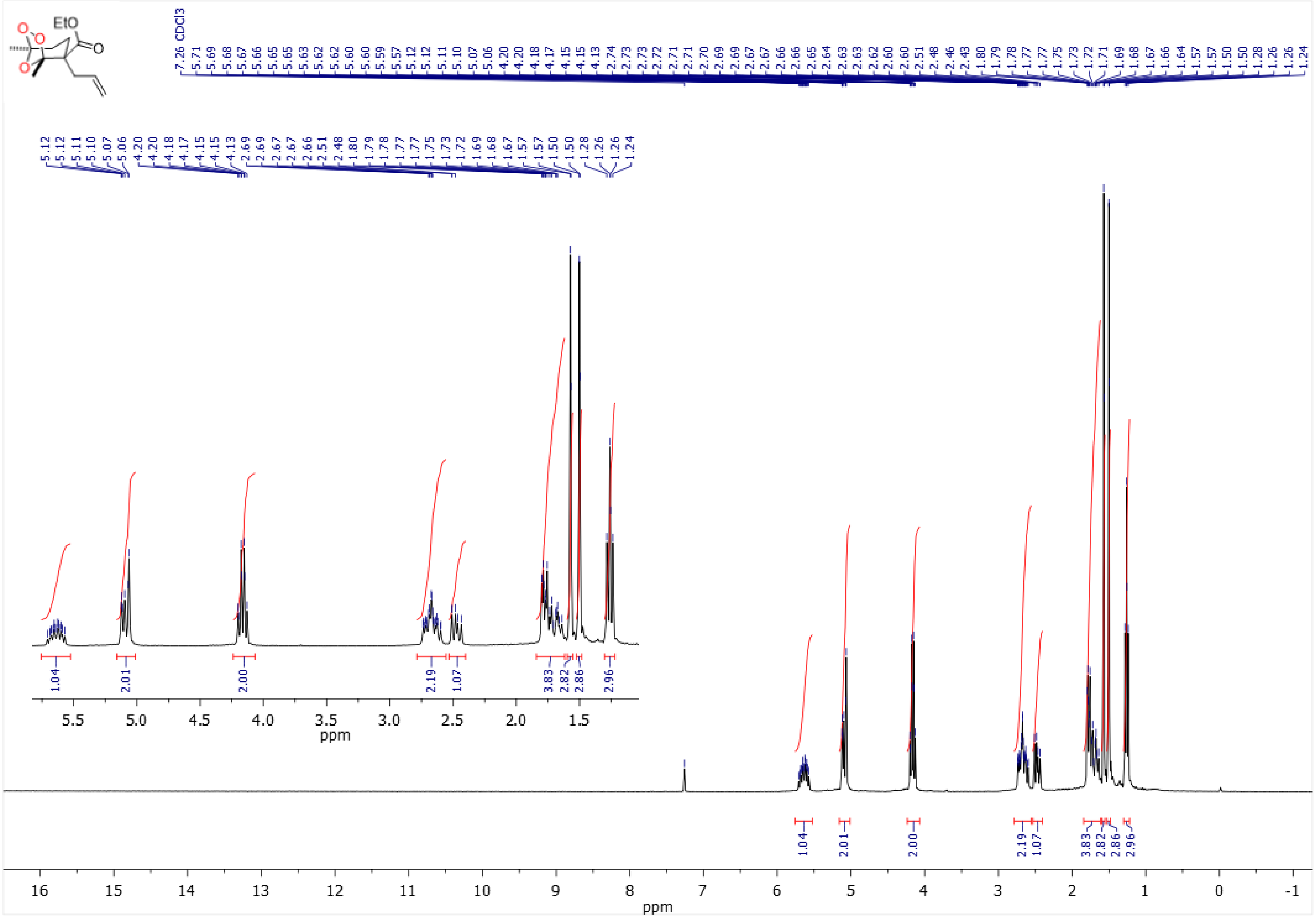

^{13}C NMR (75.48 MHz, CDCl$_3$). Ethyl (1*R,2*S**,5*S**)-2-allyl-1,5-dimethyl-6,7,8-trioxabicyclo[3.2.1]octane-2-carboxylate, 3j**

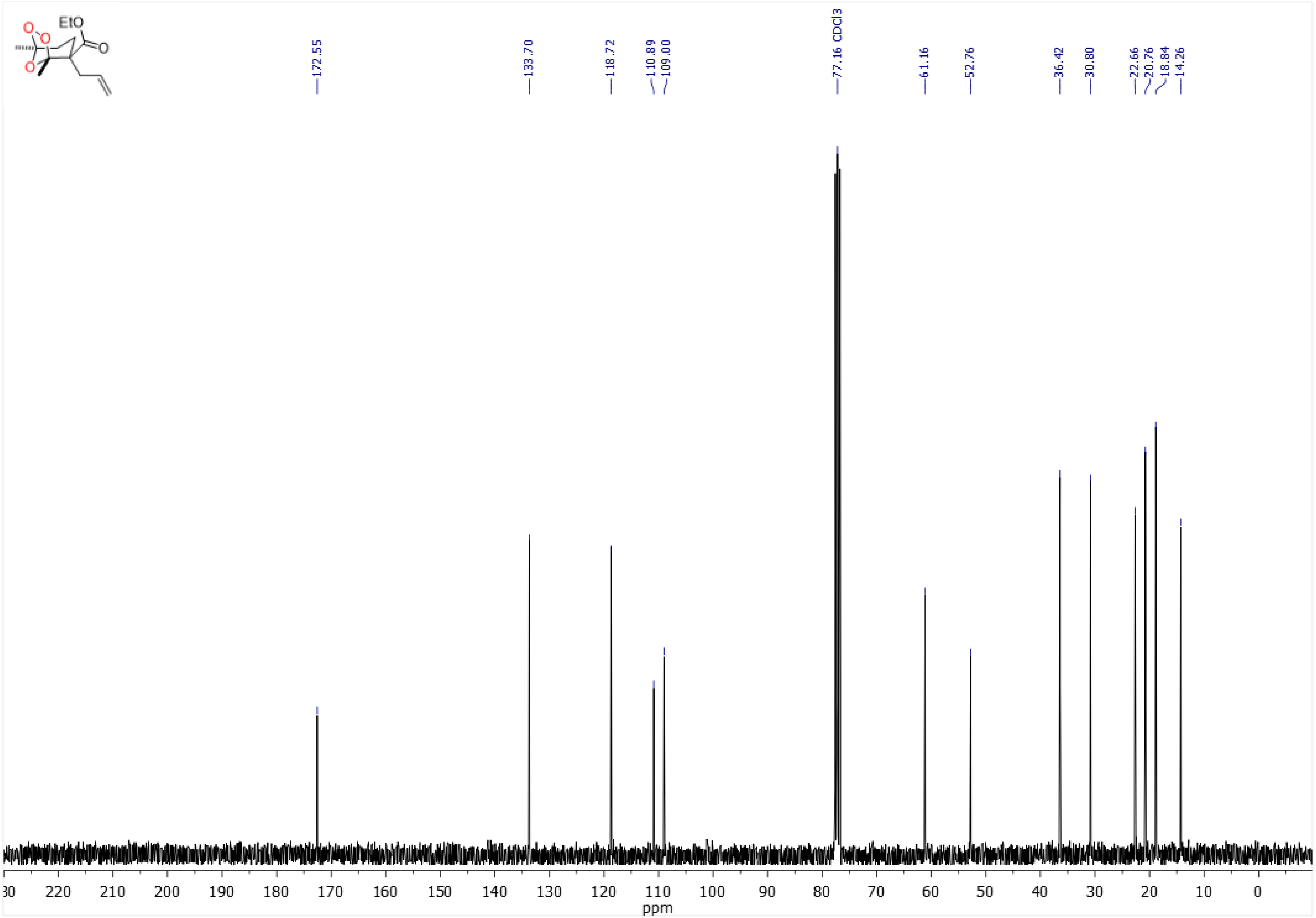

^{1}H NMR (300.13 MHz, $CDCl_3$). Ethyl(1*R**,2*S**,5*S**)-2-(2-cyanoethyl)-1,5-dimethyl-6,7,8-trioxabicyclo[3.2.1]octane-2-carboxylate, 2k

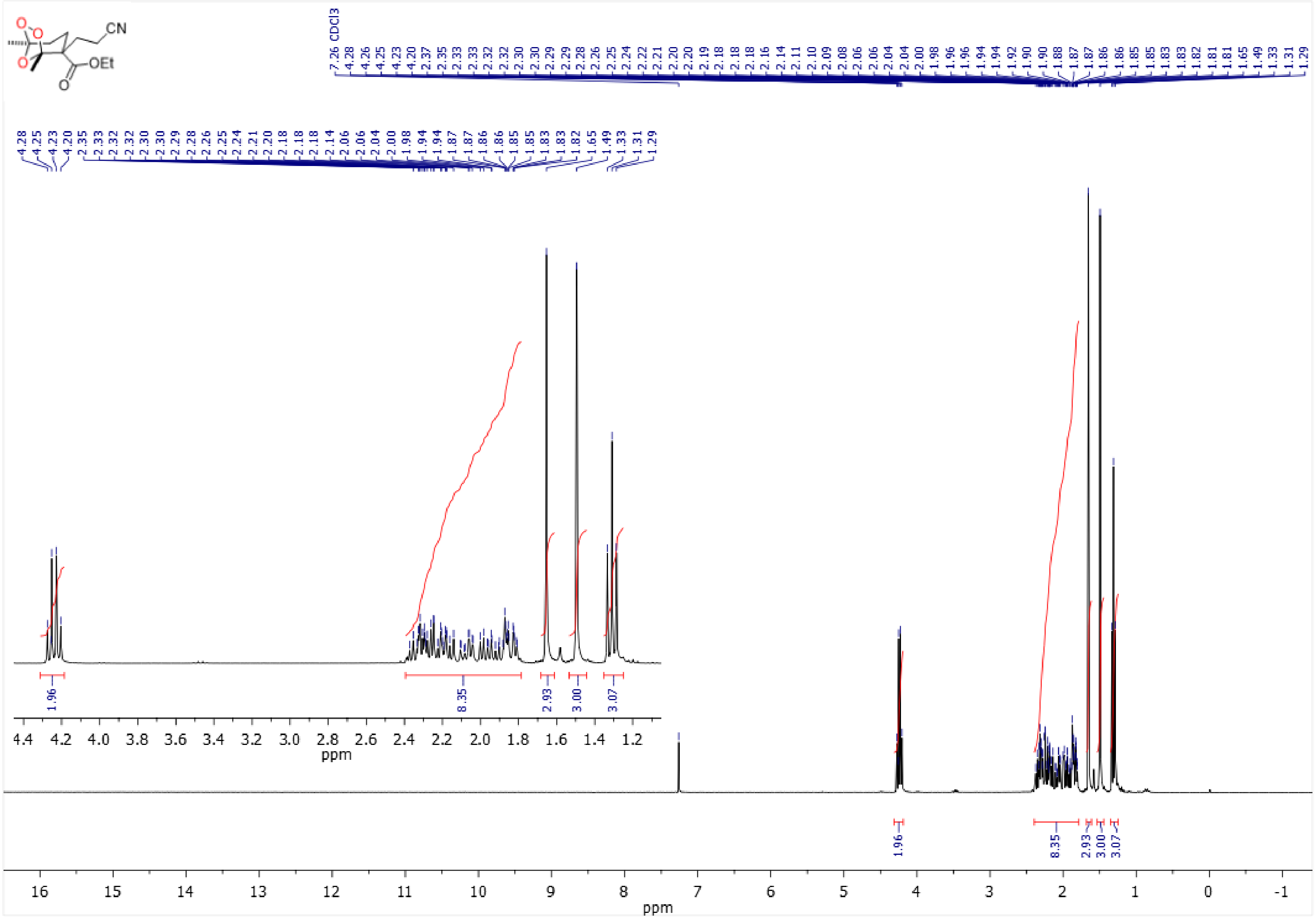

^{13}C NMR (75.48 MHz, CDCl$_3$). Ethyl(1*R**,2*S**,5*S**)-2-(2-cyanoethyl)-1,5-dimethyl-6,7,8-trioxabicyclo[3.2.1]octane-2-carboxylate, 2k

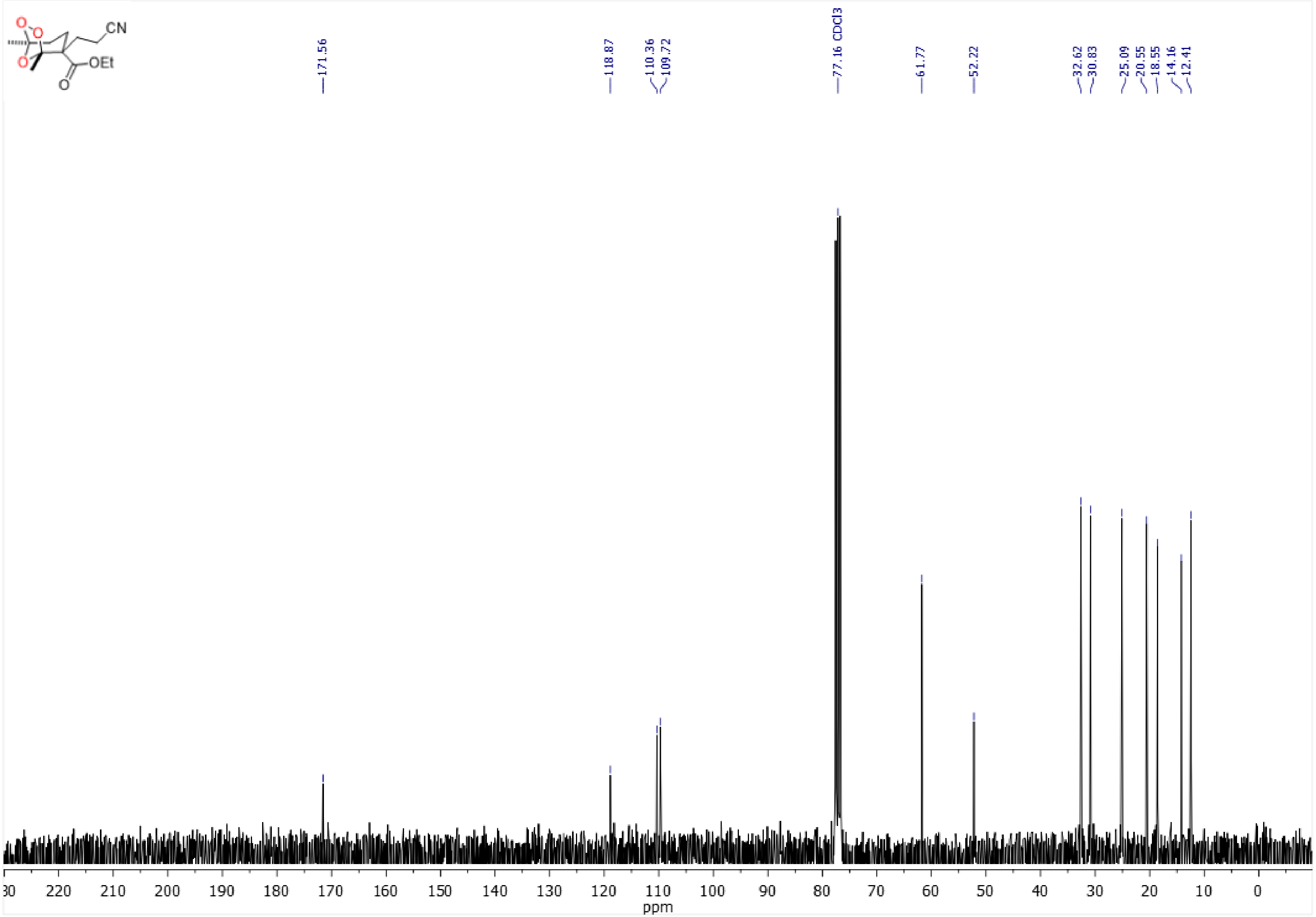

^{1}H NMR (300.13 MHz, CDCl3). Ethyl (1*R**,2*R**,5*S**)-2-(2-cyanoethyl)-1,5-dimethyl-6,7,8-trioxabicyclo[3.2.1]octane-2-carboxylate, 3k

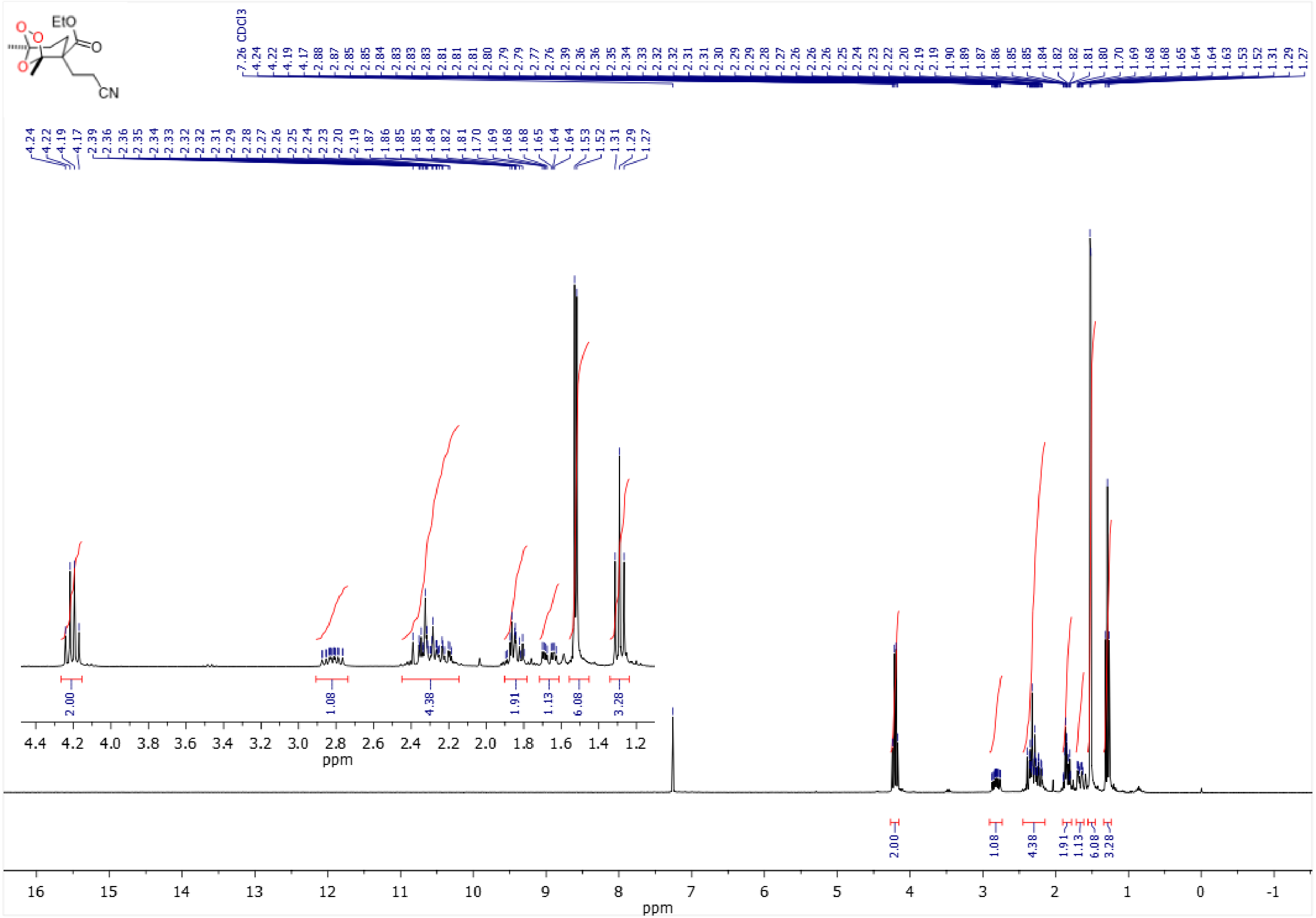

^{13}C NMR (75.48 MHz, $CDCl_3$). Ethyl (1*R**,2*R**,5*S**)-2-(2-cyanoethyl)-1,5-dimethyl-6,7,8-trioxabicyclo[3.2.1]octane-2-carboxylate, 3k

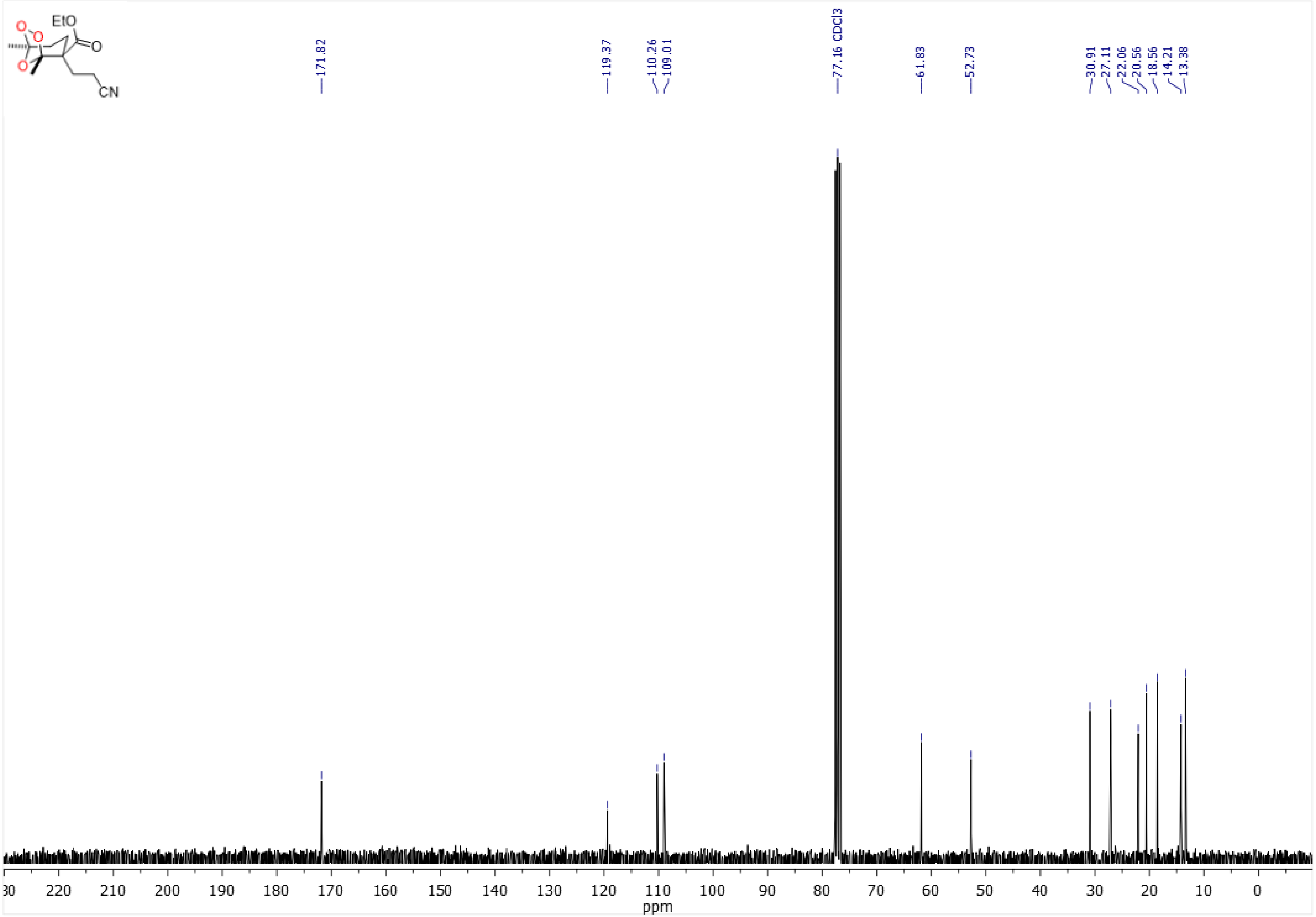

^{1}H NMR (300.13 MHz, CDCl3). (1*R,2*S**,5*S**)-2-(4-chlorobenzyl)-1,5-dimethyl-6,7,8-trioxabicyclo[3.2.1]octan-2-yl)methanol, 4**

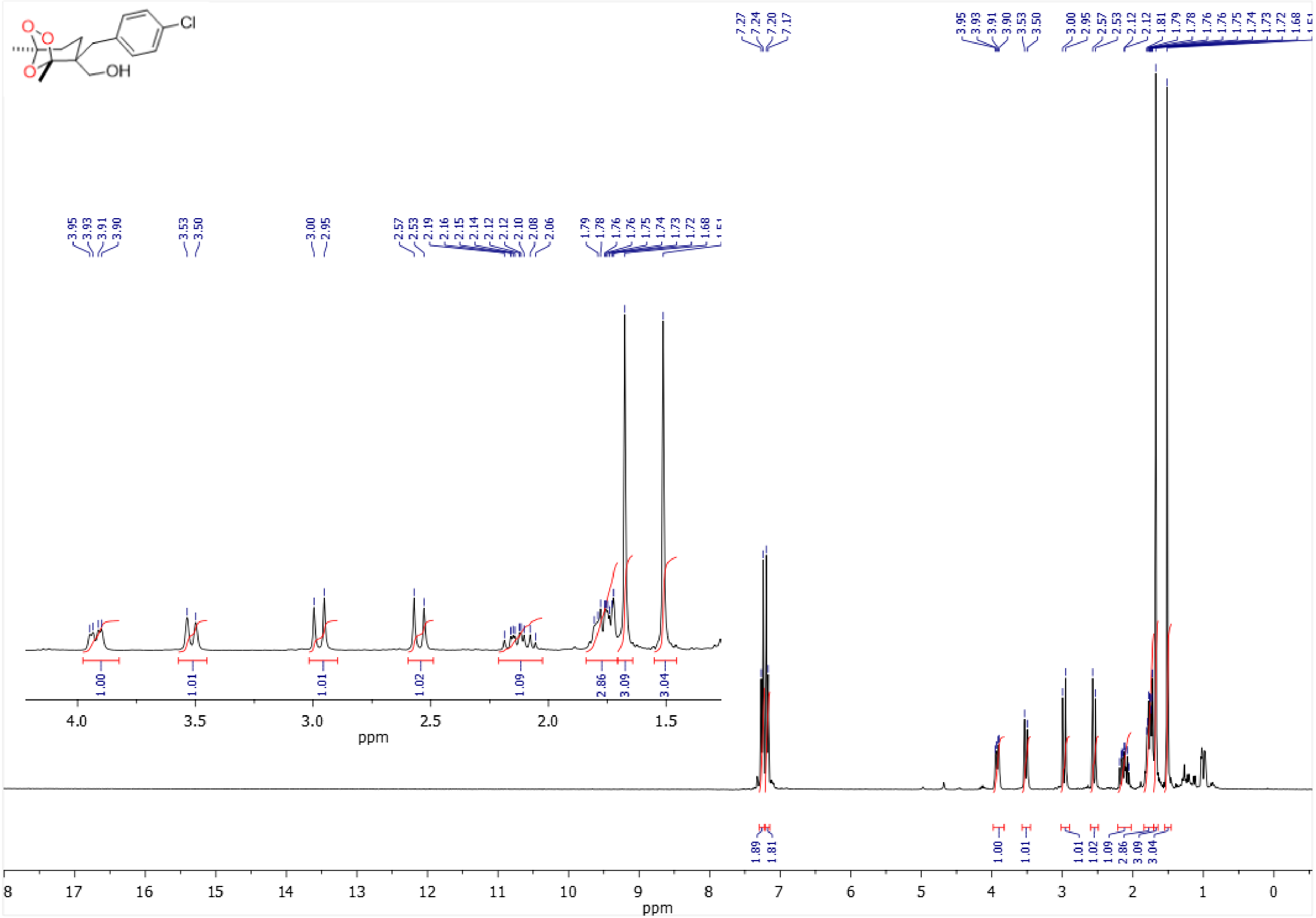

^{13}C NMR (75.48 MHz, CDCl₃). (1*R**,2*S**,5*S**)-2-(4-chlorobenzyl)-1,5-dimethyl-6,7,8-trioxabicyclo[3.2.1]octan-2-yl)methanol, 4

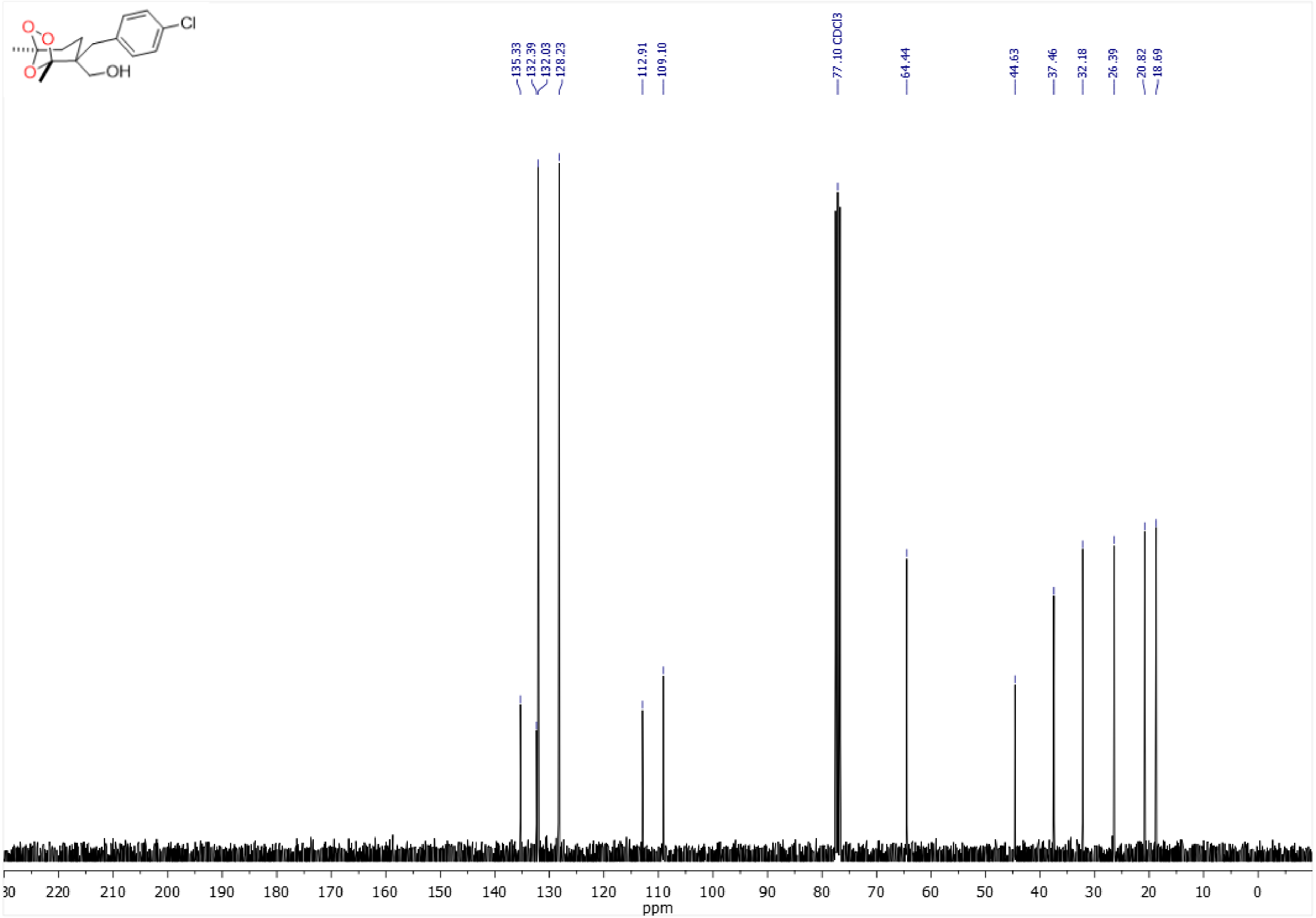

HRMS spectra of peroxide 4

(1*R,2*S**,5*S**)-2-(4-chlorobenzyl)-1,5-dimethyl-6,7,8-trioxabicyclo[3.2.1]octan-2-yl)methanol, 4**

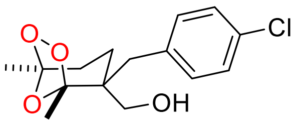

4

Display Report

Analysis Info

Analysis Name	D:\Data\Chizhov\Terentiev\Radulov\rd1052_&clblow.d	Acquisition Date	21.02.2020 16:25:21
Method	tune_low.m	Operator	BDAL@DE
Sample Name	/TERN Rd1052	Instrument / Ser#	micrOTOF 10248
Comment	CH3CN 100 %, dil. 200, calibrant added		

Acquisition Parameter

Source Type	ESI	Ion Polarity	Positive	Set Nebulizer	0.4 Bar
Focus	Not active			Set Dry Heater	180 °C
Scan Begin	50 m/z	Set Capillary	4500 V	Set Dry Gas	4.0 l/min
Scan End	3000 m/z	Set End Plate Offset	-500 V	Set Divert Valve	Waste

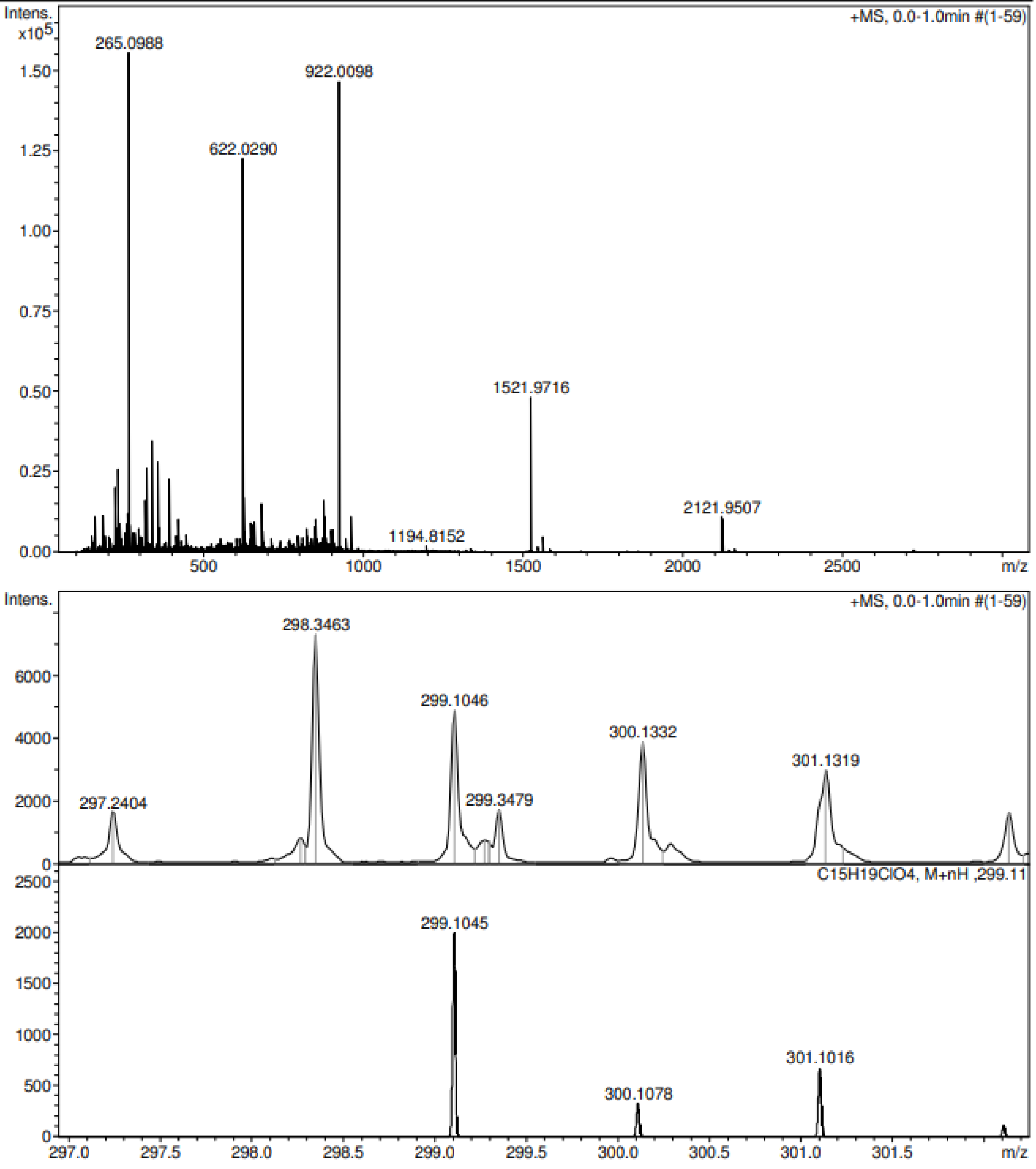

Bruker Compass DataAnalysis 4.0 printed: 21.02.2020 16:54:16 Page 1 of 1
